# Transcriptional Blood Signatures Distinguish Pulmonary Tuberculosis, Pulmonary Sarcoidosis, Pneumonias and Lung Cancers

**DOI:** 10.1371/journal.pone.0070630

**Published:** 2013-08-05

**Authors:** Chloe I. Bloom, Christine M. Graham, Matthew P. R. Berry, Fotini Rozakeas, Paul S. Redford, Yuanyuan Wang, Zhaohui Xu, Katalin A. Wilkinson, Robert J. Wilkinson, Yvonne Kendrick, Gilles Devouassoux, Tristan Ferry, Makoto Miyara, Diane Bouvry, Valeyre Dominique, Guy Gorochov, Derek Blankenship, Mitra Saadatian, Phillip Vanhems, Huw Beynon, Rama Vancheeswaran, Melissa Wickremasinghe, Damien Chaussabel, Jacques Banchereau, Virginia Pascual, Ling-pei Ho, Marc Lipman, Anne O’Garra

**Affiliations:** 1 Division of Immunoregulation, MRC National Institute for Medical Research, London, United Kingdom; 2 Department of Respiratory Medicine, Imperial College Healthcare NHS Trust, London, United Kingdom; 3 Baylor Institute for Immunology Research/ANRS Center for Human Vaccines, INSERM, Dallas, Texas, United States of America; 4 Division of Mycobacterial Research, MRC National Institute for Medical Research, London, United Kingdom; 5 Clinical Infectious Diseases Research Initiative, Institute of Infectious Diseases and Molecular Medicine, University of Cape Town, Cape Town, South Africa; 6 Department of Medicine, Wright Fleming Institute, Imperial College, London, United Kingdom; 7 MRC Human Immunology Unit, Weatherall Institute of Molecular Medicine, Oxford University, Oxford, United Kingdom; 8 Institut National de la Santé et de la Recherche Médicale (INSERM) U543, Immunologie A, Paris, France; 9 Service de Pneumologie, Hopital Avicenne, Paris, France; 10 Laboratoire AP-HP d’Immunologie Cellulaire et Tissulaire, Pitie Salpetriere Hospital, Paris, France; 11 Laboratoire de Biometrie et Biologie Evolutive, Equipe Epidémiologie et Santé Publique, Université de Lyon, Lyon, France; 12 Groupement Hospitalier Edouard Herriot, Service d’Hygiène, Epidémiologie et Prévention, Lyon, France; 13 Department of Respiratory Medicine, Royal Free London NHS Foundation Trust, London, United Kingdom; 14 Department of Respiratory Medicine, Barnet and Chase Hospital NHS Trust, London, United Kingdom; 15 Systems Immunology, Benaroya Research Institute, Seattle, Washington, United States of America; 16 University College London, London, United Kingdom; 17 Lyon-1 University CNRS UMR 5558, Lyon, France; 18 Service de Pneumologie, Hopital de la Croix-Rousse, Lyon, France; 19 Services des Maladies Infectieuses et Tropicales, Hopital de la Croix Rousse, Lyon, France; 20 Oxford Sarcoidosis Clinic, Oxford Centre for Respiratory Medicine, Churchill Hospital, Oxford, United Kingdom; 21 Baylor Institute for Health Care Research and Improvement, Baylor Health Care System, Dallas, Texas, United States of America; 22 Department of Rheumatology, Royal Free London NHS Foundation Trust, London, United Kingdom; 23 Division of Medicine, CRI, NHLI, Imperial College, London, United Kingdom; Emory University School of Medicine, United States of America

## Abstract

**Rationale:**

New approaches to define factors underlying the immunopathogenesis of pulmonary diseases including sarcoidosis and tuberculosis are needed to develop new treatments and biomarkers. Comparing the blood transcriptional response of tuberculosis to other similar pulmonary diseases will advance knowledge of disease pathways and help distinguish diseases with similar clinical presentations.

**Objectives:**

To determine the factors underlying the immunopathogenesis of the granulomatous diseases, sarcoidosis and tuberculosis, by comparing the blood transcriptional responses in these and other pulmonary diseases.

**Methods:**

We compared whole blood genome-wide transcriptional profiles in pulmonary sarcoidosis, pulmonary tuberculosis, to community acquired pneumonia and primary lung cancer and healthy controls, before and after treatment, and in purified leucocyte populations.

**Measurements and Main Results:**

An Interferon-inducible neutrophil-driven blood transcriptional signature was present in both sarcoidosis and tuberculosis, with a higher abundance and expression in tuberculosis. Heterogeneity of the sarcoidosis signature correlated significantly with disease activity. Transcriptional profiles in pneumonia and lung cancer revealed an over-abundance of inflammatory transcripts. After successful treatment the transcriptional activity in tuberculosis and pneumonia patients was significantly reduced. However the glucocorticoid-responsive sarcoidosis patients showed a significant increase in transcriptional activity. 144-blood transcripts were able to distinguish tuberculosis from other lung diseases and controls.

**Conclusions:**

Tuberculosis and sarcoidosis revealed similar blood transcriptional profiles, dominated by interferon-inducible transcripts, while pneumonia and lung cancer showed distinct signatures, dominated by inflammatory genes. There were also significant differences between tuberculosis and sarcoidosis in the degree of their transcriptional activity, the heterogeneity of their profiles and their transcriptional response to treatment.

## Introduction

Approximately nine million new cases of active tuberculosis (TB), and 1.4 million deaths from TB, are estimated to occur globally each year [1]. Prompt diagnosis is vital to avoid treatment delay, hence the ability to discriminate TB from other pulmonary conditions which can present similarly to TB, such as sarcoidosis, or have an acute (community acquired pneumonia) or chronic (primary lung cancer) presentation is important.

TB and sarcoidosis are widespread multisystem diseases that preferentially involve the lung and often present in a similar clinical, radiological and histological manner. Distinguishing these diseases therefore can require an invasive biopsy. Granuloma formation is fundamental to both conditions and although the pathogen *Mycobacterium tuberculosis* is recognised as the aetiological cause of TB, what underlies sarcoidosis is unknown [2]. The pathways involved in granulomatous inflammation are also poorly understood and there is little understanding of disease-specific differences. TB and sarcoidosis can also display a similar presentation to acute pulmonary infectious diseases such as community acquired pneumonia and chronic lung disorders such as primary lung cancer.

Given the complexity of these diseases a systems biology approach may help unravel the principal host immune responses. Peripheral blood has the capacity to reflect pathological and immunological changes elsewhere in the body, and identification of disease associated alterations can be determined by a blood transcriptional signature [3]. In support of this concept, we recently demonstrated an interferon (IFN)-inducible blood signature in patients with pulmonary TB from London and South Africa [4], which has now been validated in three independent studies in Africa [5], [6] and Indonesia [7]. Blood gene expression profiling has also been successfully applied to other infectious and inflammatory disorders, such as systemic lupus erythematosus (SLE), to help understand disease mechanisms and improve diagnosis and treatment [3]. Two recent studies have used blood transcriptional profiling for the comparison of pulmonary TB and sarcoidosis; both studies found the diseases had similar transcriptional responses, which involved the overexpression of IFN-inducible genes [8], [9]. However these studies did not examine other similar pulmonary diseases raising the question of whether these transcriptional signatures also reflected other pulmonary disorders.

The main objective of our study was to improve our understanding of the immunopathogenesis underlying sarcoidosis and TB by comparing the blood transcriptional responses in pulmonary TB patients to that found in pulmonary sarcoidosis, pneumonia and lung cancer patients. We also compared the blood transcriptional responses before and after treatment in each disease, and examined the transcriptional responses seen in the different leucocyte populations of the granulomatous diseases. In addition we investigated the association in sarcoidosis between clinical activity and the observed blood transcriptional heterogeneity.

## Methods

### Study Population and Inclusion Criteria

The majority of the TB patients were recruited from Royal Free Hospital NHS Foundation Trust, London. The sarcoidosis patients were recruited from Royal Free Hospital NHS Foundation Trust, St Mary’s Hospital Imperial College NHS Trust, Barnet and Chase Farm NHS Trust in London, the Oxford Sarcoidosis Clinic, Churchill Hospital in Oxford, and the Avicenne Hospital in Paris. The pneumonia patients were recruited from Royal Free Hospital, London. The lung cancer patients and 5 of the TB patients in the Test Set were recruited by the Lyon Collaborative Network, France. All patients were recruited consecutively over time such that the Training Set was recruited first followed by the Test Set, Validation Set and lastly the patients’ samples that were used in the cell purification. Additional blood gene expression data were obtained from pulmonary and latent TB patients recruited and analysed in our earlier study which were then re-analysed in this study when comparing responses before and after TB treatment [10].

The inclusion criteria were specific for each disease. Pulmonary TB patients: culture confirmed *Mycobacterium tuberculosis* in either sputum or bronchoalveolar lavage; pulmonary sarcoidosis: diagnosis made by a sarcoidosis specialist, granuloma’s on biopsy, compatible clinical and radiological findings (within 6 months of recruitment) according to the WASOG guidelines [11]; community acquired pneumonia patients: fulfilled the British Thoracic Society guidelines for diagnosis [12]; lung cancer patients: diagnosis by a lung cancer specialist, histological and radiological features consistent with primary lung cancer; healthy controls: their gender, ethnicity and age were similar to the patients, negative QuantiFERON-TB Gold In-Tube (QFT) (Cellestis) test. The exclusion criteria for all patients and healthy controls included significant other medical history (including any immunosuppression such as HIV infection), aged below 18 years or pregnant. Patients were recruited between September 2009 and March 2012. Patients were recruited before commencing treatment unless otherwise stated.

### Ethics Statement

This study was approved by the Central London 3 Research Ethics Committee (09/H0716/41) and CPP Sud-Est IV, France, CCPPRB, Pitié-salpétrière Hospital, Paris. All participants gave written informed consent.

### IFNγ Release Assay Testing

The QFT Assay (Cellestis) was performed according to the manufacturer’s instructions.

### Gene Expression Profiling

3ml of whole blood were collected into Tempus tubes (Applied Biosystems/Ambion) by standard phlebotomy, vigorously mixed immediately after collection, and stored between −20 and −80°C before RNA extraction. RNA was isolated using 1.5ml whole blood and the MagMAX-96 Blood RNA Isolation Kit (Applied Biosystems/Ambion) according to the manufacturer’s instructions. 250 µg of isolated total RNA was globin reduced using the GLOBINclear 96-well format kit (Applied Biosystems/Ambion) according to the manufacturer’s instructions. Total and globin-reduced RNA integrity was assessed using an Agilent 2100 Bioanalyzer (Agilent Technologies). RNA yield was assessed using a NanoDrop8000 spectrophotometer (NanoDrop Products, Thermo Fisher Scientific). Biotinylated, amplified antisense complementary RNA (cRNA) targets were then prepared from 200–250ng of the globin-reduced RNA using the Illumina CustomPrep RNA amplification kit (Applied Biosystems/Ambion). 750 ng of labelled cRNA was hybridized overnight to Illumina Human HT-12 V4 BeadChip arrays (Illumina), which contained more than 47,000 probes. The arrays were washed, blocked, stained and scanned on an Illumina iScan, as per manufacturer’s instructions. GenomeStudio (Illumina) was then used to perform quality control and generate signal intensity values.

### Cell Purification and RNA Processing for Microarray

Whole blood was collected in sodium heparin. Peripheral blood mononuclear cells (PBMCs) were separated from the granulocytes/erythrocytes using a Lymphoprep™ (Axis-Shield) density gradient. Monocytes (CD14+), CD4+ T cells (CD4+) and CD8+ T cells (CD8+) were isolated sequentially from the PBMCs using magnetic antibody-coupled (MACS) whole blood beads (Miltenyi Biotec, Germany) according to manufacturer’s instructions. Neutrophils were isolated from the granulocyte/erythrocyte layer after red blood cell lysis followed by CD15+ MACS beads (Miltenyi Biotec, Germany). RNA was extracted from whole blood (5′ Prime PerfectPure Kit) or separated cell populations (Qiagen RNeasy Mini Kit). Total RNA integrity and yield was assessed as described above. Biotinylated, amplified antisense complementary RNA (cRNA) targets were then prepared from 50 ng of total RNA using the NuGEN WT-Ovation™ RNA Amplification and Encore BiotinIL Module (NuGEN Technologies, Inc). Amplifed RNA was purified using the Qiagen MinElute PCR purification kit (Qiagen, Germany). cRNA was then handled as described above.

### Raw Data Processing

Raw data were processed using GeneSpring GX version 11.5 (Agilent Technologies) and the following was applied to all analyses. After background subtraction each probe was attributed a flag to denote its signal intensity detection *p*-value. Flags were used to filter out probe sets that did not result in a ‘present’ call in at least 10% of the samples, where the ‘present’ lower cut off = 0.99. Signal values were then set to a threshold level of 10, log2 transformed, and per-chip normalised using 75^th^ percentile shift algorithm. Next per-gene normalisation was applied by dividing each messenger RNA transcript by the median intensity of all the samples. All statistical analysis was performed after this stage. Raw microarray data has been deposited with GEO (Accession number GSE42834). All data collected and analysed in the experiments adhere to the Minimal Information About a Microarray Experiment (MIAME) guidelines.

### Data Analysis

GeneSpring 11.5 was used to select transcripts that displayed expression variability from the median of all transcripts (unsupervised analysis). A filter was set to include only transcripts that had at least twofold changes from the median and present in ≥10% of the samples. Unsupervised analysis was used to derive the 3422-transcripts. Applying a non-parametric statistical filter (Kruskal Wallis test with a FDR (Benjamini Hochberg) = 0.01), after the unsupervised analysis, generated the 1446-transcript and 1396-transcript signatures. The two signatures differed only in which groups the statistical filter was applied across; 1446, five groups (TB, sarcoidosis, pneumonia, lung cancer and controls) and 1396, six groups (TB, active sarcoidosis, non-active sarcoidosis, pneumonia, lung cancer and controls).

Differentially expressed genes for each disease were derived by comparing each disease to a set of controls matched for ethnicity and gender within a 10% difference. GeneSpring 11.5 was used to select transcripts that were ≥1.5 fold different in expression from the mean of the controls and statistically significant (Mann Whitney unpaired FDR (Benjamini Hochberg) = 0.01). Comparison Ingenuity Pathway Analysis (IPA) (Ingenuity Systems, Inc., Redwood, CA) was used to determine the most significant canonical pathways associated with the differentially expressed genes of each disease relative to the other diseases (Fisher’s exact FDR(Benjamini Hochberg) = 0.05). The bottom x-axis and bars of each comparison IPA graph indicated the log(p-value) and the top x-axis and line indicated the percentage of genes present in the pathway.

Molecular distance to health (MDTH) was determined as previously described [13], and then applied to different transcriptional signatures. Transcriptional modular analysis was applied as previously described [14]. The raw expression levels of all transcripts significantly detected from background hybridisation were compared between each sample and all the controls present in that dataset. The percentage of significantly expressed genes in each module were represented by the colour intensity (Student t-test, *p*<0.05), red indicates overexpression and blue indicates underexpression. The mean percentage of significant genes and the mean fold change of these genes compared to the controls in specified modules were also shown in graphical form. MDTH and modular analysis were calculated in Microsoft Excel 2010. GraphPad Prism version 5 for Windows was used to generate the graphs.

Differentially expressed genes between the Training Set TB patients and active sarcoidosis patients were derived using Significance Analysis of Microarrays (SAM) (*q*<0.05) and ≥1.5 fold expression change [15]. SAM was used due to the increased sensitivity of this non-parametric test compared to Mann-Whitney allowing more differentially expressed transcripts to be identified between TB and active sarcoidosis. Class prediction was performed within GeneSpring 11.5 using the machine learned algorithm support vector machines (SVM). The model was built using sample classifiers ‘TB’ or ‘not TB’. The SVM model should be built in one study cohort and run in an independent cohort to prevent over-fitting the predictive signature. This was possible for all the cohorts from our study. Where the study cohorts used a different microarray platform the SVM model had to be re-built in that cohort. To reduce the effects of over-fitting the same SVM parameters were always used. The kernel type used was linear, maximum iterations 100,000, cost 100, ratio 1 and validation type N-fold where N = 3 with 10 repeats. The receiver operating curve (ROC) and area under the curve (AUC) were calculated using Microsoft Excel 2010.

Univariate and multivariate regression analysis were calculated using STATA 9 (StataCorp 2005. Stata Statistical Software: Release 9. College Station, TX; StataCorp LP). In the multivariate regression analysis where there were missing data points (serum ACE and HRCT disease activity) to prevent list-wise deletion dummy variable adjustment was used.

The power calculations were conducted using the Power Analysis and Sample Size Software (PASS) 2008.

## Results

### Blood Transcriptional Profiles are Similar in the Pulmonary Granulomatous Diseases, TB and Sarcoidosis but Distinct from Pneumonia and Lung Cancer

Cohorts of TB and sarcoidosis, community acquired pneumonia and lung cancer patients, and healthy controls are shown in Table 1 and Figures S1–S3, and appropriately matched demographically and clinically (Tables S1-2). The study power was calculated for a training set (standard deviation = 0.4, 3,000 probes truly differentially expressed, fold change of ≥1.5, two sample t-test with FDR of 0.05). Eight to 40 patients per group provide a power of greater than 0.85 for each probe (calculated using PASS 2008) with minimal benefit of increasing the sample size.

**Table 1:** Summary of participant numbers in each cohort.

	Pulmonary Granulomatous Diseases		Other Pulmonary Diseases			TOTAL
	TB	Sarcoidosis	Pneumonia	Lung cancer	Healthy controls	
**Training Set**	16	25	8	8	38	95
**Test Set**	11	25	6	8	52	102
**Validation Set**	8	11			23	42
**TOTAL**	35	61	14	16	113	239

**Figure 1:**
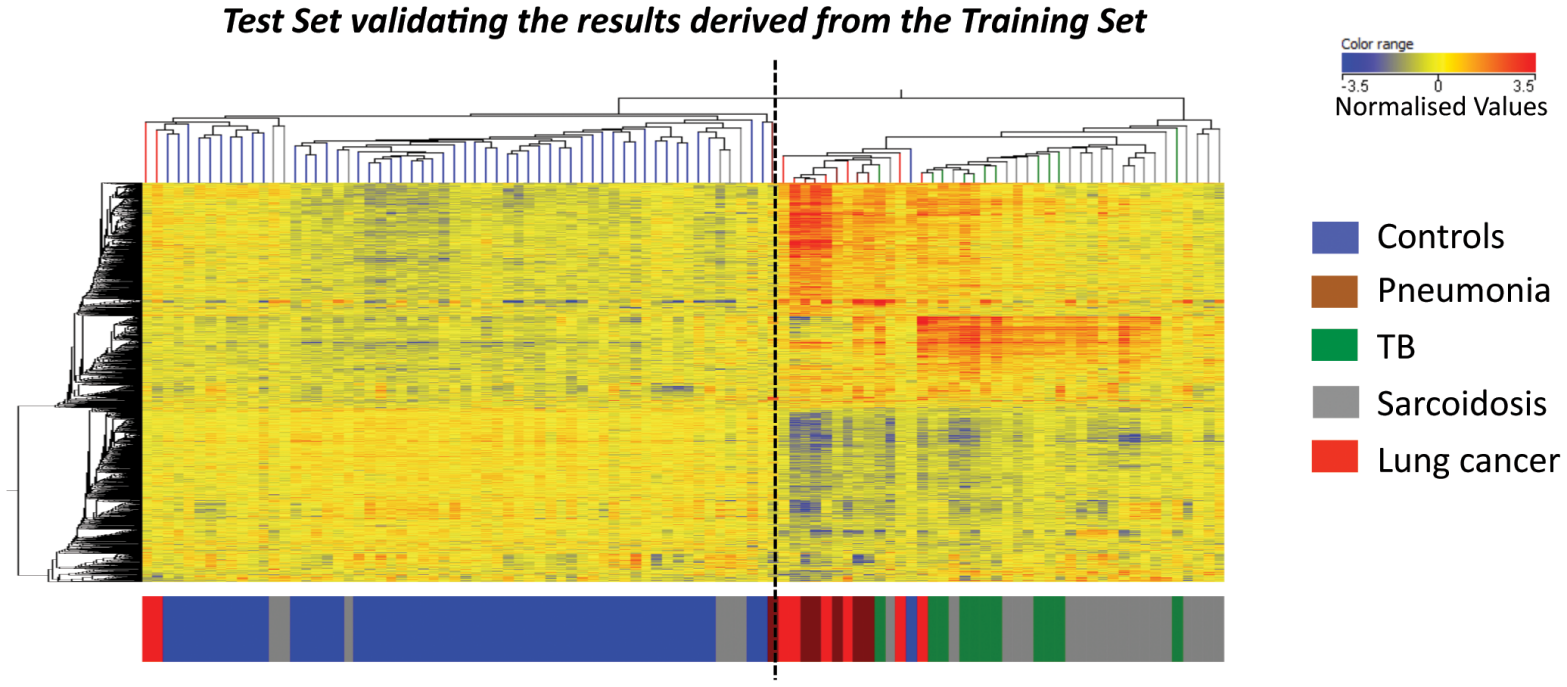
Pulmonary granulomatous diseases display similar transcriptional signatures that are distinct from pneumonia and lung cancer. 1446-transcripts were differentially expressed in the whole blood of the Training Set healthy controls, pulmonary TB patients, pulmonary sarcoidosis patients, pneumonia patients and lung cancer patients. The clustering of the 1446-transcripts were tested in an independent cohort from which they were not derived from, the Test Set. The heatmap shows the transcripts and Test Set patients’ profiles as organised by the unbiased algorithm of unsupervised hierarchical clustering. A dotted line is added to the heatmap to help visualisation of the main clusters generated by the clustering algorithm. Transcript intensity values are normalised to the median of all transcripts. Red transcripts are relatively over-abundant and blue transcripts under-abundant. The coloured bar at the bottom of the heatmap indicates to which group the profile belongs.

**Figure 2:**
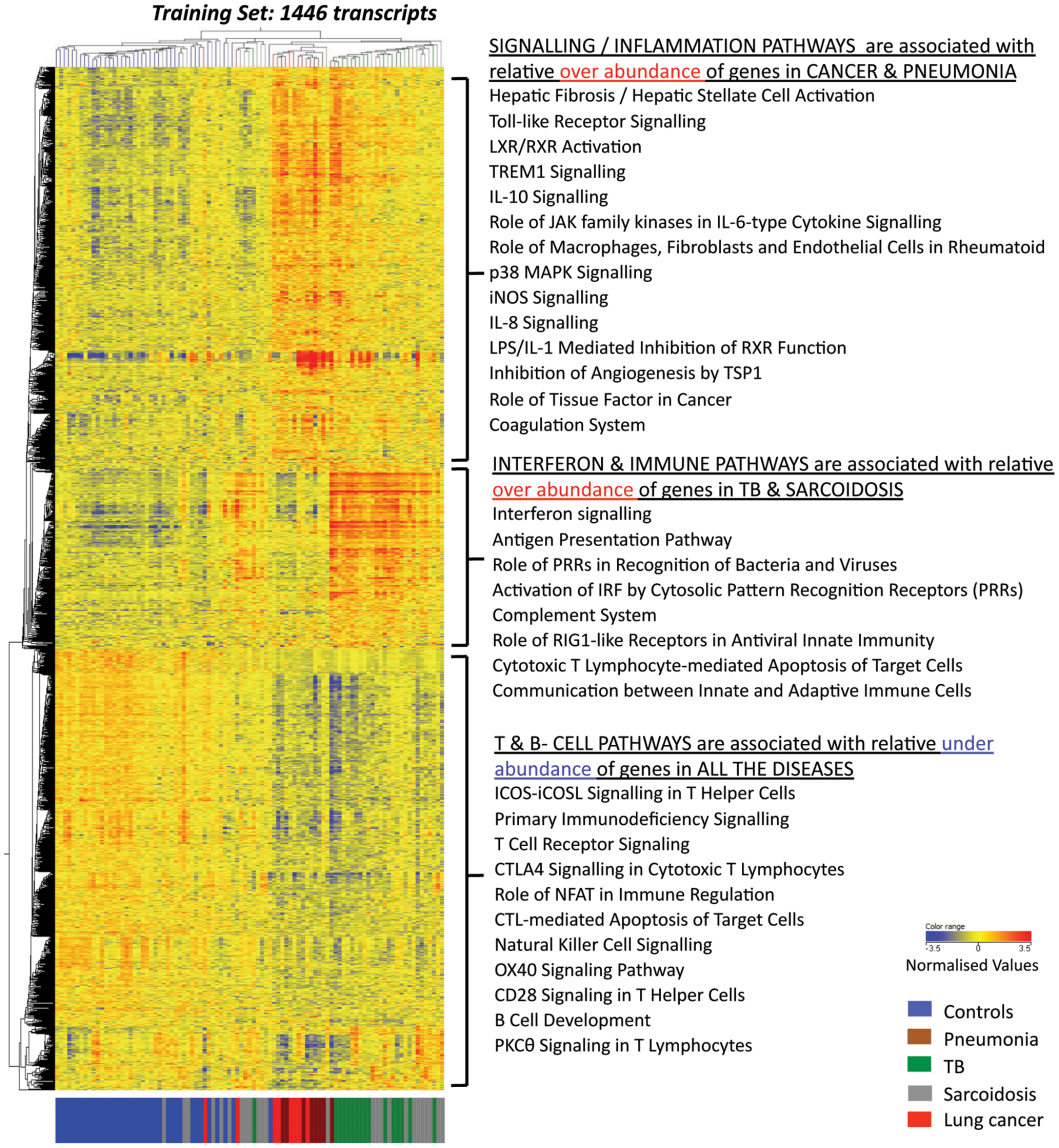
Three dominant clusters of the 1446 differentially expressed transcripts are associated with distinct biological pathways. Each of the three dominant clusters of transcripts is associated with different study groups in the Training Set. The top transcript cluster is over-abundant in the pneumonia and cancer patients and significantly associated with IPA pathways relating to inflammation (Fisher’s exact with Benjamini Hochberg FDR = 0.05). The middle transcript cluster is over-abundant in the TB and sarcoidosis patients and significantly associated with IFN signalling and other immune response IPA pathways (Fisher’s exact with Benjamini Hochberg FDR = 0.05). The bottom transcript cluster is under-abundant in all the patients and significantly associated with T and B cell IPA pathways (Fisher’s exact Benjamini Hochberg FDR = 0.05).

Unbiased analysis demonstrated that the TB and sarcoidosis blood transcriptional profiles clustered together but distinctly from pneumonia and cancer, which themselves clustered together (Training Set; 3422 transcripts, Figure S4A). Statistical filtering generated 1446 differentially expressed transcripts (Training Set, Figure S4B). This clustering pattern was verified in an independent cohort (Test Set, Figure 1), and was not influenced by ethnicity or gender (data not shown). Pathway analysis (IPA) revealed that the TB and sarcoidosis samples were associated with over-abundance of interferon (IFN) signalling and immune response pathways (Figure 2). Pneumonia and lung cancer samples were associated with over-abundance of pathways linked with inflammation. All diseases showed an under-abundance of T and B cell pathways.

### Heterogeneity of Transcriptional Profiles in Sarcoidosis Patients Correlates with Clinical Phenotype

**Figure 3:**
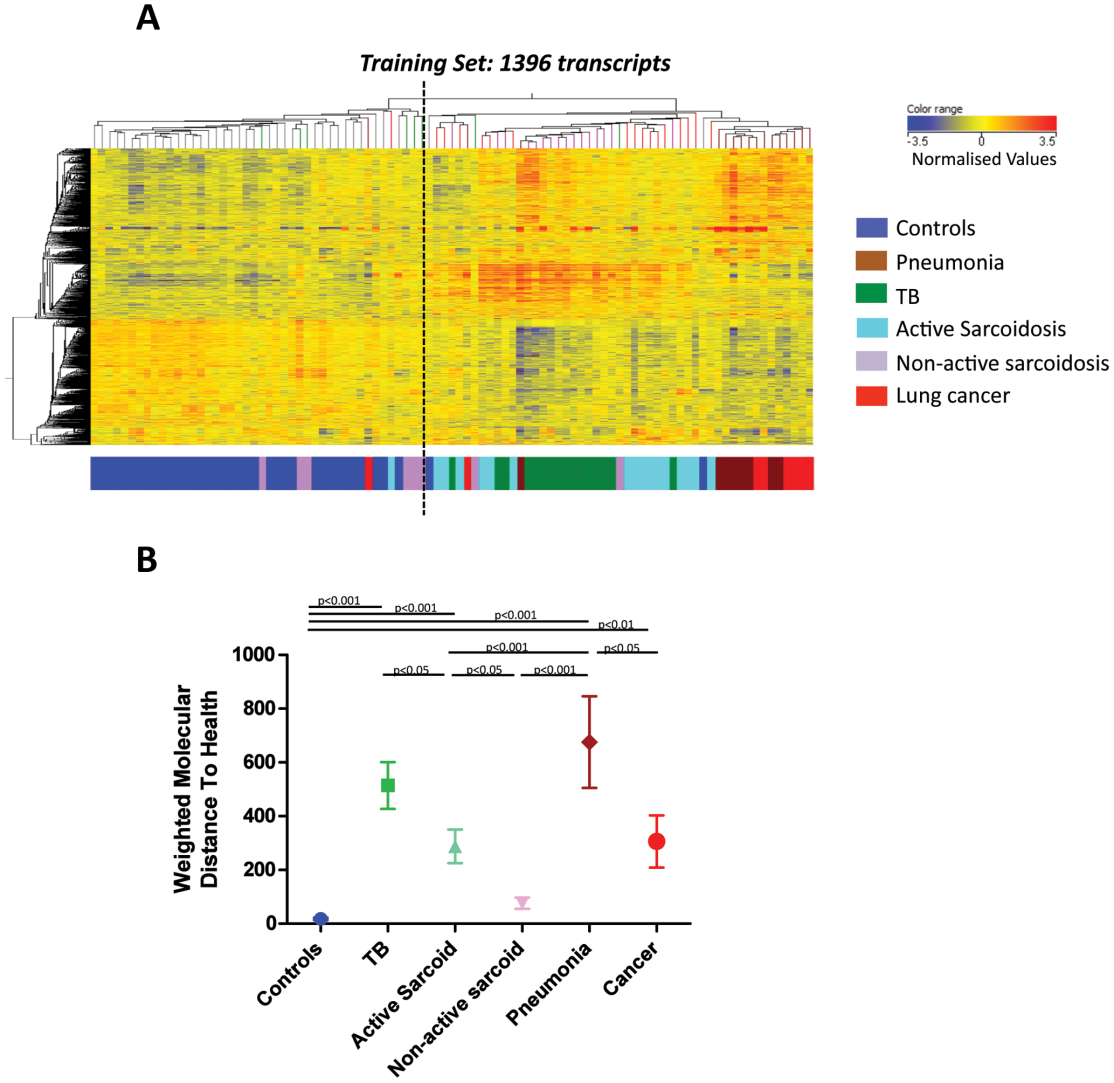
Active sarcoidosis signatures are similar to TB but distinct from non-sarcoidosis which resembles healthy controls. 1396-transcripts are differentially expressed in the whole blood of the Training Set after applying the analysis across six groups to include the two phenotypes of sarcoidosis patients. (A) The 1396 transcripts and Training Set patients’ profiles are organised by unsupervised hierarchical clustering. A dotted line is added to the heatmap to clarify the main clusters generated by the clustering algorithm. Transcript intensity values are normalised to the median of all transcripts. (B) Molecular distance to health of the 1396 transcripts in the Training and Test sets demonstrates the quantification of transcriptional change relative to the controls. The mean, SEM and *p-*values are displayed (ANOVA with Tukey’s multiple comparison test).

Transcriptional profiles of the sarcoidosis patients clustered either with TB patients or with healthy controls (1446 transcripts, Figure 1). Patients were thus assessed for disease activity (labelled as either active or non-active) by a pre-defined decision tree of a composite of clinical parameters reflecting a snapshot profile of disease activity at that time-point (Figure S5; Table S3). 1396-transcripts were found to be differentially expressed using this sarcoidosis classification. Again active sarcoidosis patients clustered with the TB patients, whereas non-active sarcoidosis patients clustered with the controls (1396 transcripts, Figure 3A). This was validated in the independent cohorts Test and Validation Sets (Figure S6A & B; Table S4A, S4B).

Application of the Molecular distance to health (MDTH) algorithm [13] to all the disease groups showed that the non-active sarcoidosis MDTH score was not quantitatively significantly different from the controls, whereas the active sarcoidosis MDTH score was (Figure 3B). The TB scores were significantly higher than the active sarcoidosis scores, and the pneumonia scores were significantly higher than the lung cancer scores, with pneumonia and TB having the highest MDTH scores. Collectively, this suggests a quantitative as well as qualitative difference in blood transcriptional signatures.

### Three Data Mining Strategies Show that TB and Active Sarcoidosis are Dominated by IFN-inducible Genes, Whereas Pneumonia and Lung Cancer are Dominated by Inflammatory Genes

**Figure 4:**
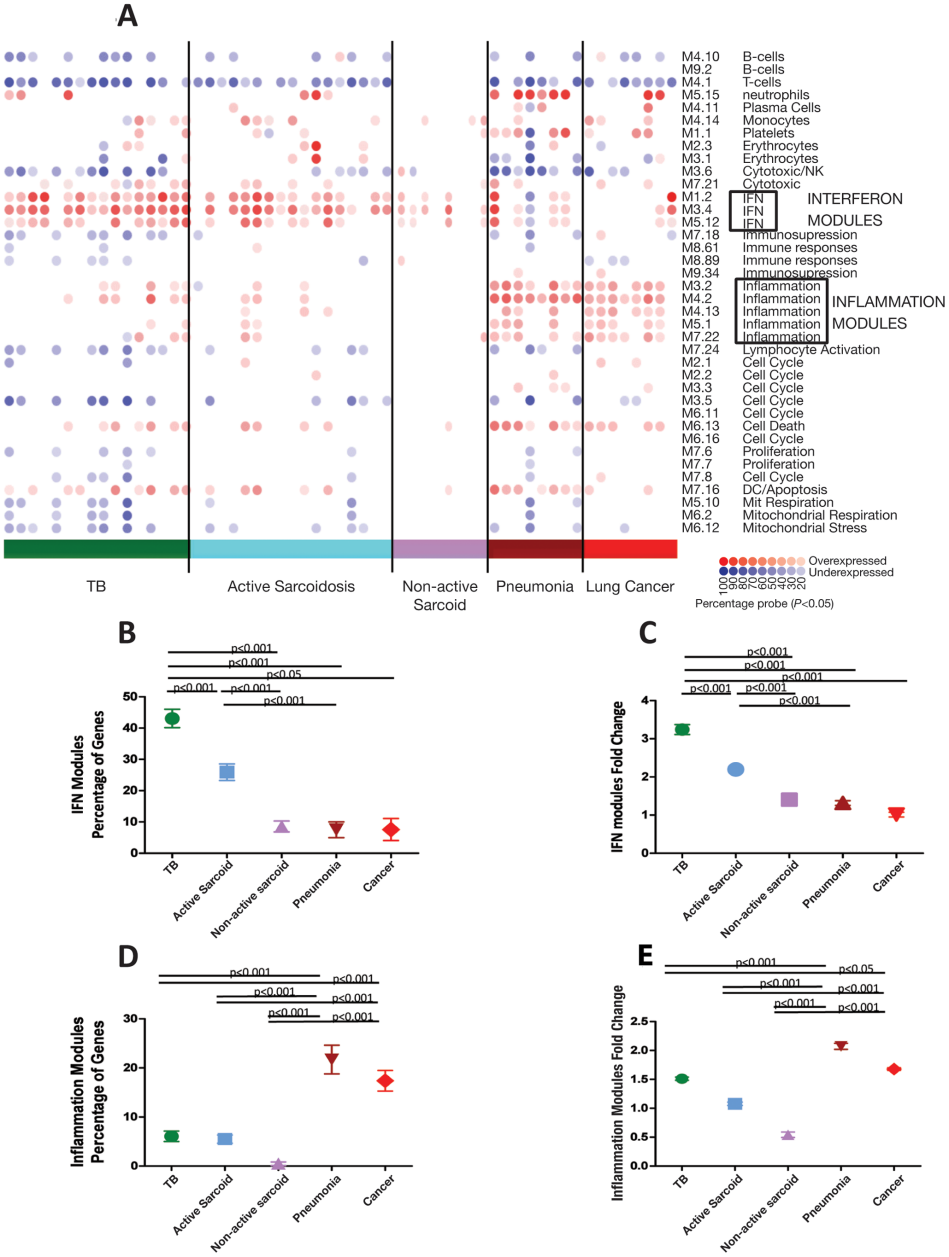
Modular analysis shows similar pathways associated with TB and sarcoidosis, differing from pneumonia and cancer. (A) Gene expression levels of all transcripts that were significantly detected compared to background hybridisation (15212 transcripts, *p*<0.01) were compared in the Training Set between each patient group: TB, active sarcoidosis, non-active sarcoidosis, pneumonia, lung cancer, to the healthy controls. Each module corresponds to a set of co-regulated genes that were assigned biological functions by unbiased literature profiling. A red dot indicates significant over-abundance of transcripts and a blue dot indicates significant under-abundance (p<0.05). The colour intensity correlates with the percentage of genes in that module that are significantly differentially expressed. The modular analysis can also be represented in graphical form as shown in (B)–(E), including both the Training and Test Set samples. The mean, SEM and *p-*values are displayed (ANOVA with Tukey’s multiple comparison test). (B) The percentage of genes significantly overexpressed in the 3 IFN modules for each disease. (C) The fold change of the expression of the genes present in the IFN modules compared to the controls. (D) The percentage of genes significantly overexpressed in the 5 inflammation modules for each disease. (E) The fold change of the expression of the genes present in the inflammation modules compared to the controls.

We further investigated the biological pathways associated with each disease group, using modular analysis, [14], IPA, and annotation of the top differentially expressed genes for each disease group. Modular analysis, which takes into account all genes differentially expressed against controls, verified that TB and active sarcoidosis showed significant overexpression of the IFN modules compared to the other pulmonary disease groups (Figure 4A; Training Set; Figure S7, Test Set), whereas pneumonia and cancer patients showed significant overexpression of the inflammation modules (Figure 2). TB patients showed a significant quantitative increase in the number of IFN-inducible genes and their degree of expression, compared to the active sarcoidosis patients (Figures 4B, 4C). Pneumonia and lung cancer showed a significant increase in the number of genes present in the inflammation modules and their degree of expression, in comparison to TB and active sarcoidosis (Figures 4D, 4E). Pneumonia patients showed an increased number of genes present in the neutrophil module (Figure S8), correlating with blood neutrophil count (Spearman’s correlation, r = 0.42, *p*<0.0001).

**Figure 5:**
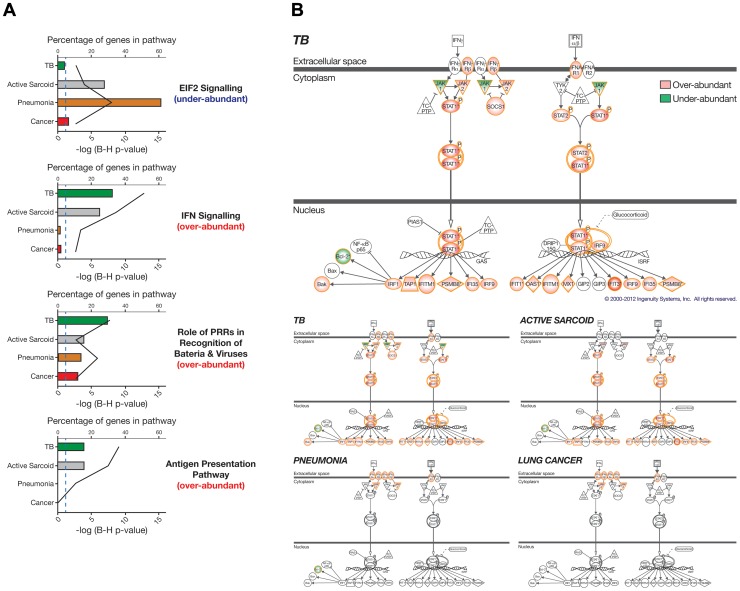
Comparison analysis of the diseases compared to matched controls reveals the four most significant pathways. Differentially expressed genes were derived from the Training Set by comparing each disease to healthy controls matched for ethnicity and gender: TB = 2524, active sarcoidosis = 1391, pneumonia = 2801 and lung cancer = 1626 transcripts (≥1.5 fold change from the mean of the controls, Mann Whitney with Benjamini Hochberg FDR = 0.01). (A) IPA canonical pathways was used to determined the most significant pathways (i-iv) associated with each disease relative to the other diseases (Fisher’s exact with Benjamini Hochberg FDR = 0.05). The bottom x-axis and bars of each graph indicates the log (p-value) and the top x-axis and line indicates the percentage of genes present in the pathway. The genes in the EIF2 signalling pathway are predominately under-abundant genes however the genes in the other three pathways are predominantly over-abundant relative to the controls. Pathways above the blue dotted line are significant (p<0.05). (B) The IFN signalling IPA pathway is overlaid onto each disease group. Coloured genes are differentially expressed in that disease group compared to their matched controls (Fisher’s exact FDR = 0.05). Red genes represent over-abundance and green under-abundance. The pathway for TB is shown enlarged so the detail of the genes can be seen, it is also shown again in a much smaller scale besides the other diseases so that a visual comparison can be more easily made.

Comparison IPA, using genes that were differentially expressed between each disease group and a matched set of controls, revealed the most significant pathways when comparing across the diseases (≥1.5 fold change from the controls, Mann Whitney Benjamini Hochberg *p*<0.01; TB = 2524, active sarcoidosis = 1391, pneumonia = 2801 and lung cancer = 1626 transcripts). The top four significant pathways were related to protein synthesis (EIF2 signalling), the immune response, IFN signalling, pattern recognition receptors recognising bacteria and viruses, and antigen presentation (Figure 5A and Table S11). Under-abundance of the EIF2 signalling pathway was driven by the pneumonia patients. Other genes related to protein synthesis (ribosomal proteins and other eukaryotic initiation factors) and the unfolded protein response (a stress response to excessive protein synthesis), were also significantly under-abundant in the pneumonia patients e.g. PERK, CHOP, ABCE1 (data not shown). The statistical significance (bottom x-axis, bar graph, Figure 5A) and percentage of genes (top x-axis, line graph, Figure 5A) of the three immune response pathways were driven predominantly by the TB and active sarcoidosis patients, again demonstrating the similarity of the biological pathways underlying these diseases. However the IFN-signalling pathway was more significant (Figure 5A) and contained a higher number of genes in TB than active sarcoidosis (Figure 5B).

The top 50 over-abundant differentially expressed transcripts for each disease correlated with the findings from the modular and IPA analysis where TB and active sarcoidosis were dominated by IFN-inducible genes e.g. IFITM3, IFIT3, GBP1, GBP6, CXCL10, OAS1, STAT1, IFI44L, FCGR1B (Table S5). Again these were greater in number in TB than sarcoidosis. The top 50 over-abundant transcripts in pneumonia were dominated by antimicrobial neutrophil-related genes e.g ELANE, DEFA1B, MMP8, CAMP, DEFA3, DEFA4, MPO, LTF. The genes FCGR1A, B and C were over-abundant in the top 50 transcripts of all four pulmonary diseases. A 4-set Venn diagram of the differentially expressed genes demonstrated unique genes for each disease group (Figure S9 and Table S6), with over three times the number of unique TB genes than unique active sarcoidosis genes. The TB unique genes comprised immune responses genes significantly associated with the IFN-signalling pathway and antigen presentation. The unique pneumonia genes were associated with an under-abundance of pathways related to protein synthesis. The unique lung cancer genes were associated with over-abundance of inflammation related pathways and under-abundance of T cell pathways. Overlapping genes common to all four disease groups were significantly associated with under-abundance of T and B cell pathways.

### TB and Pneumonia Patients after Treatment Showed a Diminishment of Transcriptional Profiles Whereas Sarcoidosis Patients Responding to Glucocorticoids Showed a Significant Increase in Transcriptional Activity

**Figure 6:**
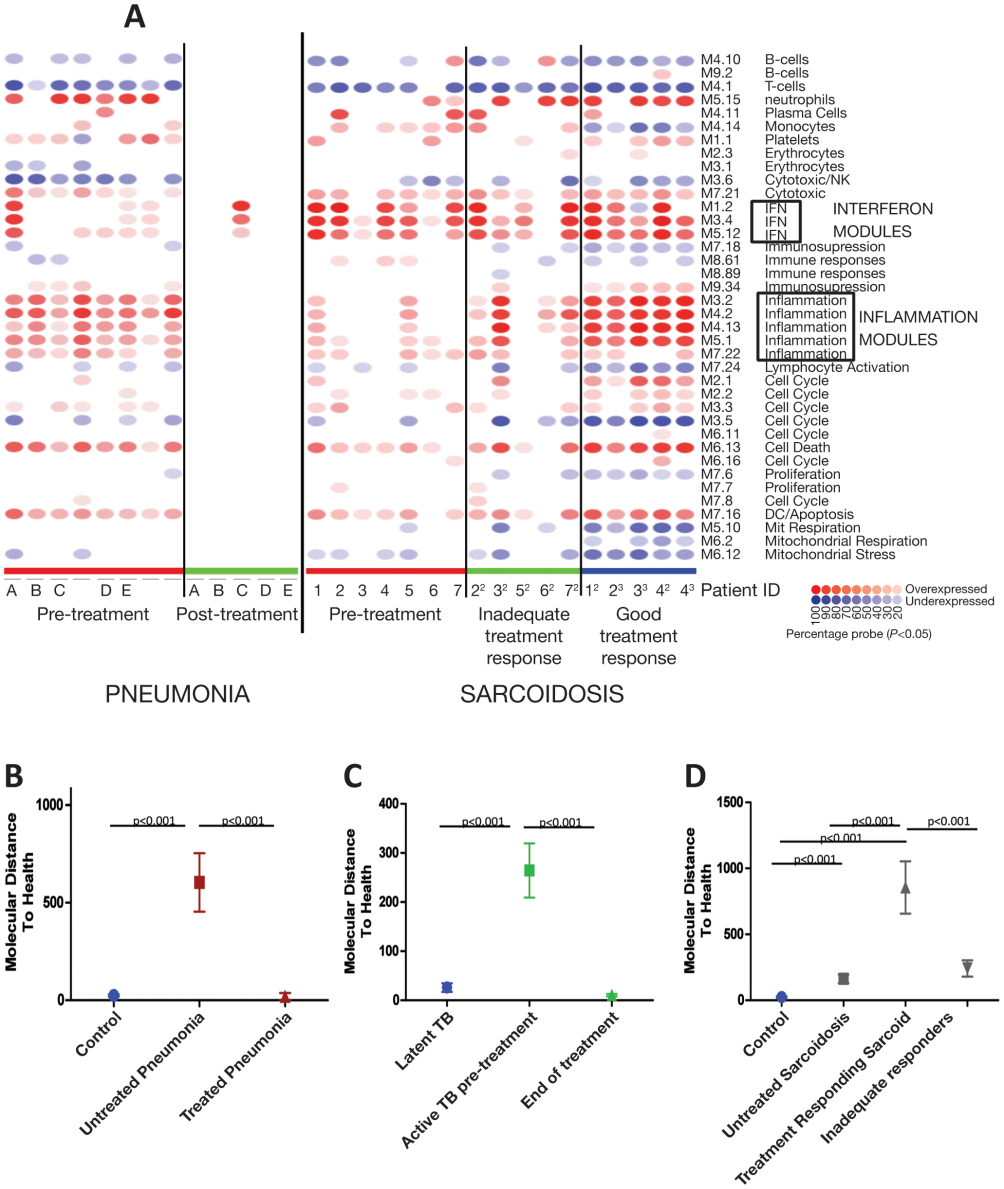
Unlike TB and pneumonia, successful treatment of sarcoidosis was associated with increased transcriptional activity. (A) Modular analysis. Gene expression levels of all transcripts that were significantly detected compared to background hybridisation in at least 10% of samples (p<0.01) were compared between the healthy controls and each of the following the patient groups: pre-treatment pneumonia, post-treatment pneumonia patients and pre-treatment sarcoidosis, inadequate treatment response sarcoidosis and good-treatment response sarcoidosis patients. A red dot indicates significant over-abundance of transcripts and a blue dot indicates under-abundance (p<0.05). The colour intensity correlates to the percentage of genes in that module that are significantly differentially expressed. MDTH demonstrates the quantification of transcriptional change after treatment in the 1446-transcripts relative to controls. The mean, SEM and p-values are displayed (ANOVA with Tukey’s multiple comparison test). (B) Pneumonia patients. (D) Sarcoidosis patients. (C) TB patients from the Bloom et al study carried out in South Africa, the controls in this study were participants with latent TB.

We next examined the transcriptional response to treatment. Pneumonia patients, followed-up at least 6 weeks after their hospital discharge, showed a good clinical response to standard antibiotic treatment (Table S7A) and a diminishment in their transcriptional profiles to the level of controls by modular analysis (all detectable transcripts) and MDTH (1446-transcripts) (Figure 6A & 6B). The MDTH of the 1446-transcripts derived in the present study of different lung diseases, was also diminished in the blood of South African TB patients upon completion of treatment, reverting to the signature of latent TB controls (Figure 6C) and supporting our previous report [10].

Sarcoidosis patients showed a variable clinical response after initiation of immunosuppressive treatment as determined by their practising physician (Table S7B); if their treatment was increased on clinic follow-up the patient was categorised as having an ‘inadequate treatment response’; continuing the same treatment or reducing treatment categorised the patient as having a ‘good treatment response’ (Table S7B). Sarcoidosis patients with a ‘good treatment response’ showed a significantly increased transcriptional activity in inflammatory transcripts although IFN-inducible transcripts remained unchanged, which was not seen in sarcoidosis patients with an ‘inadequate treatment response’ (Figure 6A & 6B). The top 50 overexpressed inflammatory genes in the ‘good treatment response’ sarcoidosis patients included anti-inflammatory genes e.g IL1R2, DUSP1, IL18R, C-FOS, IκBα and MAPK1 (Table S8).

### IFN-inducible Genes are Most Abundant in Neutrophils in Both TB and Sarcoidosis

**Figure 7:**
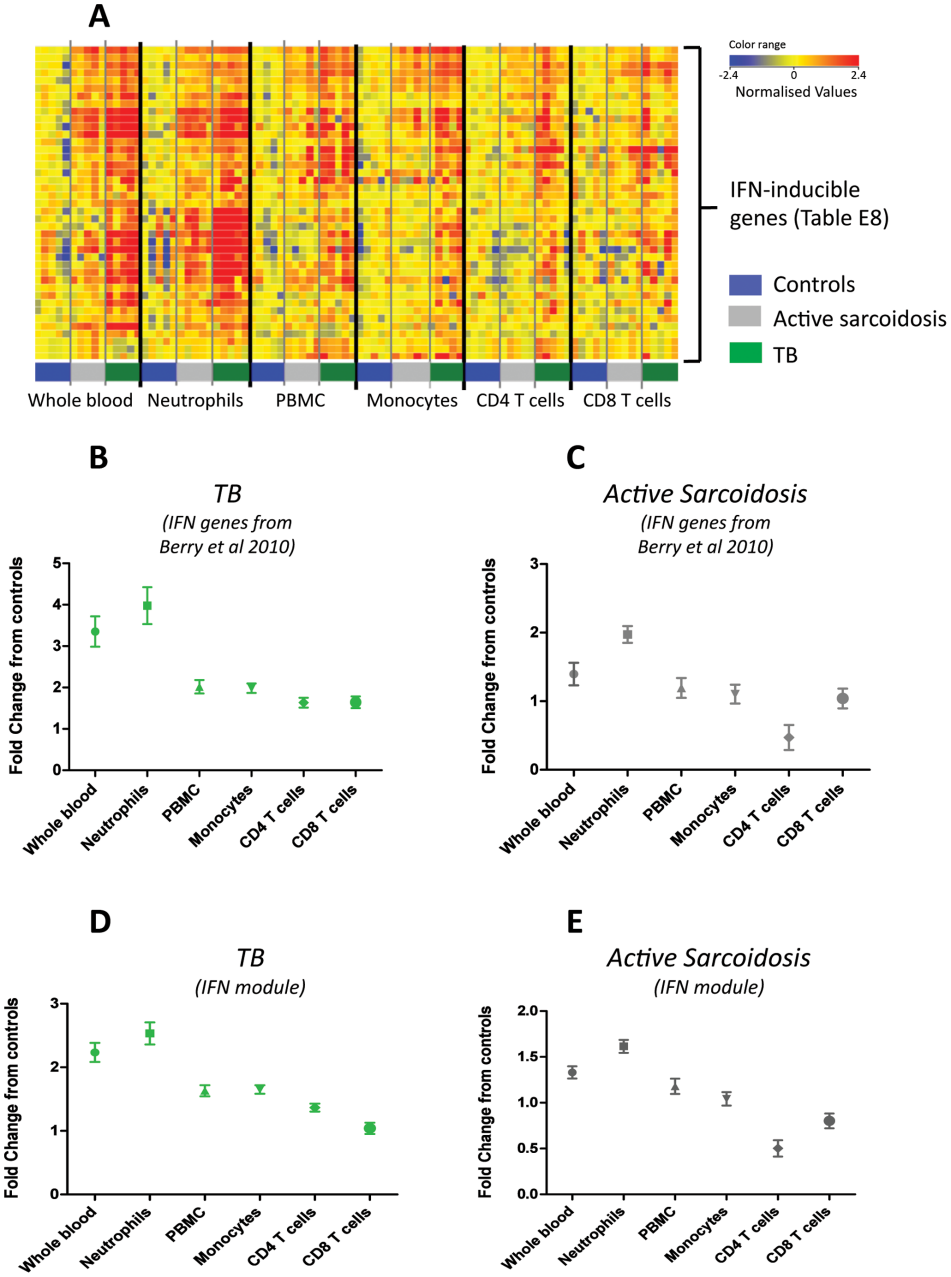
IFN-inducible gene expression is most abundant in the neutrophils in both TB and sarcoidosis. The expression of IFN-inducible genes were measured in purified leucocyte populations from whole blood. (A) Heatmap shows the expression of IFN-inducible transcripts, from the Berry et al 2010 study, for each disease group normalised to the controls for that cell type. (B) The mean expression fold change in the TB samples of the same IFN-inducible transcripts. (C) The mean expression fold change in the sarcoidosis samples of the same IFN-inducible transcripts. (D) The mean expression fold change in the TB samples of all the genes present in the three IFN modules compared to the controls. (E) The mean expression fold change in the sarcoidosis samples of all the genes present in the three IFN modules compared to the controls. Graphs show mean and SEM.

Analysis of purified blood cell populations revealed that neutrophils displayed the highest abundance of IFN-inducible genes (Figure 7A, B & D; Tables S9 & 10); and monocytes showed a higher abundance of IFN-inducible genes than lymphocytes in sarcoidosis as well as TB patients (Figure 7A-E). The findings in the TB patients were in keeping with our earlier study [4].

### TB Patients Can be Distinguished from the Other Pulmonary Diseases and Healthy Controls

Comparison of the TB transcriptional profiles to the most similar group, active sarcoidosis, revealed that 144 transcripts were differentially expressed, including IFN-inducible genes (Table 2; Training Set; significance analysis of microarray *q*<0.05, fold change ≥1.5). Using the machine learning algorithm, support vector machines (SVM), these 144 transcripts showed good sensitivity (above 80%) and specificity (above 90%), in all three independent cohorts from our own study (Training, Test and Validation Sets) and also when tested in an external cohort from the Maertzdorf *et al* study [9] (Table 3, Figures S10 & S11a). However, 76 of 100 transcripts, recognised as genes by NIH DAVID Gene ID Conversion Tool, proposed by Maertzdorf *et al*. to distinguish TB and sarcoidosis, showed a much lower sensitivity (45–56%), although similar specificity (above 90%), when tested in our three independent cohorts (Table 4 & Figure S11b). Fifty genes shown to be differentially expressed in TB and sarcoidosis by Koth *et al.* [8] also resulted in lower sensitivity (75–45%), although similar specificity (above 87%), when tested in our three independent cohorts (Table 5 & Figure S11c). It is possible class prediction was affected by the different microarray platforms used in each study; however, the external validation of our 144 transcripts (Illumina) in the Maertzdorf *et al* study (Agilent) provides further evidence for the discriminative accuracy of the 144 transcripts. There were few overlapping transcripts between our study, the Maertzdorf *et al* study and the Koth *et al* study; this may reflect the lack of validation of the transcript lists derived in both the Maertzdorf and Koth *et al* studies (Figure S10).

**Table 2:** 144 transcripts.

Symbol	FC	Exp.	Symbol	FC	Exp.	Symbol	FC	Exp.
C1QB	10.6	UP	LOC653610	1.9	UP	PLAC8	1.6	UP
LOC100133565	6.4	UP	CST7	1.9	UP	BAGE5	1.6	UP
TDRD9	5.3	UP	LILRB4	1.9	UP	DUSP3	1.6	UP
ABCA2	5.3	UP	MSL3L1	1.9	UP	SLC22A4	1.6	UP
SMARCD3	5.3	UP	HIST1H2BG	1.9	UP	LOC645159	1.6	UP
CACNA1E	5.1	UP	OSM	1.9	UP	IL4R	1.6	UP
HP	4.2	UP	LILRA5	1.9	UP	FLJ32255	1.6	UP
NTN3	4.2	UP	GPR97	1.9	UP	HIST2H2AA3	1.6	UP
LOC100008589	3.3	UP	HIST2H2AC	1.9	UP	PLAC8	1.6	UP
CARD17	3.3	UP	LILRA5	1.8	UP	SH3GLB1	1.6	UP
LOC441763	3.2	UP	TLR5	1.8	UP	PLSCR1	1.6	UP
ERLIN1	3.1	UP	LOC728417	1.8	UP	IFI35	1.6	UP
SLPI	3.1	UP	MSL3	1.8	UP	TAOK1	1.6	UP
SLC26A8	2.9	UP	HPSE	1.8	UP	MCTP1	1.6	UP
AIM2	2.8	UP	RGL4	1.8	UP	CEACAM1	1.6	UP
INCA	2.8	UP	CYP1B1	1.8	UP	B4GALT5	1.6	UP
OPLAH	2.7	UP	HIST2H2AA3	1.8	UP	COP1	1.6	UP
LPCAT2	2.6	UP	AGTRAP	1.8	UP	PROK2	1.6	UP
Sep-04	2.5	UP	PFKFB3	1.8	UP	IFI30	1.6	UP
DISC1	2.5	UP	GNG8	1.8	UP	FCER1G	1.5	UP
ZFP91	2.5	UP	LTB4R	1.8	UP	ZNF438	1.5	UP
UBE2J2	2.4	UP	H2AFJ	1.8	UP	EEF1D	1.5	UP
KREMEN1	2.4	UP	LILRA5	1.8	UP	MIR21	1.5	UP
ALPL	2.3	UP	ABCA1	1.8	UP	NGFRAP1	1.5	UP
LOC100008589	2.3	UP	SULT1B1	1.8	UP	PGS1	1.5	UP
KCNJ15	2.2	UP	GYG1	1.7	UP	KIF1B	1.5	UP
C19orf59	2.2	UP	IFITM1	1.7	UP	C16orf57	1.5	UP
FCGR1A	2.2	UP	SVIL	1.7	UP	ANKRD33	1.5	UP
SPATA13	2.2	UP	DGAT2	1.7	UP	MXD4	−1.5	DOWN
ADM	2.2	UP	MEFV	1.7	UP	ZSCAN18	−1.6	DOWN
CDK5RAP2	2.2	UP	PIM3	1.7	UP	MEF2D	−1.6	DOWN
SNORA73B	2.2	UP	MTRF1L	1.7	UP	BHLHB2	−1.7	DOWN
TncRNA	2.1	UP	MAZ	1.7	UP	CLC	−2.3	DOWN
PPAP2C	2.1	UP	HIST2H2AA4	1.7	UP	FCER1A	−2.5	DOWN
IFITM3	2.1	UP	LOC728519	1.7	UP	SRGAP3	−2.6	DOWN
FCGR1B	2.1	UP	SMARCD3	1.7	UP	FLJ43093	−2.8	DOWN
JMJD6	2.1	UP	LOC641710	1.7	UP	CCR3	−2.9	DOWN
HIST1H3D	2.1	UP	HIST2H2BE	1.7	UP	EMR4	−3	DOWN
LMNB1	2	UP	ITPRIPL2	1.7	UP	ZNF792	−3.1	DOWN
S100A12	2	UP	FKBP5	1.7	UP	C10orf33	−3.5	DOWN
FCGR1C	2	UP	IFNAR1	1.6	UP	CACNG6	−3.8	DOWN
LOC653591	2	UP	LY96	1.6	UP	P2RY10	−4.2	DOWN
LOC100132394	2	UP	GPR109A	1.6	UP	GATA2	−4.6	DOWN
SLC26A8	2	UP	DHRS13	1.6	UP	EMR4P	−6.6	DOWN
ANXA3	2	UP	IL18R1	1.6	UP	ESPN	−7	DOWN
NLRC4	1.9	UP	GPR109B	1.6	UP	EMR4	−9.3	DOWN
LILRA6	1.9	UP	AGTRAP	1.6	UP			

The 144 transcripts are differentially expressed genes between the TB and active sarcoidosis profiles in the Training Set (significance analysis of microarray *q*<0.05, fold change ≥1.5). FC = Fold change TB versus active sarcoidosis. Exp. = Regulation of expression.

**Table 3:** Class prediction of 144 transcripts from the Training Set.

	Present study Training SetTB vs non-TB (controls, sarcoid, cancer, pneumonia)	Present study Test SetTB vs non-TB (controls, sarcoid, cancer, pneumonia)	Present study Validation SetTB vs non-TB (controls &sarcoid)	Maertzdorf et alTB vs non-TB (controls & sarcoid)
**Sensitivity**	88%	82%	88%	88%
**Specificity**	94%	91%	92%	97%

Class prediction was performed using support vector machines (SVM). The 144 transcripts derived from the Training Set were used to build the SVM model, the model was then run in the other two cohorts and the external cohort (Maertzdorf *et al*).

**Table 4:** Class prediction of 100 Agilent transcripts from the Maertzdorf et al study.

	Present study Training SetTB vs non-TB (controls, sarcoid, cancer, pneumonia)	Present study Test SetTB vs non-TB (controls, sarcoid, cancer, pneumonia)	Present study Validation Set TB vs non-TB(controls & sarcoid)	Maertzdorf et alTB vs non-TB(controls & sarcoid)
**Sensitivity**	56%	45%	75%	88% (as stated in their publication)
**Specificity**	96%	92%	92%	97% (as stated in their publication)

Class prediction was performed using support vector machines (SVM). The 100 Agilent transcripts from the Maertzdorf *et al* study translated to 76 recognised genes using the DAVID gene converter. The SVM model was built in the Training Set and run in the Test and Validation Sets.

**Table 5:** Class prediction of 50 genes from the Koth et al study.

	Present study Training SetTB vs non-TB (controls, sarcoid, cancer, pneumonia)	Present study Test SetTB vs non-TB (controls, sarcoid, cancer, pneumonia)	Present study Validation Set TB vs non-TB(controls & sarcoid)	Maertzdorf et alTB vs non-TB(controls & sarcoid)
**Sensitivity**	75%	45%	50%	Not shown in their publication
**Specificity**	92%	87%	92%	Not shown in their publication

Class prediction was performed using support vector machines (SVM). The 50 genes from the Koth *et al* study were used to build the last SVM model in the Training Set and run in the Test and Validation Sets.

## Discussion

We show an IFN-inducible blood transcriptional signature in patients with the pulmonary granulomatous diseases, TB and sarcoidosis, which is distinct from other lung diseases representing acute and chronic conditions, pneumonia and lung cancer, which were dominated by an inflammatory signature. Sarcoidosis transcriptional profiles revealed heterogeneity correlating with clinical disease activity. The IFN-inducible transcripts appeared to be dominant in the neutrophils in sarcoidosis as well as TB. Treated-TB and pneumonia patients showed significant diminishment of their transcriptional activity, however, the treatment-responsive sarcoidosis patients revealed a significantly more active transcriptional profile dominated by inflammatory transcripts.

We previously reported an IFN-inducible blood transcriptional signature in patients with pulmonary TB, which correlated with extent of radiographic disease, diminished upon treatment [4], [10], and now confirmed in other studies [5], [6], [7]. Similar blood transcriptional profiles dominated by IFN-signalling in TB and sarcoidosis patients, using publicly available data (7) and smaller patient cohorts [8], [9], have since been reported. We now demonstrate an IFN-inducible blood transcriptional signature in TB and sarcoidosis patients using larger cohorts of independently recruited participants and new findings of a distinct signature from pneumonia and lung cancer. The heterogeneity of sarcoidosis blood transcriptional profiles was explained by a significant correlation with the clinical activity phenotype, not reported previously [8], [9], [16], but in accordance with the known clinical heterogeneity of sarcoidosis patients [11], [17]. Lockstone *et al.,* demonstrated a correlation between expression profiles of lung biopsies from sarcoidosis patients, and their clinical classification as progressive-fibrotic or self-limited after follow-up [18]. Collectively these findings suggest the potential for blood transcriptomic approaches to provide information before prolonged follow-up of a patient and allow development of much needed tools to standardise and aid sarcoidosis management.

In contrast to the IFN-signalling pathways in TB and sarcoidosis, the transcriptomes in pneumonia and lung cancer represented inflammation related pathways, in keeping with different immunopathogenesis of these diseases, and pneumonia as an infection of the respiratory tract resulting in acute inflammation of the lungs and peripheral blood [19], [20]. The inflammatory blood transcriptional signature of lung cancer patients is in keeping with a role for inflammation in primary lung cancer [21], suggesting blood could be used for further exploratory studies where tissue is not available. The under-abundance of transcripts relating to T and B cells in all four diseases is consistent with previous observations of reduced numbers of immune cells in the blood of patients with TB, sarcoidosis and bacterial infection, likely due to migration or death [4], [22], [23].

Although TB and sarcoidosis exhibited similar IFN-inducible transcriptional signatures, the significant quantitative difference in their transcriptional activity in whole blood and the neutrophils likely reflects the observed clinical differences. Sarcoidosis patients with a dominant IFN-inducible signature clustered with the TB patients whereas those with a much weaker IFN-inducible profile clustered with the healthy controls. Although IFNγ has been shown to be crucial in controlling mycobacterial infection [24], type 1 IFN, induced by extracellular mycobacterial DNA activation of cytosolic receptors [25], or peptidoglycan activation of the NOD2/IRF5 pathway by [26], may exacerbate TB [27] [4], [10], [28]. The immune pathways contributing to sarcoidosis remain unresolved although suggested to be associated with macrophage and Th1 activation [29]. It is notable that IFNα therapy for hepatitis C is a documented risk factor for developing sarcoidosis (approximately 5% of cases) [30]; and IFNß therapy has also been associated with case reports of sarcoidosis [31]. That sarcoidosis and TB patients show a similar transcriptional signature suggests that the underlying immunological processes of these two granulomatous diseases have much in common [29]. A frequently suggested aetiological agent for sarcoidosis is mycobacteria, however the evidence for this is debated [32]. Establishing whether this IFN-inducible signature is reflective of granulomatous inflammation, or a response to pathogens such as mycobacteria, requires studies of additional pulmonary granulomatous diseases.

Successful treatment of TB and pneumonia patients with anti-microbial drugs led to diminishment of the blood transcriptional signature. However, sarcoidosis patients responding successfully to treatment showed a significant increase in transcriptional activity by MDTH of inflammatory transcripts, including the anti-inflammatory genes, IL1R2, IL18RAP, DUSP1, FOS, IκBα and MAPK1, which invariably accompany the activation of inflammatory pathways [33], [34], [35]. This is consistent with the mixed transcriptional response found after glucocorticoid stimulation of blood from healthy donors [36]. However, the IFN-inducible signature was unchanged. In SLE it has been shown that while glucocorticoids suppress the inflammatory NF-κB pathway in many cells, they exerted no effect on secretion of IFNα by plasmacytoid dendritic cells, providing a potential reason for the reduced glucocorticoid sensitivity seen in SLE [14]. The underlying mechanisms resulting in the partial or negligible clinical responses towards glucocorticoids seen in many sarcoidosis patients are as yet undetermined [37].

We identified 144 differentially expressed transcripts between TB and active sarcoidosis, including IFN-inducible transcripts that were over-abundant only in the TB patients and distinguished the TB samples from all other diseases in our three cohorts and an external cohort (Maertzdorf *et al* cohort) [9], (sensitivity >80%; specificity >90%). The two previously published studies comparing whole blood expression profiles of TB and sarcoidosis patients also derived transcript lists to differentiate the diseases [8], [9], however their lists produced much lower sensitivity, but similar specificity values when tested against our cohorts. The high specificity achieved by all transcript lists was due to the very low Type I error rate attributable to the low prevalence of true TB samples. Thus blood transcriptional signatures may have promise as supportive surrogate markers for pulmonary TB diagnosis, after satisfying rigorous testing and validation in large populations.

We have shown that the blood transcriptome of sarcoidosis like TB is dominated by an IFN-inducible neutrophil-driven signature, and heterogeneity of this signature is reflective of disease activity in sarcoidosis. In contrast, pneumonia and lung cancer were dominated by an inflammatory signature. Identification of biological pathways by transcriptomics enhances our understanding of the potential factors underlying pathogens is in the pulmonary granulomatous diseases TB and sarcoidosis and the acute and chronic pulmonary diseases, pneumonias and lung cancers.

## Supporting Information

Figure S1 **Recruitment flow diagrams for each disease group and healthy controls in the Training Set.**
(PDF)

Figure S2 **Recruitment flow diagrams for each disease group and healthy controls in the Test Set.**
(PDF)

Figure S3 **Recruitment flow diagrams for each disease group and healthy controls in the Validation Set.**
(PDF)

Figure S4 **Pulmonary granulomatous diseases display similar transcriptional signatures that are distinct from pneumonia and lung cancer.** (A) 3422-transcripts derived by unsupervised analysis in the Training Set, prior to the application of a statistical filter, in the whole blood of healthy controls, pulmonary TB patients, pulmonary sarcoidosis patients, pneumonia patients and lung cancer patients. The 3422 transcripts and patients’ profiles are organised by unsupervised hierarchical clustering. (B) After adding a statistical filter to the 3422-transcripts, 1446-transcripts were derived as differentially expressed across all the groups in the Training Set. The clustering of the 1446-transcripts are tested here in an independent cohort, the Test Set. A dotted line is added to the heatmaps to clarify the main clusters generated by the clustering algorithm. Transcript intensity values are normalised to the median of all transcripts. Red transcripts are relatively over-abundant and blue transcripts under-abundant. The coloured bar at the bottom of the heatmap indicates which group the profile belongs to.
(PDF)

Figure S5 **Clinical decision tree for classifying sarcoidosis patients.** The decision tree demonstrates how each sarcoidosis patient was classified into active pulmonary, active extra-thoracic or non-active sarcoidosis using clinical variables known to be associated with disease activity and routinely measured as part of standard medical care.
(PDF)

Figure S6 **Active sarcoidosis signatures are similar to TB but distinct from non-sarcoidosis which resembles healthy controls.** 1396-transcripts are differentially expressed in the whole blood of healthy controls, pulmonary TB patients, active sarcoidosis patients, non-active sarcoidosis patients, pneumonia patients and lung cancer patients. The 1396 transcripts and patients’ profiles are organised by unsupervised hierarchical clustering. A dotted line is added to the heatmap to clarify the main clusters generated by the clustering algorithm. Transcript intensity values are normalised to the median of all transcripts. Red transcripts are relatively over-abundant and blue transcripts under-abundant. The coloured bar at the bottom of the heatmap indicates which group the profile belongs to. (A) Test Set (B) Validation Set.
(PDF)

Figure S7 **Modular analysis shows similar pathways associated with TB and sarcoidosis, differing from pneumonia and cancer.** Gene expression levels of all transcripts that were significantly detected compared to background hybridisation (18894 transcripts, *p*<0.01) were compared between each patient group: TB, active sarcoidosis, non-active sarcoidosis, pneumonia, lung cancer, to the healthy controls in the Test Set. Each module corresponds to a set of co-regulated genes that were assigned biological functions by unbiased literature profiling. A red dot indicates significant over-abundance of transcripts and a blue dot indicates significant under-abundance (p<0.05). The colour intensity correlates to the percentage of genes in that module that are significantly differentially expressed.
(PDF)

Figure S8 **Neutrophil module.** (A) The mean percentage of genes significantly overexpressed in the neutrophil module for each disease in both the Training and Test set. (B) The mean fold change of the expression of the genes present in the neutrophil module compared to the controls. The mean, SEM and *p-*values are displayed (ANOVA with Tukey’s multiple comparison test).
(PDF)

Figure S9 **Venn diagram comparing differentially expressed genes for each disease group compared to their matched controls.** Differentially expressed genes were derived from the Training Set by comparing each disease to healthy controls matched for ethnicity and gender: TB = 2524, active sarcoidosis = 1391, pneumonia = 2801 and lung cancer = 1626 transcripts (≥1.5 fold change from the mean of the controls, Mann Whitney Benjamini Hochberg *p*<0.01). The 4-set Venn diagram was created using Venny (Oliveros 2007). IPA canonical pathways was used to determined the most significant pathways associated with the unique transcripts for each disease (Fisher’s exact FDR = 0.05). Active Sarc = active sarcoidosis.
(PDF)

Figure S10 **Venn diagram comparing the gene lists used in the class prediction.** The gene lists were obtained from this study (144 Illumina probes), Maertzdorf et al study (100 Agilent probes of which only 76 probes were recognised as genes using DAVID converter) and Koth et al study (50 genes obtained from a Affymetrix platform). In the Illumina platform used to compare these lists some genes are represented by more than one transcript for example the 50 genes in Koth et al study translate to 77 Illumina probes/transcripts.
(PDF)

Figure S11 **Receiver operating curves of the gene lists used in the class prediction.** Receiver operating curves and area under the curves calculations are shown in parallel to the support vector machine results in tables 3–5. (A) 144 transcripts from our study (B) 76 probes from Maertzdorf *et al* study (C) 50 genes from Koth *et al* study.
(PDF)

Table S1 **Demographics of the patients and controls recruited.** (A) Training Set and Test Set total numbers, age, gender and ethnicity. (B) Validation Set.
(PPTX)

Table S2 **Clinical characteristics of the Training set are not significantly different to the Test and Validation Sets.** (A–D) Clinical characteristics of the patients in the Training Set. (E-H) Comparing the clinical characteristics of the patients in the Training Set to those of the patients in the Test and Validation Sets (t-test or Chi-squared *p*<0.05). BAL = bronchoalveolar lavage, IGRA = IFN gamma-release assay, Lymph = lymphocyte count, BHL = bilateral hilar lymphadenopathy, Neut = neutrophil count, CXR = chest X-ray, ISC = Indian subcontinent, CRP = C-reactive protein, Ind = indeterminate, ND = not done, N/A = not available, pred = prednisolone. Dyspnoea = breathlessness. Haemoptysis = coughing up blood. CURB65 score = pneumonia severity score where 5 is the most severe. HT = hypertension. DM = hypertension. Adeno = adenocarcinoma.
(PPTX)

Table S3 **Clinical characteristics and clinical classification of sarcoidosis patients as determine by the decision tree.** (A) Training Set (B) Test Set (C) Validation Set (D) Demographics of all sarcoidosis patients in the three datasets. CXR = chest radiograph, CT = computer tomography, ACE = angiotensin converting enzyme, Lymph = lymphocyte count, Neut = neutrophil count, TLCO = transfer factor for carbon monoxide, KCO = transfer coefficient, FVC = forced vital capacity, FEV1 = forced expiratory volume in 1 second, Abdo = abdomen, LN = lymph node, Med = mediastinal, NA = non-active sarcoidosis, AET = active extra-thoracic sarcoidosis, Neuro = neurological disease.
(PPTX)

Table S4 **The clinical classification decision tree used to categorise patients into either active or non-active sarcoidosis predicts the clustering of the transcriptional profiles of the sarcoidosis patients better than standard single or multiple clinical variables.** (A) Univariate regression analysis to determine which single clinical variables can best predict those sarcoidosis patients that will cluster with the TB patients and those that will cluster with the healthy controls as per the unsupervised clustering of the 1446-transcripts in the Training set and Test set (see Figures 1 &S4b) (B) Multivariate analysis to determine the ability of more than one variable to predict the clustering of the sarcoidosis patients.
(PPTX)

Table S5 **The top 50 differentially expressed transcripts for each disease compared to matched controls.** Differentially expressed genes were derived from the Training Set by comparing each disease to healthy controls matched for ethnicity and gender: TB = 2524, active sarcoidosis = 1391, pneumonia = 2801 and lung cancer = 1626 transcripts (≥1.5 fold change from the mean of the controls, Mann Whitney Benjamini Hochberg *p*<0.01).
(PPTX)

Table S6 **The top 50 differentially expressed transcripts unique for each disease as determined by the 4-set Venn diagram.** Differentially expressed genes were derived from the Training Set by comparing each disease to healthy controls matched for ethnicity and gender (≥1.5 fold change from the mean of the controls, Mann Whitney Benjamini Hochberg *p*<0.01). A 4-set Venn diagram was used to identify genes that were unique for each disease.
(PPTX)

Table S7 **Drug therapy given to each sarcoidosis patient who was commenced on treatment and the clinical management of their practising physician after observing their response to the therapy.** Each patients study ID and ‘treatment response category’ correlates with the legend used for the modular analysis. The superscript number of the sarcoidosis patients is used when the patient had more than one visit to their practising physician such that 2^2^ was the visit after being started on treatment and 2^3^ was the subsequent visit.
(PPTX)

Table S8 **Top 50 over-expressed genes in the inflammation modules in the good-treatment response sarcoidosis patients.**
(PPTX)

Table S9 **Interferon-inducible genes from the Berry** ***et al*** **2010 publication.**
(PPTX)

Table S10 **Demographics of study participants used in the cell purification.**
(PPTX)

Table S11 **List of transcripts present in the IPA canonical pathways shown in** **Figure 5** **.** List of all transcripts by gene symbol that are present in the IPA canonical pathways: EIF2 signalling, interferon signalling, role of pattern recognition receptors in recognition of bacteria and viruses, and antigen presentation pathway.
(PPTX)
