# Correction: Transcriptional Blood Signatures Distinguish Pulmonary Tuberculosis, Pulmonary Sarcoidosis, Pneumonias and Lung Cancers

**DOI:** 10.1371/annotation/7d9ec449-aee0-48fe-8111-0c110850c0c1

**Published:** 2013-08-21

**Authors:** Chloe I. Bloom, Christine M. Graham, Matthew P. R. Berry, Fotini Rozakeas, Paul S. Redford, Yuanyuan Wang, Zhaohui Xu, Katalin A. Wilkinson, Robert J. Wilkinson, Yvonne Kendrick, Gilles Devouassoux, Tristan Ferry, Makoto Miyara, Diane Bouvry, Valeyre Dominique, Guy Gorochov, Derek Blankenship, Mitra Saadatian, Phillip Vanhems, Huw Beynon, Rama Vancheeswaran, Melissa Wickremasinghe, Damien Chaussabel, Jacques Banchereau, Virginia Pascual, Ling-pei Ho, Marc Lipman, Anne O’Garra

The name of the fifteenth author was incorrect.

The correct name is: Dominique Valeyre. 

