# Supplementary material for: Transcriptional Blood Signatures Distinguish Pulmonary Tuberculosis, Pulmonary Sarcoidosis, Pneumonias and Lung Cancers
Source: PLoS One. 2013 Aug 5;8(8):e70630. doi: 10.1371/journal.pone.0070630 (PMC3734176; doi:10.1371/journal.pone.0070630)
Supplement: Figure S1 — Recruitment flow diagrams for each disease group and healthy controls in the Training Set. (PDF) [file pone.0070630.s001.pdf]

Figure S1

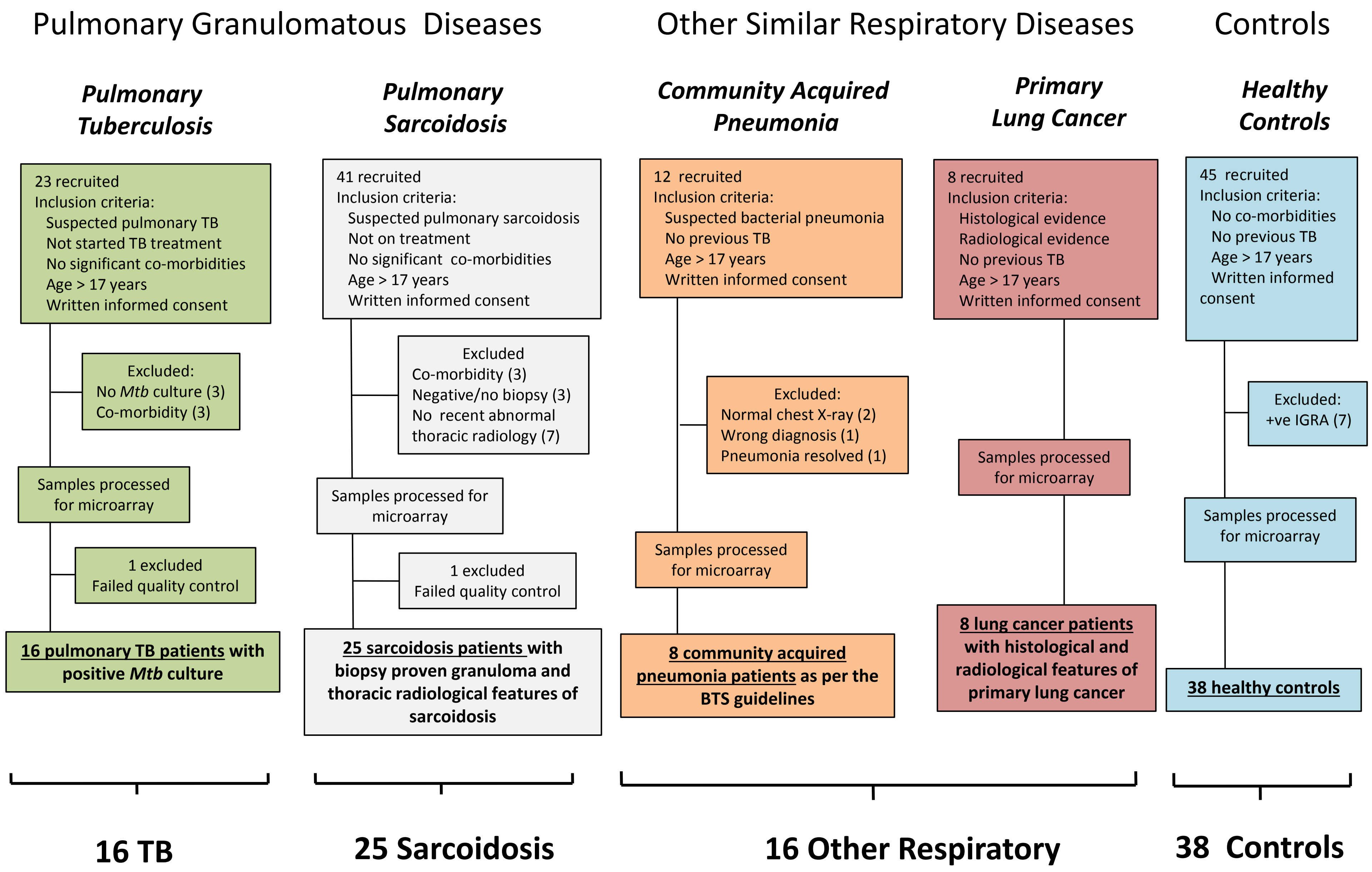
Pulmonary Granulomatous Diseases
Other Similar Respiratory Diseases
Controls
Pulmonary Tuberculosis
Pulmonary Sarcoidosis
Community Acquired Pneumonia
Primary Lung Cancer
Healthy Controls
23 recruited
Inclusion criteria:
Suspected pulmonary TB
Not started TB treatment
No significant co-morbidities
Age > 17 years
Written informed consent
Excluded:
No Mtb culture (3)
Co-morbidity (3)
Samples processed for microarray
1 excluded
Failed quality control
16 pulmonary TB patients with positive Mtb culture
41 recruited
Inclusion criteria:
Suspected pulmonary sarcoidosis
Not on treatment
No significant co-morbidities
Age > 17 years
Written informed consent
Excluded
Co-morbidity (3)
Negative/no biopsy (3)
No recent abnormal thoracic radiology (7)
Samples processed for microarray
1 excluded
Failed quality control
25 sarcoidosis patients with biopsy proven granuloma and thoracic radiological features of sarcoidosis
12 recruited
Inclusion criteria:
Suspected bacterial pneumonia
No previous TB
Age > 17 years
Written informed consent
Excluded:
Normal chest X-ray (2)
Wrong diagnosis (1)
Pneumonia resolved (1)
Samples processed for microarray
8 community acquired pneumonia patients as per the BTS guidelines
8 recruited
Inclusion criteria:
Histological evidence
Radiological evidence
No previous TB
Age > 17 years
Written informed consent
Samples processed for microarray
8 lung cancer patients with histological and radiological features of primary lung cancer
45 recruited
Inclusion criteria:
No co-morbidities
No previous TB
Age > 17 years
Written informed consent
Excluded:
+ve IGRA (7)
Samples processed for microarray
38 healthy controls
16 TB
25 Sarcoidosis
16 Other Respiratory
38 Controls
