# Supplementary material for: Transcriptional Blood Signatures Distinguish Pulmonary Tuberculosis, Pulmonary Sarcoidosis, Pneumonias and Lung Cancers
Source: PLoS One. 2013 Aug 5;8(8):e70630. doi: 10.1371/journal.pone.0070630 (PMC3734176; doi:10.1371/journal.pone.0070630)
Supplement: Figure S2 — Recruitment flow diagrams for each disease group and healthy controls in the Test Set. (PDF) [file pone.0070630.s002.pdf]

Figure S2

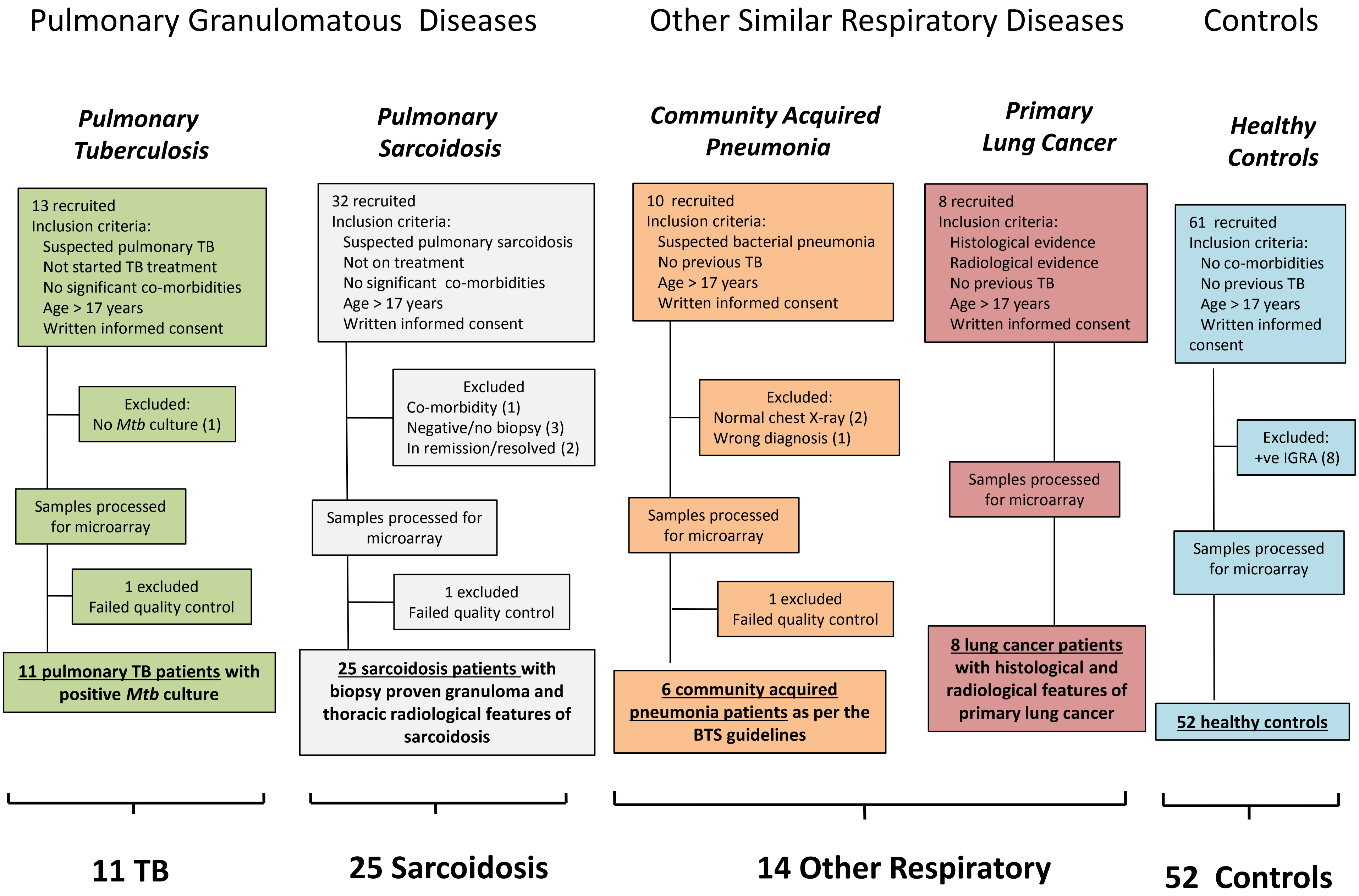
Pulmonary Granulomatous Diseases
Other Similar Respiratory Diseases
Controls
Pulmonary Tuberculosis
13 recruited
Inclusion criteria:
Suspected pulmonary TB
Not started TB treatment
No significant co-morbidities
Age > 17 years
Written informed consent
Excluded:
No Mtb culture (1)
Samples processed for microarray
1 excluded
Failed quality control
11 pulmonary TB patients with positive Mtb culture
Pulmonary Sarcoidosis
32 recruited
Inclusion criteria:
Suspected pulmonary sarcoidosis
Not on treatment
No significant co-morbidities
Age > 17 years
Written informed consent
Excluded
Co-morbidity (1)
Negative/no biopsy (3)
In remission/resolved (2)
Samples processed for microarray
1 excluded
Failed quality control
25 sarcoidosis patients with biopsy proven granuloma and thoracic radiological features of sarcoidosis
Community Acquired Pneumonia
10 recruited
Inclusion criteria:
Suspected bacterial pneumonia
No previous TB
Age > 17 years
Written informed consent
Excluded:
Normal chest X-ray (2)
Wrong diagnosis (1)
Samples processed for microarray
1 excluded
Failed quality control
6 community acquired pneumonia patients as per the BTS guidelines
Primary Lung Cancer
8 recruited
Inclusion criteria:
Histological evidence
Radiological evidence
No previous TB
Age > 17 years
Written informed consent
Samples processed for microarray
8 lung cancer patients with histological and radiological features of primary lung cancer
Healthy Controls
61 recruited
Inclusion criteria:
No co-morbidities
No previous TB
Age > 17 years
Written informed consent
Excluded:
+ve IGRA (8)
Samples processed for microarray
52 healthy controls
11 TB
25 Sarcoidosis
14 Other Respiratory
52 Controls
