# Supplementary material for: Transcriptional Blood Signatures Distinguish Pulmonary Tuberculosis, Pulmonary Sarcoidosis, Pneumonias and Lung Cancers
Source: PLoS One. 2013 Aug 5;8(8):e70630. doi: 10.1371/journal.pone.0070630 (PMC3734176; doi:10.1371/journal.pone.0070630)
Supplement: Figure S3 — Recruitment flow diagrams for each disease group and healthy controls in the Validation Set. (PDF) [file pone.0070630.s003.pdf]

Figure S3

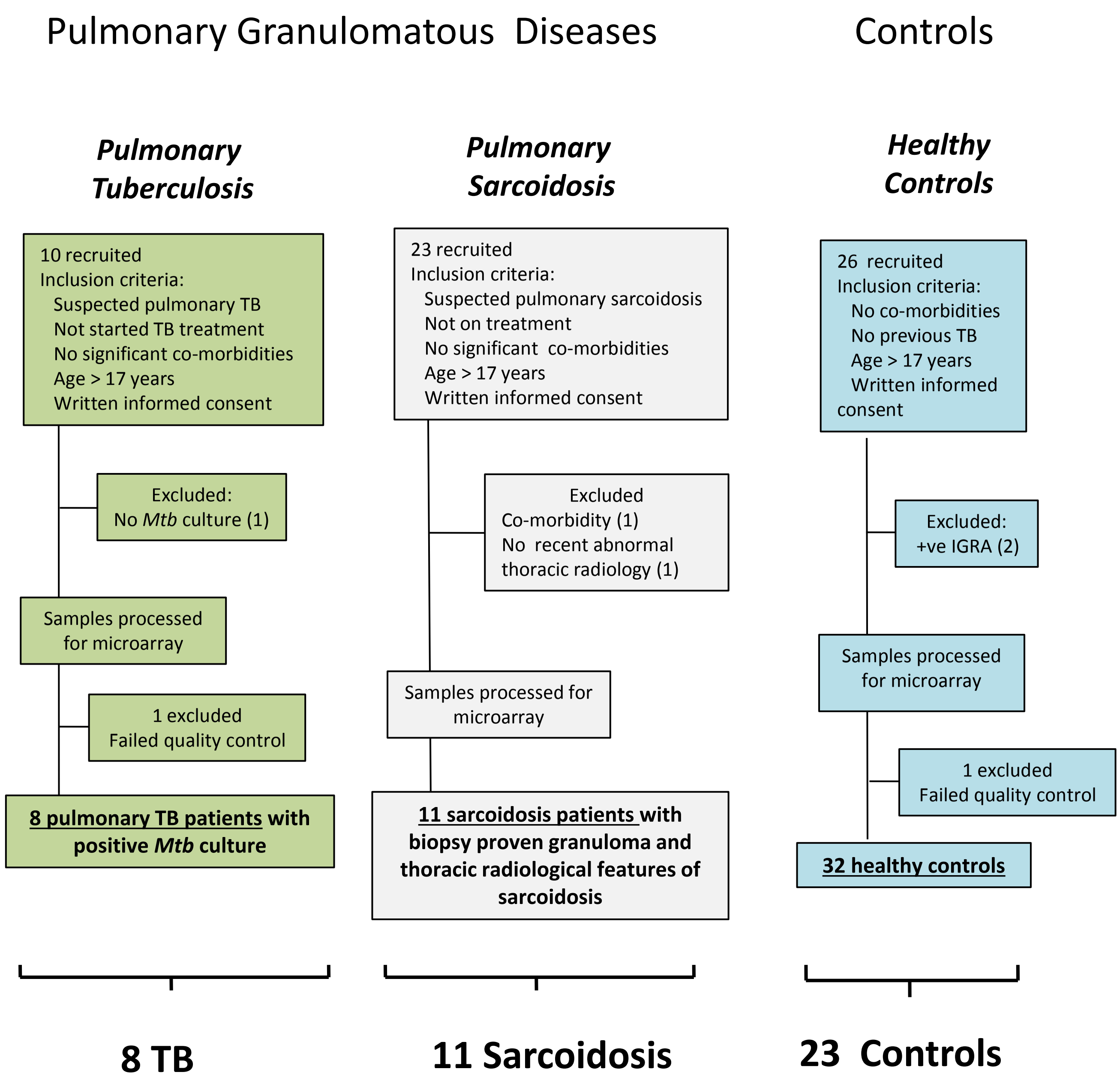
Pulmonary Granulomatous Diseases
Controls
Pulmonary Tuberculosis
Pulmonary Sarcoidosis
Healthy Controls
10 recruited
Inclusion criteria:
Suspected pulmonary TB
Not started TB treatment
No significant co-morbidities
Age > 17 years
Written informed consent
Excluded:
No Mtb culture (1)
Samples processed for microarray
1 excluded
Failed quality control
8 pulmonary TB patients with positive Mtb culture
23 recruited
Inclusion criteria:
Suspected pulmonary sarcoidosis
Not on treatment
No significant co-morbidities
Age > 17 years
Written informed consent
Excluded
Co-morbidity (1)
No recent abnormal thoracic radiology (1)
Samples processed for microarray
11 sarcoidosis patients with biopsy proven granuloma and thoracic radiological features of sarcoidosis
26 recruited
Inclusion criteria:
No co-morbidities
No previous TB
Age > 17 years
Written informed consent
Excluded:
+ve IGRA (2)
Samples processed for microarray
1 excluded
Failed quality control
32 healthy controls
8 TB
11 Sarcoidosis
23 Controls
