# Supplementary figures and images for: Transcriptional Blood Signatures Distinguish Pulmonary Tuberculosis, Pulmonary Sarcoidosis, Pneumonias and Lung Cancers
Source: PLoS One. 2013 Aug 5;8(8):e70630. doi: 10.1371/journal.pone.0070630 (PMC3734176; doi:10.1371/journal.pone.0070630)

**Figure S4A**

*Training Set: 3422 transcripts*

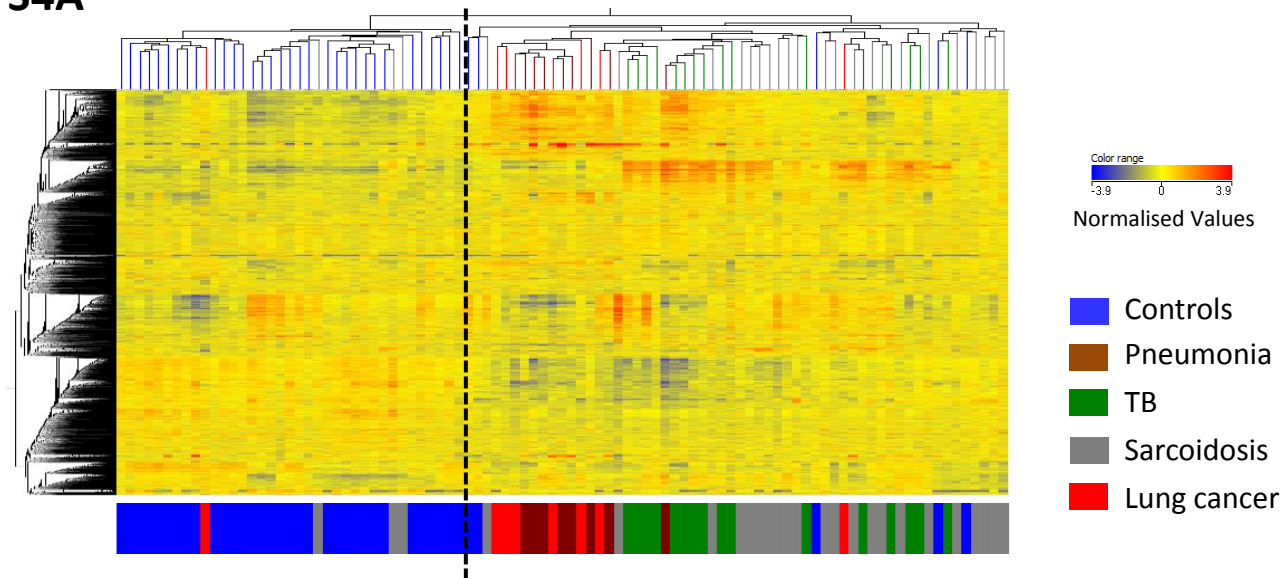

**Figure S4B**

*Training Set: 1446 transcripts*

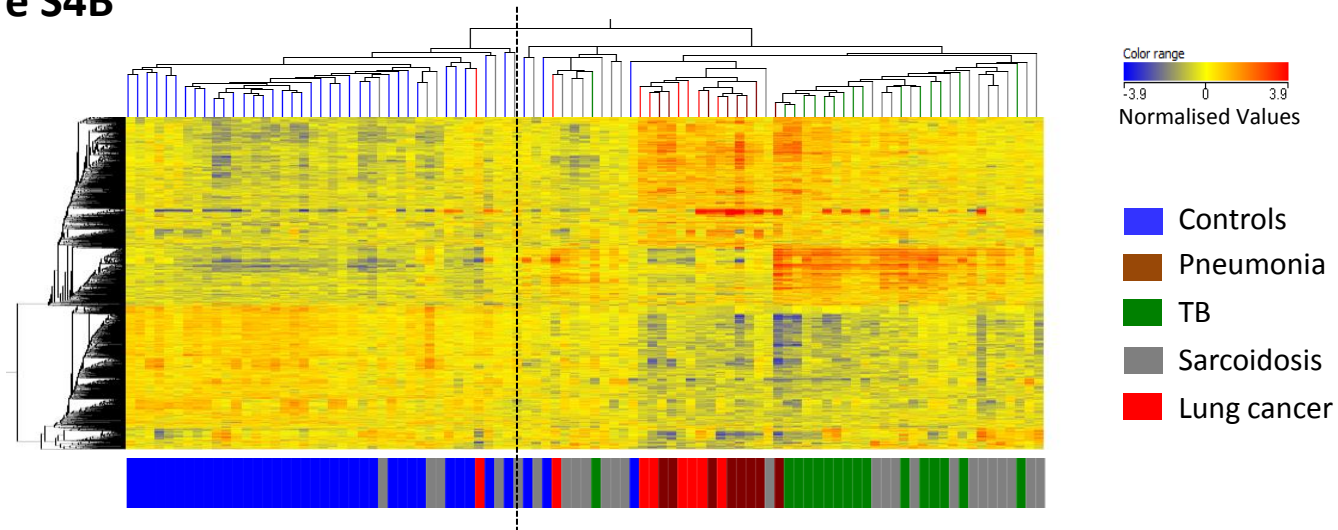

Supplement: Figure S4 — Pulmonary granulomatous diseases display similar transcriptional signatures that are distinct from pneumonia and lung cancer. (A) 3422-transcripts derived by unsupervised analysis in the Training Set, prior to the application of a statistical filter, in the whole blood of healthy controls, pulmonary TB patients, pulmonary sarcoidosis patients, pneumonia patients and lung cancer patients. The 3422 transcripts and patients’ profiles are organised by unsupervised hierarchical clustering. (B) After adding a statistical filter to the 3422-transcripts, 1446-transcripts were derived as differentially expressed across all the groups in the Training Set. The clustering of the 1446-transcripts are tested here in an independent cohort, the Test Set. A dotted line is added to the heatmaps to clarify the main clusters generated by the clustering algorithm. Transcript intensity values are normalised to the median of all transcripts. Red transcripts are relatively over-abundant and blue transcripts under-abundant. The coloured bar at the bottom of the heatmap indicates which group the profile belongs to. (PDF) [file pone.0070630.s004.pdf]

**Figure S5**

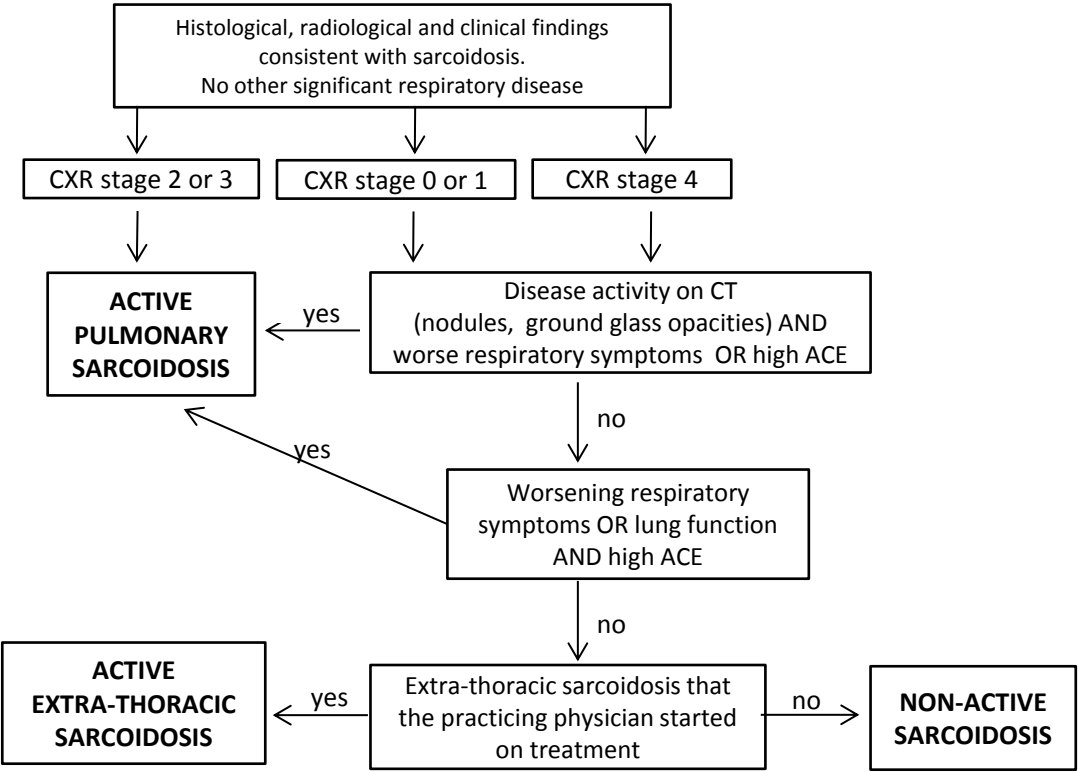

Supplement: Figure S5 — Clinical decision tree for classifying sarcoidosis patients. The decision tree demonstrates how each sarcoidosis patient was classified into active pulmonary, active extra-thoracic or non-active sarcoidosis using clinical variables known to be associated with disease activity and routinely measured as part of standard medical care. (PDF) [file pone.0070630.s005.pdf]

**Figure S6A**

*Test Set: 1396 transcripts*

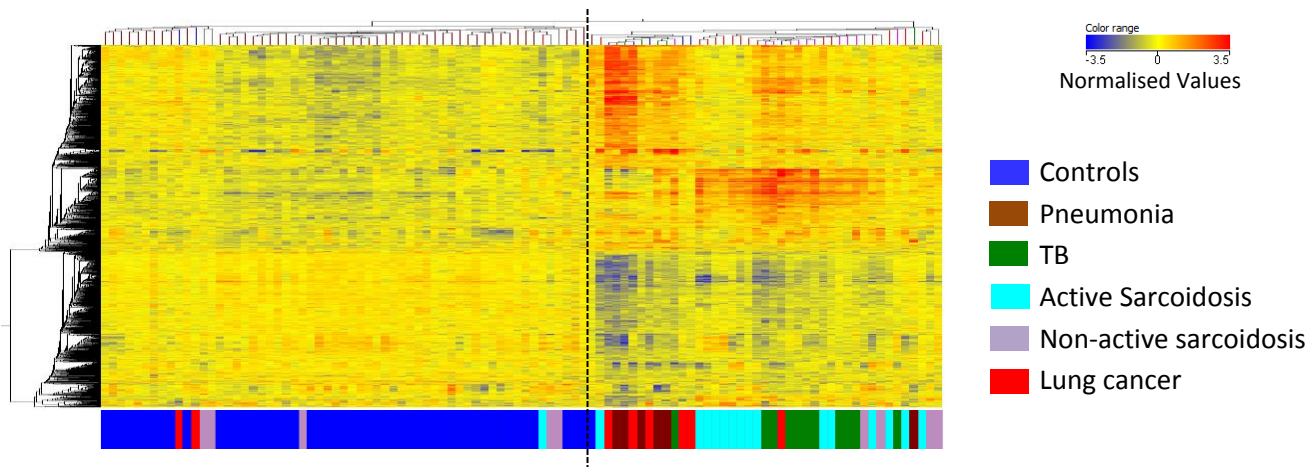

**Figure S6B**

*Validation Set: 1396 transcripts*

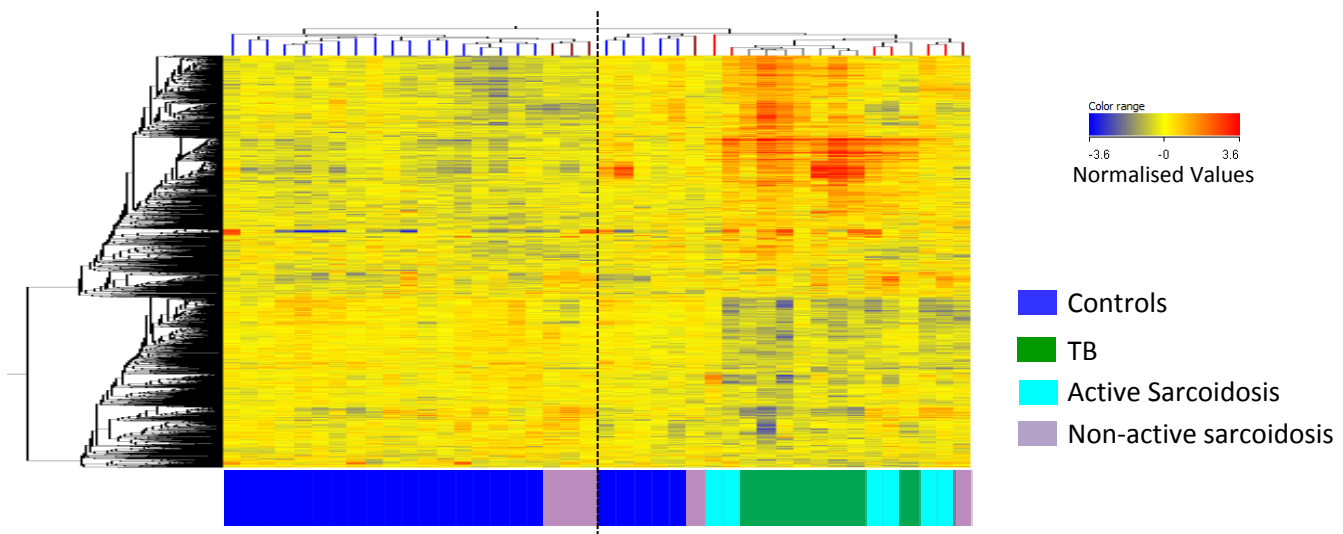

Supplement: Figure S6 — Active sarcoidosis signatures are similar to TB but distinct from non-sarcoidosis which resembles healthy controls. 1396-transcripts are differentially expressed in the whole blood of healthy controls, pulmonary TB patients, active sarcoidosis patients, non-active sarcoidosis patients, pneumonia patients and lung cancer patients. The 1396 transcripts and patients’ profiles are organised by unsupervised hierarchical clustering. A dotted line is added to the heatmap to clarify the main clusters generated by the clustering algorithm. Transcript intensity values are normalised to the median of all transcripts. Red transcripts are relatively over-abundant and blue transcripts under-abundant. The coloured bar at the bottom of the heatmap indicates which group the profile belongs to. (A) Test Set (B) Validation Set. (PDF) [file pone.0070630.s006.pdf]

Figure S7

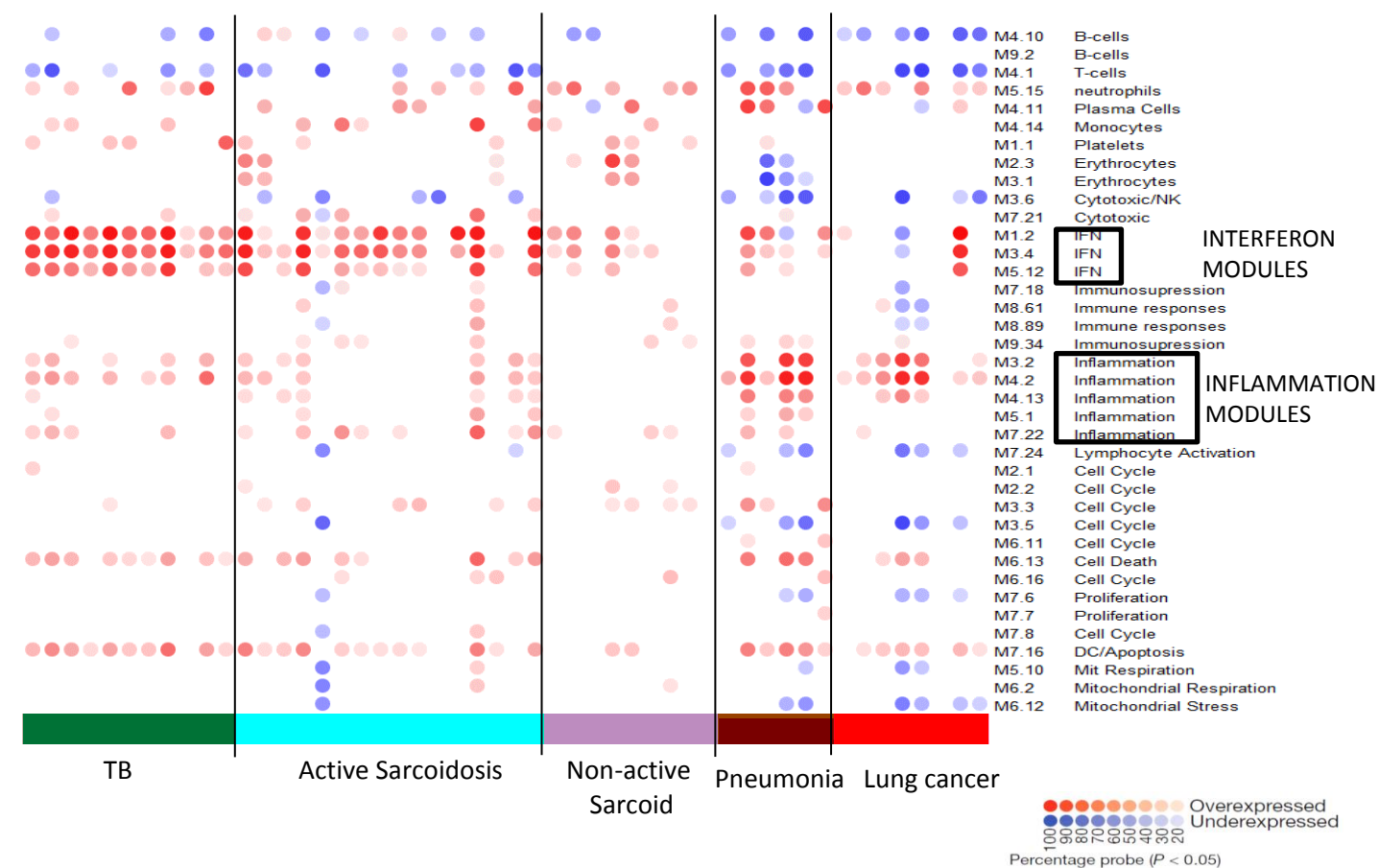

Supplement: Figure S7 — Modular analysis shows similar pathways associated with TB and sarcoidosis, differing from pneumonia and cancer. Gene expression levels of all transcripts that were significantly detected compared to background hybridisation (18894 transcripts, p<0.01) were compared between each patient group: TB, active sarcoidosis, non-active sarcoidosis, pneumonia, lung cancer, to the healthy controls in the Test Set. Each module corresponds to a set of co-regulated genes that were assigned biological functions by unbiased literature profiling. A red dot indicates significant over-abundance of transcripts and a blue dot indicates significant under-abundance (p<0.05). The colour intensity correlates to the percentage of genes in that module that are significantly differentially expressed. (PDF) [file pone.0070630.s007.pdf]

Figure S8A

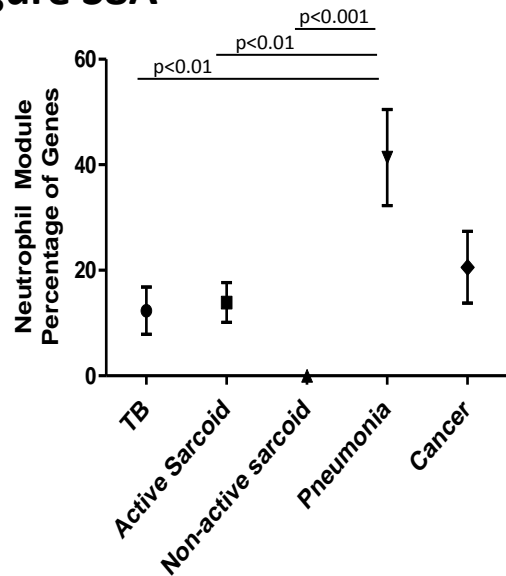

Figure S8B

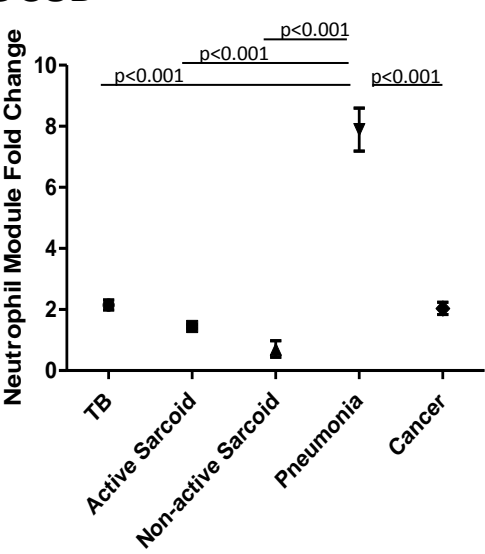

Supplement: Figure S8 — Neutrophil module. (A) The mean percentage of genes significantly overexpressed in the neutrophil module for each disease in both the Training and Test set. (B) The mean fold change of the expression of the genes present in the neutrophil module compared to the controls. The mean, SEM and p-values are displayed (ANOVA with Tukey’s multiple comparison test). (PDF) [file pone.0070630.s008.pdf]

**Figure S9**

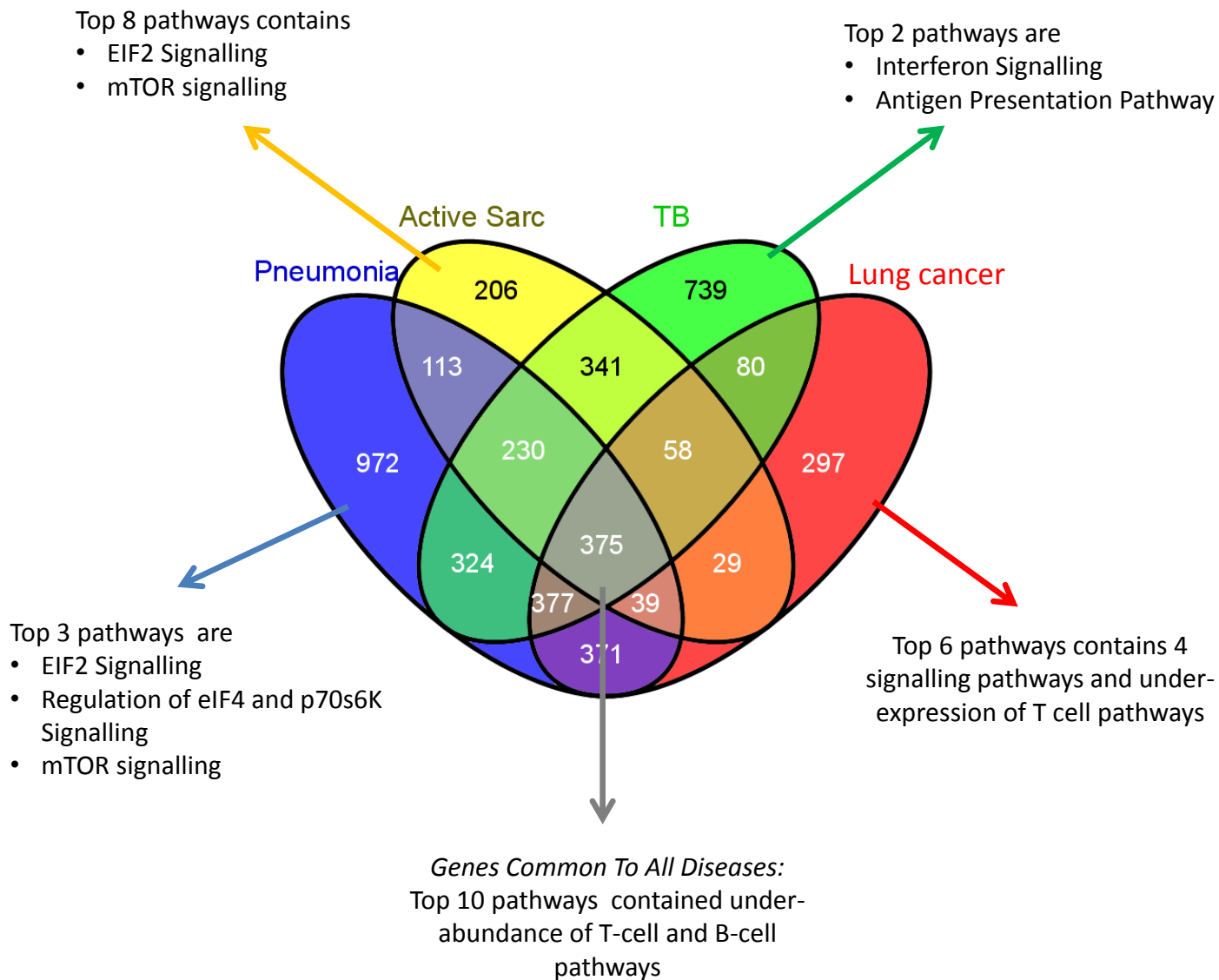

Supplement: Figure S9 — Venn diagram comparing differentially expressed genes for each disease group compared to their matched controls. Differentially expressed genes were derived from the Training Set by comparing each disease to healthy controls matched for ethnicity and gender: TB = 2524, active sarcoidosis = 1391, pneumonia = 2801 and lung cancer = 1626 transcripts (≥1.5 fold change from the mean of the controls, Mann Whitney Benjamini Hochberg p<0.01). The 4-set Venn diagram was created using Venny (Oliveros 2007). IPA canonical pathways was used to determined the most significant pathways associated with the unique transcripts for each disease (Fisher’s exact FDR = 0.05). Active Sarc = active sarcoidosis. (PDF) [file pone.0070630.s009.pdf]

**Figure S10**

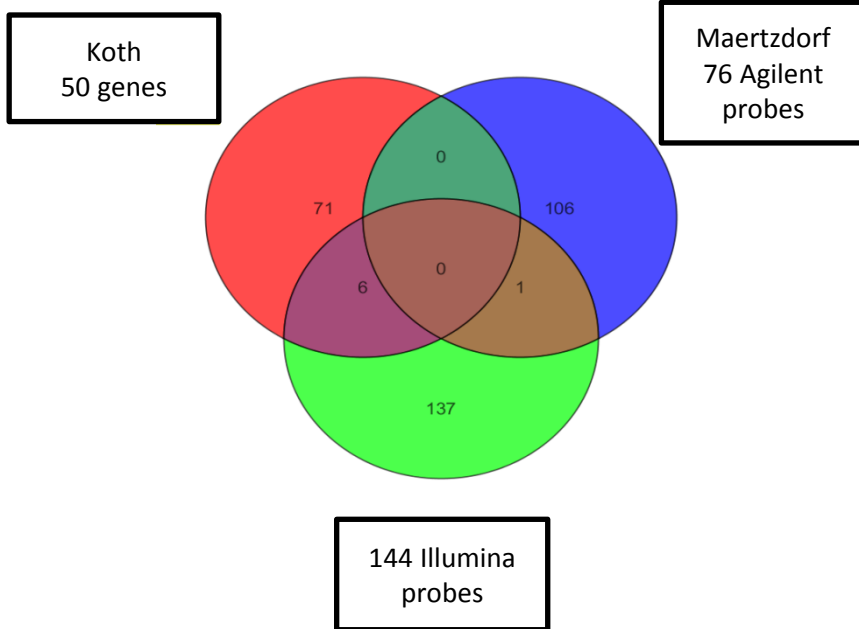

Supplement: Figure S10 — Venn diagram comparing the gene lists used in the class prediction. The gene lists were obtained from this study (144 Illumina probes), Maertzdorf et al study (100 Agilent probes of which only 76 probes were recognised as genes using DAVID converter) and Koth et al study (50 genes obtained from a Affymetrix platform). In the Illumina platform used to compare these lists some genes are represented by more than one transcript for example the 50 genes in Koth et al study translate to 77 Illumina probes/transcripts. (PDF) [file pone.0070630.s010.pdf]

Figure S11

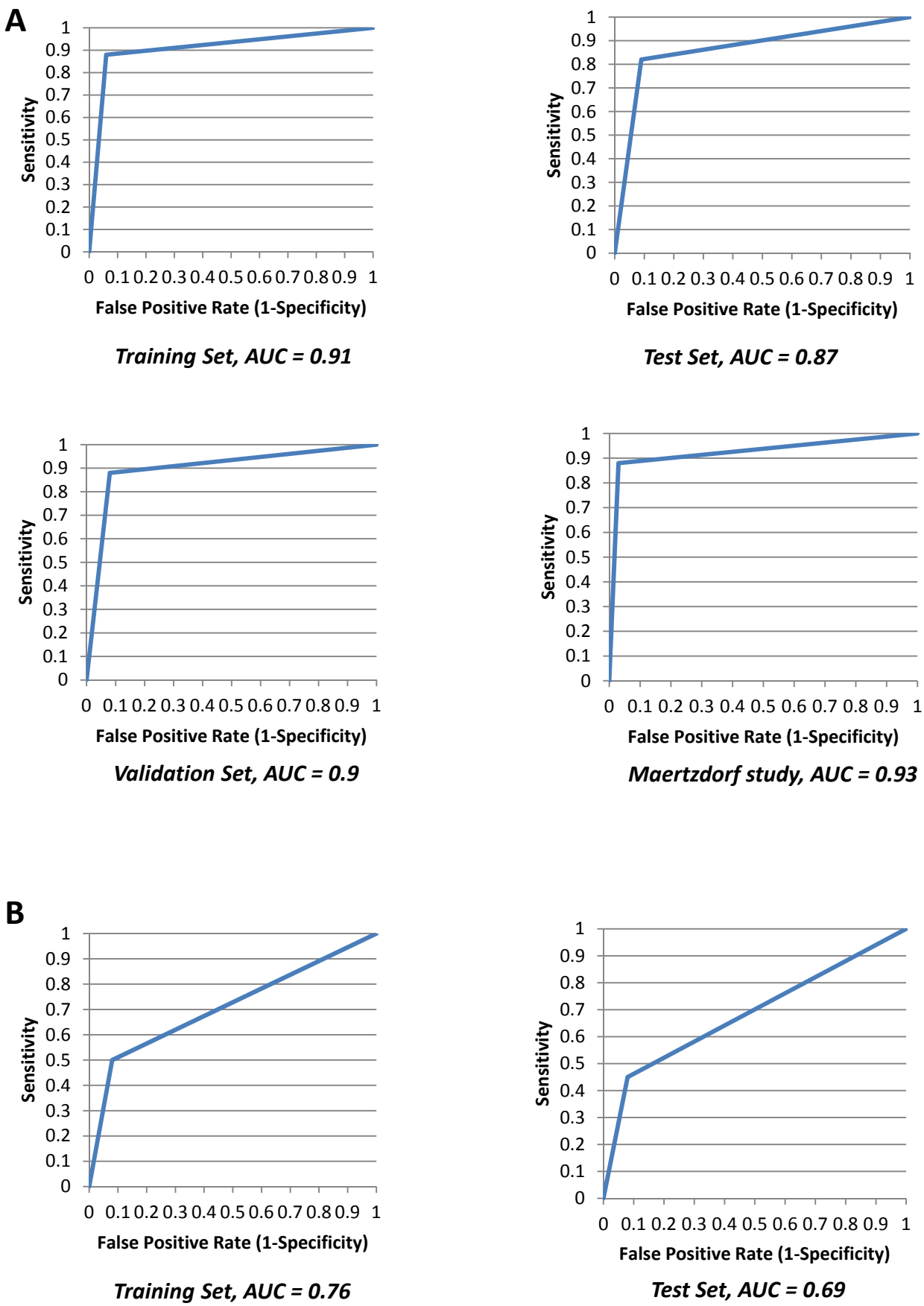

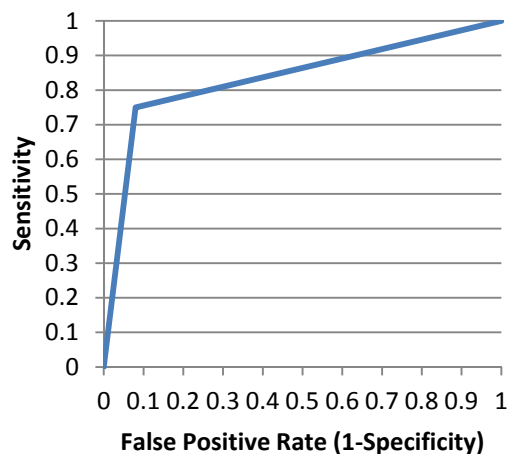

**C**

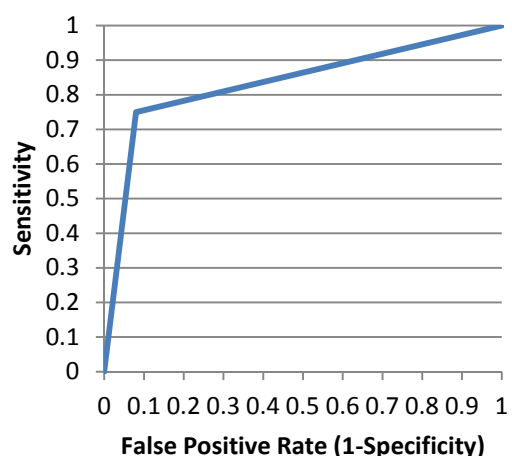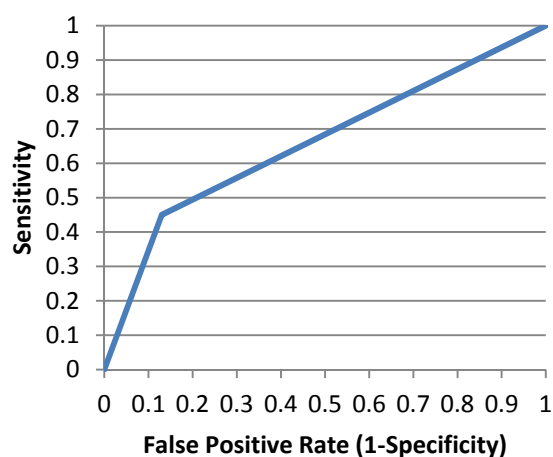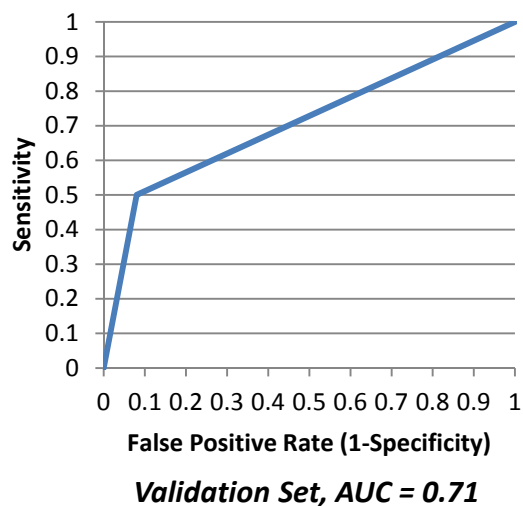

Supplement: Figure S11 — Receiver operating curves of the gene lists used in the class prediction. Receiver operating curves and area under the curves calculations are shown in parallel to the support vector machine results in tables 3–5. (A) 144 transcripts from our study (B) 76 probes from Maertzdorf et al study (C) 50 genes from Koth et al study. (PDF) [file pone.0070630.s011.pdf]
