# Supplementary material for: Transcriptional Blood Signatures Distinguish Pulmonary Tuberculosis, Pulmonary Sarcoidosis, Pneumonias and Lung Cancers
Source: PLoS One. 2013 Aug 5;8(8):e70630. doi: 10.1371/journal.pone.0070630 (PMC3734176; doi:10.1371/journal.pone.0070630)
Supplement: Table S1 — Demographics of the patients and controls recruited. (A) Training Set and Test Set total numbers, age, gender and ethnicity. (B) Validation Set. (PPTX) [file pone.0070630.s012.pptx]

## Slide 1
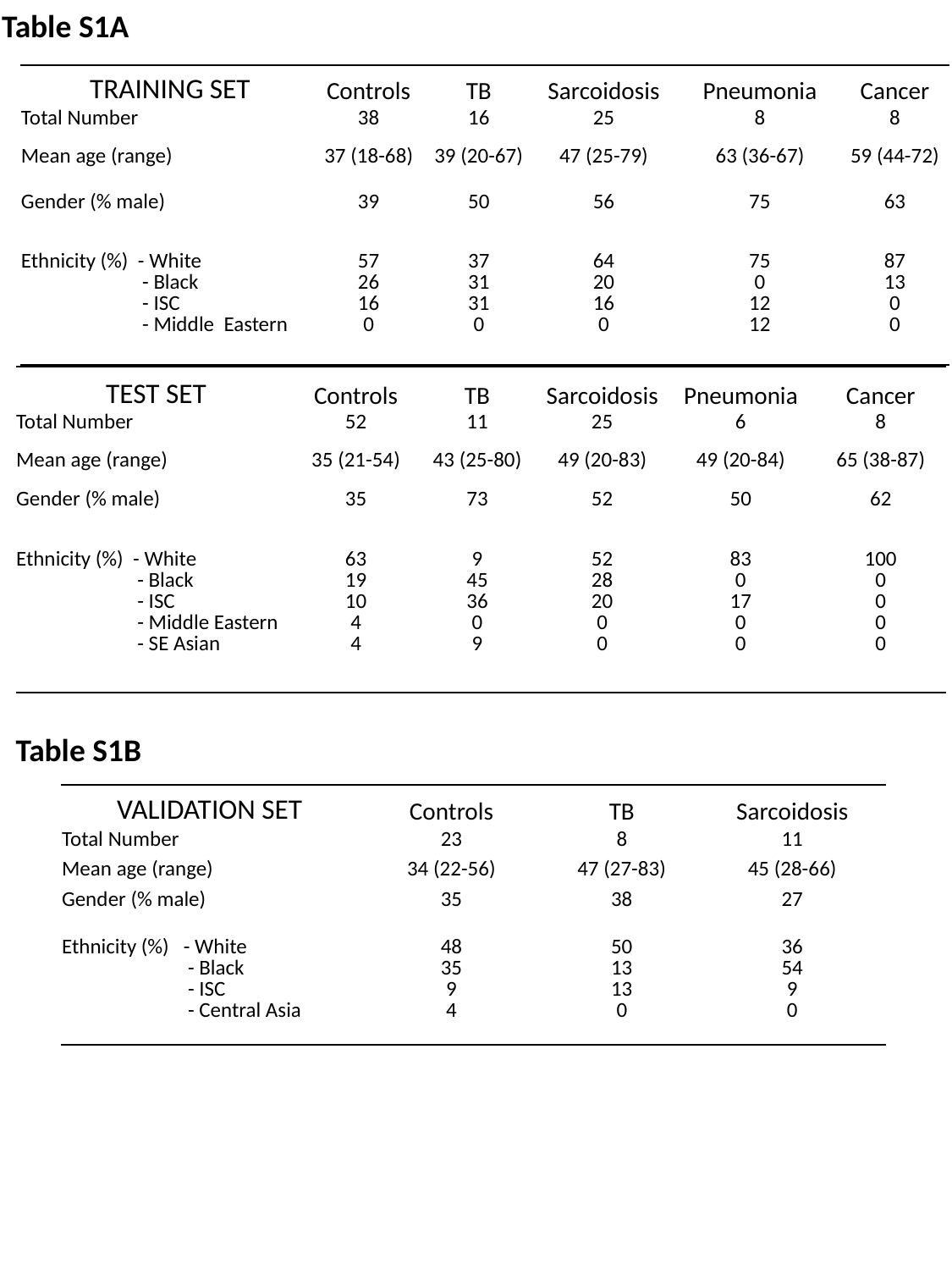

Table S1A
| TRAINING SET | Controls | TB | Sarcoidosis | Pneumonia | Cancer |
| --- | --- | --- | --- | --- | --- |
| Total Number | 38 | 16 | 25 | 8 | 8 |
| Mean age (range) | 37 (18-68) | 39 (20-67) | 47 (25-79) | 63 (36-67) | 59 (44-72) |
| Gender (% male) | 39 | 50 | 56 | 75 | 63 |
| Ethnicity (%) - White - Black - ISC - Middle Eastern | 57 26 16 0 | 37 31 31 0 | 64 20 16 0 | 75 0 12 12 | 87 13 0 0 |
| TEST SET | Controls | TB | Sarcoidosis | Pneumonia | Cancer |
| --- | --- | --- | --- | --- | --- |
| Total Number | 52 | 11 | 25 | 6 | 8 |
| Mean age (range) | 35 (21-54) | 43 (25-80) | 49 (20-83) | 49 (20-84) | 65 (38-87) |
| Gender (% male) | 35 | 73 | 52 | 50 | 62 |
| Ethnicity (%) - White - Black - ISC - Middle Eastern - SE Asian | 63 19 10 4 4 | 9 45 36 0 9 | 52 28 20 0 0 | 83 0 17 0 0 | 100 0 0 0 0 |
Table S1B
| VALIDATION SET | Controls | TB | Sarcoidosis |
| --- | --- | --- | --- |
| Total Number | 23 | 8 | 11 |
| Mean age (range) | 34 (22-56) | 47 (27-83) | 45 (28-66) |
| Gender (% male) | 35 | 38 | 27 |
| Ethnicity (%) - White - Black - ISC - Central Asia | 48 35 9 4 | 50 13 13 0 | 36 54 9 0 |
