# Supplementary material for: Transcriptional Blood Signatures Distinguish Pulmonary Tuberculosis, Pulmonary Sarcoidosis, Pneumonias and Lung Cancers
Source: PLoS One. 2013 Aug 5;8(8):e70630. doi: 10.1371/journal.pone.0070630 (PMC3734176; doi:10.1371/journal.pone.0070630)
Supplement: Table S2 — Clinical characteristics of the Training set are not significantly different to the Test and Validation Sets. (A–D) Clinical characteristics of the patients in the Training Set. (E-H) Comparing the clinical characteristics of the patients in the Training Set to those of the patients in the Test and Validation Sets (t-test or Chi-squared p<0.05). BAL = bronchoalveolar lavage, IGRA = IFN gamma-release assay, Lymph = lymphocyte count, BHL = bilateral hilar lymphadenopathy, Neut = neutrophil count, CXR = chest X-ray, ISC = Indian subcontinent, CRP = C-reactive protein, Ind = indeterminate, ND = not done, N/A = not available, pred = prednisolone. Dyspnoea = breathlessness. Haemoptysis = coughing up blood. CURB65 score = pneumonia severity score where 5 is the most severe. HT = hypertension. DM = hypertension. Adeno = adenocarcinoma. (PPTX) [file pone.0070630.s013.pptx]

## Slide 1
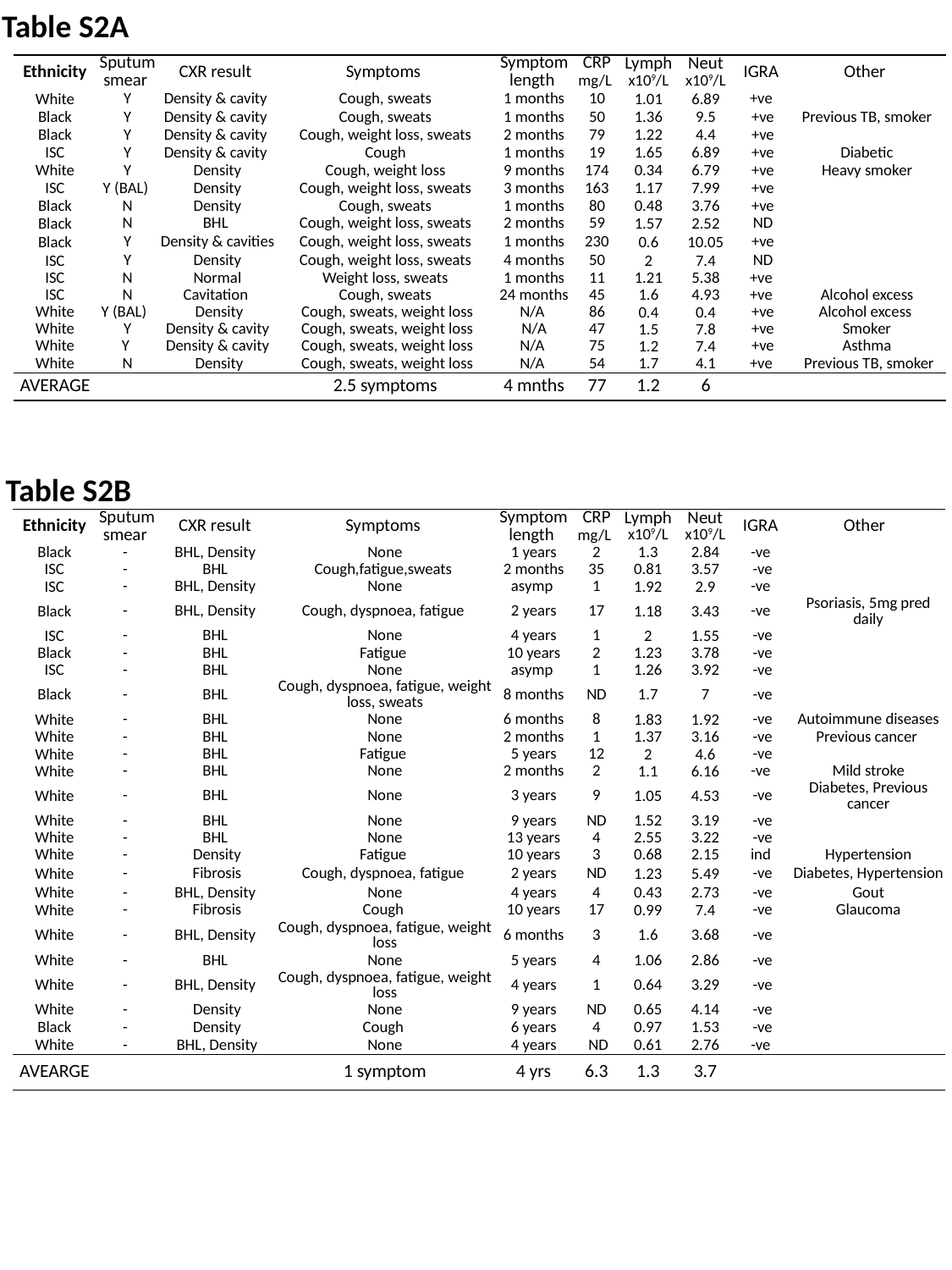

Table S2A
| Ethnicity | Sputum smear | CXR result | Symptoms | Symptom length | CRP mg/L | Lymph x109/L | Neut x109/L | IGRA | Other |
| --- | --- | --- | --- | --- | --- | --- | --- | --- | --- |
| White | Y | Density & cavity | Cough, sweats | 1 months | 10 | 1.01 | 6.89 | +ve | |
| Black | Y | Density & cavity | Cough, sweats | 1 months | 50 | 1.36 | 9.5 | +ve | Previous TB, smoker |
| Black | Y | Density & cavity | Cough, weight loss, sweats | 2 months | 79 | 1.22 | 4.4 | +ve | |
| ISC | Y | Density & cavity | Cough | 1 months | 19 | 1.65 | 6.89 | +ve | Diabetic |
| White | Y | Density | Cough, weight loss | 9 months | 174 | 0.34 | 6.79 | +ve | Heavy smoker |
| ISC | Y (BAL) | Density | Cough, weight loss, sweats | 3 months | 163 | 1.17 | 7.99 | +ve | |
| Black | N | Density | Cough, sweats | 1 months | 80 | 0.48 | 3.76 | +ve | |
| Black | N | BHL | Cough, weight loss, sweats | 2 months | 59 | 1.57 | 2.52 | ND | |
| Black | Y | Density & cavities | Cough, weight loss, sweats | 1 months | 230 | 0.6 | 10.05 | +ve | |
| ISC | Y | Density | Cough, weight loss, sweats | 4 months | 50 | 2 | 7.4 | ND | |
| ISC | N | Normal | Weight loss, sweats | 1 months | 11 | 1.21 | 5.38 | +ve | |
| ISC | N | Cavitation | Cough, sweats | 24 months | 45 | 1.6 | 4.93 | +ve | Alcohol excess |
| White | Y (BAL) | Density | Cough, sweats, weight loss | N/A | 86 | 0.4 | 0.4 | +ve | Alcohol excess |
| White | Y | Density & cavity | Cough, sweats, weight loss | N/A | 47 | 1.5 | 7.8 | +ve | Smoker |
| White | Y | Density & cavity | Cough, sweats, weight loss | N/A | 75 | 1.2 | 7.4 | +ve | Asthma |
| White | N | Density | Cough, sweats, weight loss | N/A | 54 | 1.7 | 4.1 | +ve | Previous TB, smoker |
| AVERAGE | | | 2.5 symptoms | 4 mnths | 77 | 1.2 | 6 | | |
Table S2B
| Ethnicity | Sputum smear | CXR result | Symptoms | Symptom length | CRP mg/L | Lymph x109/L | Neut x109/L | IGRA | Other |
| --- | --- | --- | --- | --- | --- | --- | --- | --- | --- |
| Black | - | BHL, Density | None | 1 years | 2 | 1.3 | 2.84 | -ve | |
| ISC | - | BHL | Cough,fatigue,sweats | 2 months | 35 | 0.81 | 3.57 | -ve | |
| ISC | - | BHL, Density | None | asymp | 1 | 1.92 | 2.9 | -ve | |
| Black | - | BHL, Density | Cough, dyspnoea, fatigue | 2 years | 17 | 1.18 | 3.43 | -ve | Psoriasis, 5mg pred daily |
| ISC | - | BHL | None | 4 years | 1 | 2 | 1.55 | -ve | |
| Black | - | BHL | Fatigue | 10 years | 2 | 1.23 | 3.78 | -ve | |
| ISC | - | BHL | None | asymp | 1 | 1.26 | 3.92 | -ve | |
| Black | - | BHL | Cough, dyspnoea, fatigue, weight loss, sweats | 8 months | ND | 1.7 | 7 | -ve | |
| White | - | BHL | None | 6 months | 8 | 1.83 | 1.92 | -ve | Autoimmune diseases |
| White | - | BHL | None | 2 months | 1 | 1.37 | 3.16 | -ve | Previous cancer |
| White | - | BHL | Fatigue | 5 years | 12 | 2 | 4.6 | -ve | |
| White | - | BHL | None | 2 months | 2 | 1.1 | 6.16 | -ve | Mild stroke |
| White | - | BHL | None | 3 years | 9 | 1.05 | 4.53 | -ve | Diabetes, Previous cancer |
| White | - | BHL | None | 9 years | ND | 1.52 | 3.19 | -ve | |
| White | - | BHL | None | 13 years | 4 | 2.55 | 3.22 | -ve | |
| White | - | Density | Fatigue | 10 years | 3 | 0.68 | 2.15 | ind | Hypertension |
| White | - | Fibrosis | Cough, dyspnoea, fatigue | 2 years | ND | 1.23 | 5.49 | -ve | Diabetes, Hypertension |
| White | - | BHL, Density | None | 4 years | 4 | 0.43 | 2.73 | -ve | Gout |
| White | - | Fibrosis | Cough | 10 years | 17 | 0.99 | 7.4 | -ve | Glaucoma |
| White | - | BHL, Density | Cough, dyspnoea, fatigue, weight loss | 6 months | 3 | 1.6 | 3.68 | -ve | |
| White | - | BHL | None | 5 years | 4 | 1.06 | 2.86 | -ve | |
| White | - | BHL, Density | Cough, dyspnoea, fatigue, weight loss | 4 years | 1 | 0.64 | 3.29 | -ve | |
| White | - | Density | None | 9 years | ND | 0.65 | 4.14 | -ve | |
| Black | - | Density | Cough | 6 years | 4 | 0.97 | 1.53 | -ve | |
| White | - | BHL, Density | None | 4 years | ND | 0.61 | 2.76 | -ve | |
| AVEARGE | | | 1 symptom | 4 yrs | 6.3 | 1.3 | 3.7 | | |

## Slide 2
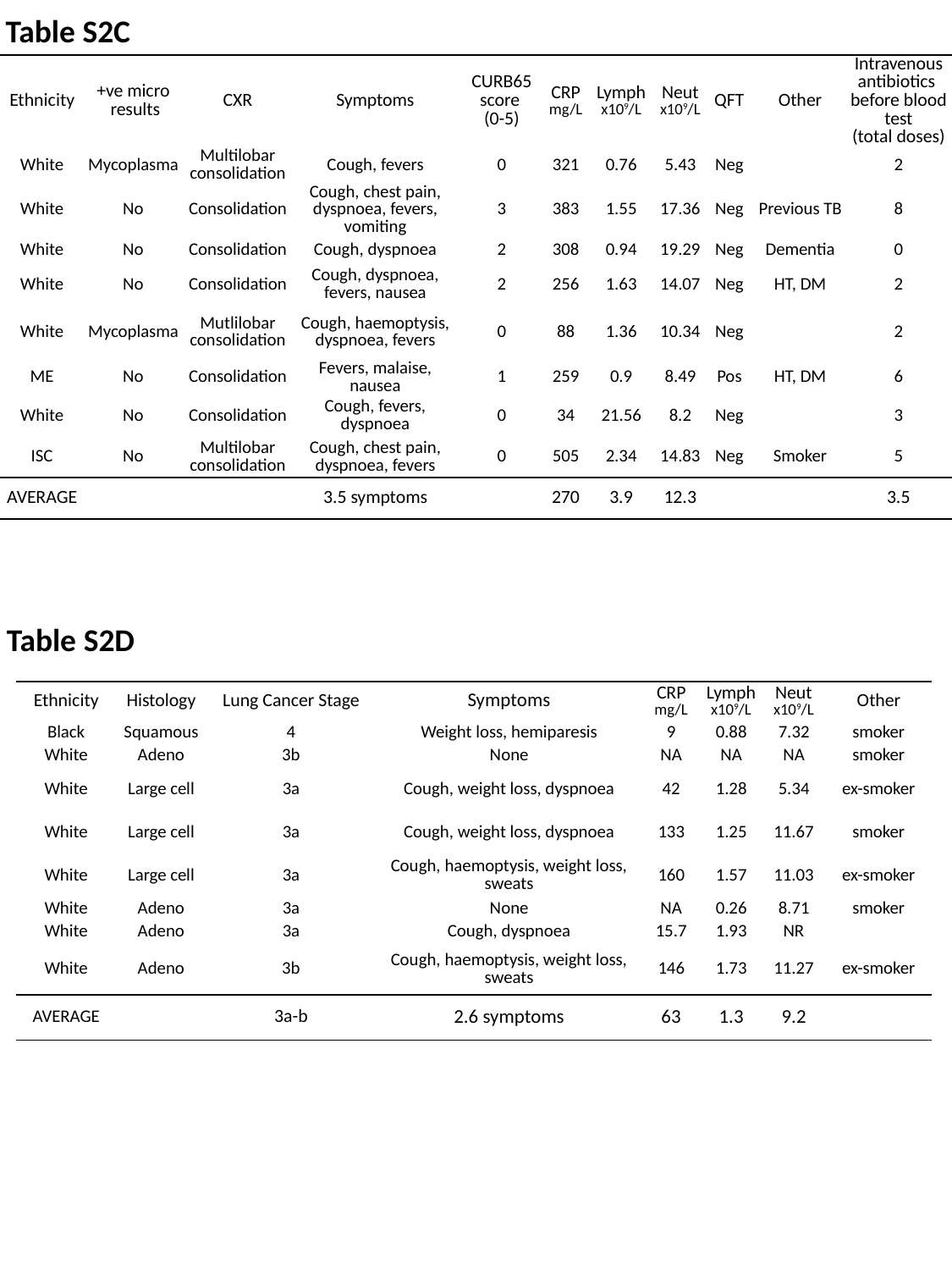

Table S2C
| Ethnicity | +ve micro results | CXR | Symptoms | CURB65 score (0-5) | CRP mg/L | Lymph x109/L | Neut x109/L | QFT | Other | Intravenous antibiotics before blood test (total doses) |
| --- | --- | --- | --- | --- | --- | --- | --- | --- | --- | --- |
| White | Mycoplasma | Multilobar consolidation | Cough, fevers | 0 | 321 | 0.76 | 5.43 | Neg | | 2 |
| White | No | Consolidation | Cough, chest pain, dyspnoea, fevers, vomiting | 3 | 383 | 1.55 | 17.36 | Neg | Previous TB | 8 |
| White | No | Consolidation | Cough, dyspnoea | 2 | 308 | 0.94 | 19.29 | Neg | Dementia | 0 |
| White | No | Consolidation | Cough, dyspnoea, fevers, nausea | 2 | 256 | 1.63 | 14.07 | Neg | HT, DM | 2 |
| White | Mycoplasma | Mutlilobar consolidation | Cough, haemoptysis, dyspnoea, fevers | 0 | 88 | 1.36 | 10.34 | Neg | | 2 |
| ME | No | Consolidation | Fevers, malaise, nausea | 1 | 259 | 0.9 | 8.49 | Pos | HT, DM | 6 |
| White | No | Consolidation | Cough, fevers, dyspnoea | 0 | 34 | 21.56 | 8.2 | Neg | | 3 |
| ISC | No | Multilobar consolidation | Cough, chest pain, dyspnoea, fevers | 0 | 505 | 2.34 | 14.83 | Neg | Smoker | 5 |
| AVERAGE | | | 3.5 symptoms | | 270 | 3.9 | 12.3 | | | 3.5 |
Table S2D
| Ethnicity | Histology | Lung Cancer Stage | Symptoms | CRP mg/L | Lymph x109/L | Neut x109/L | Other |
| --- | --- | --- | --- | --- | --- | --- | --- |
| Black | Squamous | 4 | Weight loss, hemiparesis | 9 | 0.88 | 7.32 | smoker |
| White | Adeno | 3b | None | NA | NA | NA | smoker |
| White | Large cell | 3a | Cough, weight loss, dyspnoea | 42 | 1.28 | 5.34 | ex-smoker |
| White | Large cell | 3a | Cough, weight loss, dyspnoea | 133 | 1.25 | 11.67 | smoker |
| White | Large cell | 3a | Cough, haemoptysis, weight loss, sweats | 160 | 1.57 | 11.03 | ex-smoker |
| White | Adeno | 3a | None | NA | 0.26 | 8.71 | smoker |
| White | Adeno | 3a | Cough, dyspnoea | 15.7 | 1.93 | NR | |
| White | Adeno | 3b | Cough, haemoptysis, weight loss, sweats | 146 | 1.73 | 11.27 | ex-smoker |
| AVERAGE | | 3a-b | 2.6 symptoms | 63 | 1.3 | 9.2 | |

## Slide 3
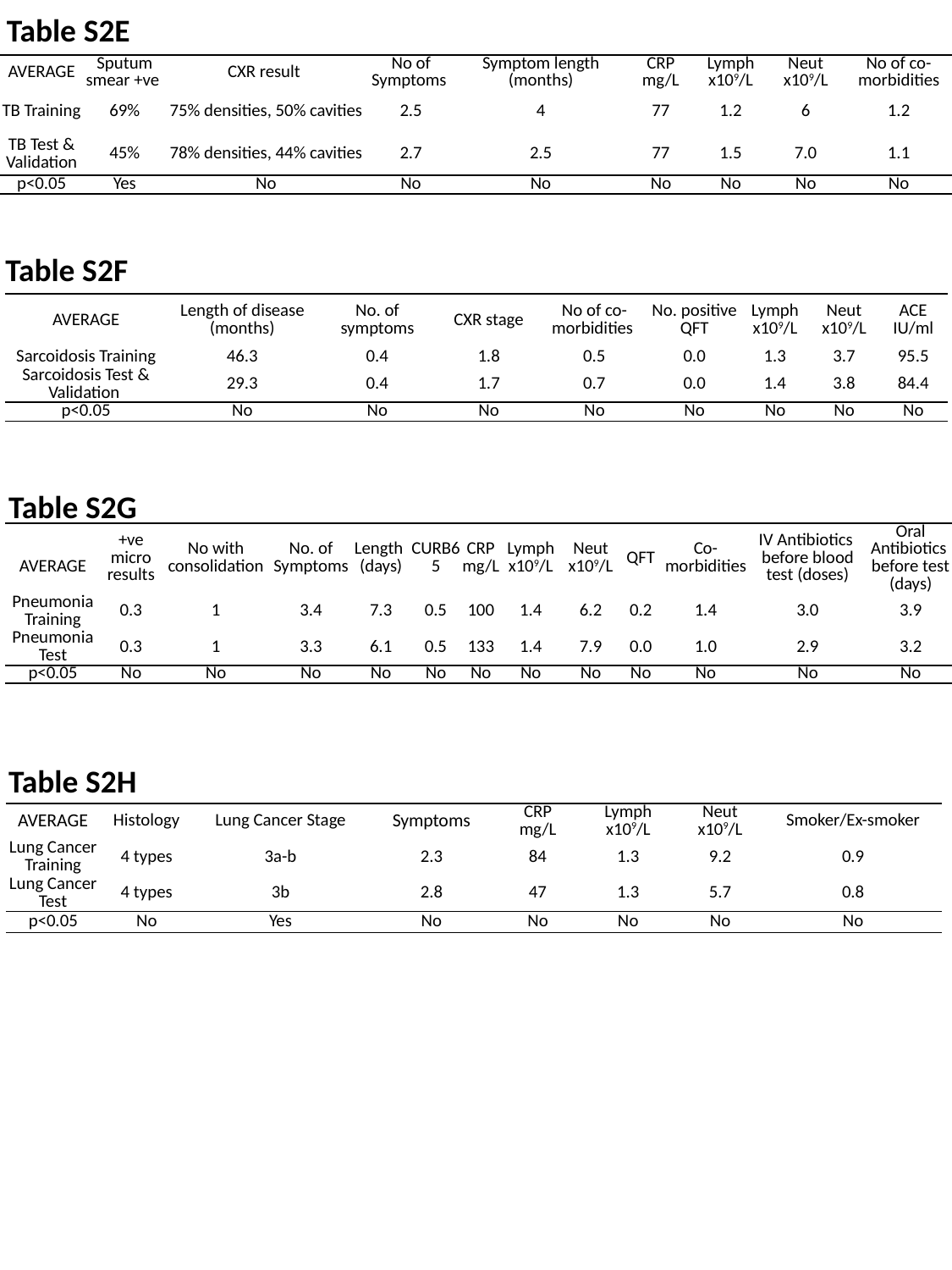

Table S2E
| AVERAGE | Sputum smear +ve | CXR result | No of Symptoms | Symptom length (months) | CRP mg/L | Lymph x109/L | Neut x109/L | No of co-morbidities |
| --- | --- | --- | --- | --- | --- | --- | --- | --- |
| TB Training | 69% | 75% densities, 50% cavities | 2.5 | 4 | 77 | 1.2 | 6 | 1.2 |
| TB Test & Validation | 45% | 78% densities, 44% cavities | 2.7 | 2.5 | 77 | 1.5 | 7.0 | 1.1 |
| p<0.05 | Yes | No | No | No | No | No | No | No |
Table S2F
| AVERAGE | Length of disease (months) | No. of symptoms | CXR stage | No of co-morbidities | No. positive QFT | Lymph x109/L | Neut x109/L | ACE IU/ml |
| --- | --- | --- | --- | --- | --- | --- | --- | --- |
| Sarcoidosis Training | 46.3 | 0.4 | 1.8 | 0.5 | 0.0 | 1.3 | 3.7 | 95.5 |
| Sarcoidosis Test & Validation | 29.3 | 0.4 | 1.7 | 0.7 | 0.0 | 1.4 | 3.8 | 84.4 |
| p<0.05 | No | No | No | No | No | No | No | No |
Table S2G
| AVERAGE | +ve micro results | No with consolidation | No. of Symptoms | Length (days) | CURB65 | CRP mg/L | Lymph x109/L | Neut x109/L | QFT | Co-morbidities | IV Antibiotics before blood test (doses) | Oral Antibiotics before test (days) |
| --- | --- | --- | --- | --- | --- | --- | --- | --- | --- | --- | --- | --- |
| Pneumonia Training | 0.3 | 1 | 3.4 | 7.3 | 0.5 | 100 | 1.4 | 6.2 | 0.2 | 1.4 | 3.0 | 3.9 |
| Pneumonia Test | 0.3 | 1 | 3.3 | 6.1 | 0.5 | 133 | 1.4 | 7.9 | 0.0 | 1.0 | 2.9 | 3.2 |
| p<0.05 | No | No | No | No | No | No | No | No | No | No | No | No |
Table S2H
| AVERAGE | Histology | Lung Cancer Stage | Symptoms | CRP mg/L | Lymph x109/L | Neut x109/L | Smoker/Ex-smoker |
| --- | --- | --- | --- | --- | --- | --- | --- |
| Lung Cancer Training | 4 types | 3a-b | 2.3 | 84 | 1.3 | 9.2 | 0.9 |
| Lung Cancer Test | 4 types | 3b | 2.8 | 47 | 1.3 | 5.7 | 0.8 |
| p<0.05 | No | Yes | No | No | No | No | No |
