# Supplementary material for: Transcriptional Blood Signatures Distinguish Pulmonary Tuberculosis, Pulmonary Sarcoidosis, Pneumonias and Lung Cancers
Source: PLoS One. 2013 Aug 5;8(8):e70630. doi: 10.1371/journal.pone.0070630 (PMC3734176; doi:10.1371/journal.pone.0070630)
Supplement: Table S3 — Clinical characteristics and clinical classification of sarcoidosis patients as determine by the decision tree. (A) Training Set (B) Test Set (C) Validation Set (D) Demographics of all sarcoidosis patients in the three datasets. CXR = chest radiograph, CT = computer tomography, ACE = angiotensin converting enzyme, Lymph = lymphocyte count, Neut = neutrophil count, TLCO = transfer factor for carbon monoxide, KCO = transfer coefficient, FVC = forced vital capacity, FEV1 = forced expiratory volume in 1 second, Abdo = abdomen, LN = lymph node, Med = mediastinal, NA = non-active sarcoidosis, AET = active extra-thoracic sarcoidosis, Neuro = neurological disease. (PPTX) [file pone.0070630.s014.pptx]

## Slide 1
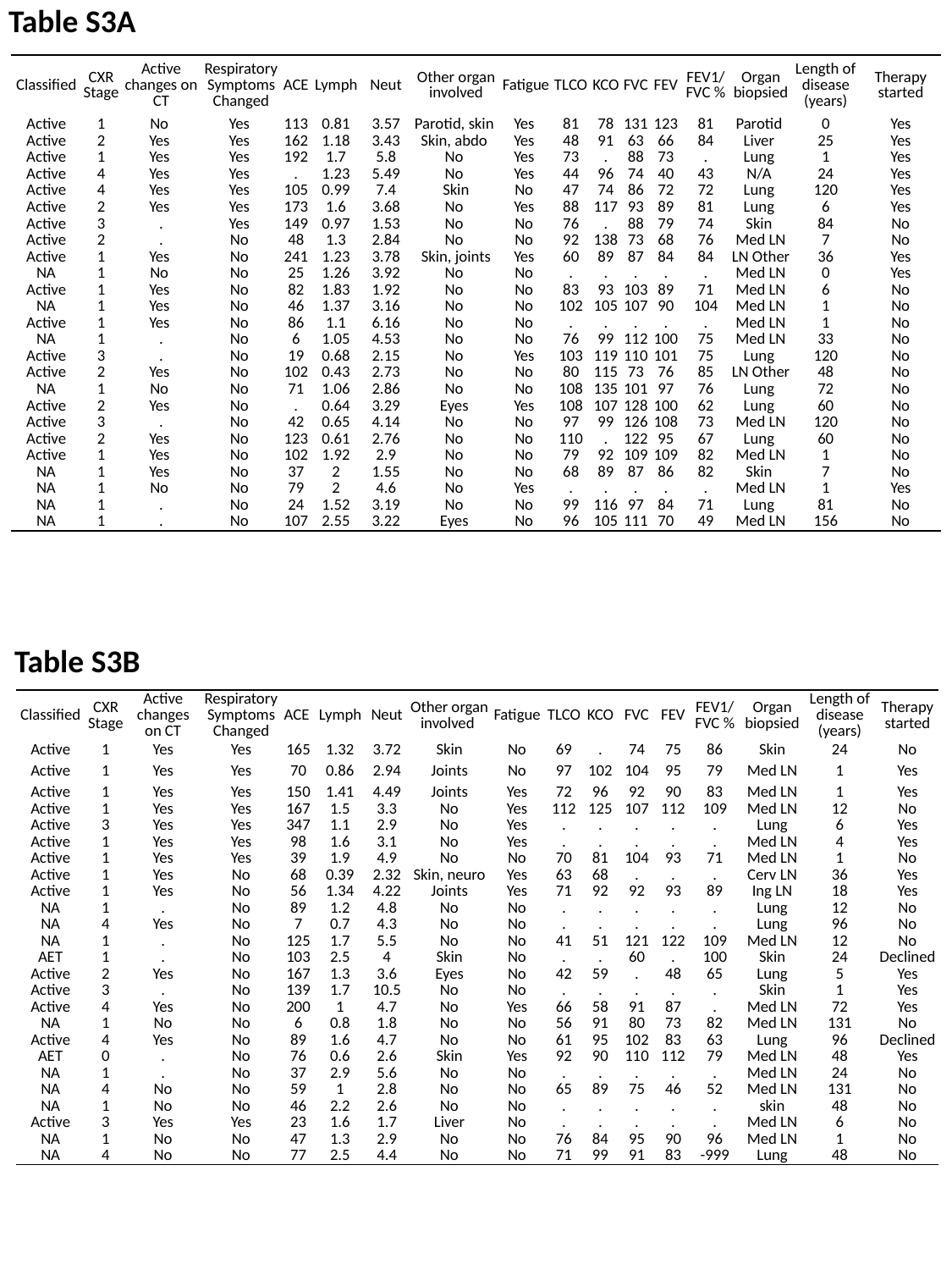

Table S3A
| Classified | CXR Stage | Active changes on CT | Respiratory Symptoms Changed | ACE | Lymph | Neut | Other organ involved | Fatigue | TLCO | KCO | FVC | FEV | FEV1/ FVC % | Organ biopsied | Length of disease (years) | Therapy started |
| --- | --- | --- | --- | --- | --- | --- | --- | --- | --- | --- | --- | --- | --- | --- | --- | --- |
| Active | 1 | No | Yes | 113 | 0.81 | 3.57 | Parotid, skin | Yes | 81 | 78 | 131 | 123 | 81 | Parotid | 0 | Yes |
| Active | 2 | Yes | Yes | 162 | 1.18 | 3.43 | Skin, abdo | Yes | 48 | 91 | 63 | 66 | 84 | Liver | 25 | Yes |
| Active | 1 | Yes | Yes | 192 | 1.7 | 5.8 | No | Yes | 73 | . | 88 | 73 | . | Lung | 1 | Yes |
| Active | 4 | Yes | Yes | . | 1.23 | 5.49 | No | Yes | 44 | 96 | 74 | 40 | 43 | N/A | 24 | Yes |
| Active | 4 | Yes | Yes | 105 | 0.99 | 7.4 | Skin | No | 47 | 74 | 86 | 72 | 72 | Lung | 120 | Yes |
| Active | 2 | Yes | Yes | 173 | 1.6 | 3.68 | No | Yes | 88 | 117 | 93 | 89 | 81 | Lung | 6 | Yes |
| Active | 3 | . | Yes | 149 | 0.97 | 1.53 | No | No | 76 | . | 88 | 79 | 74 | Skin | 84 | No |
| Active | 2 | . | No | 48 | 1.3 | 2.84 | No | No | 92 | 138 | 73 | 68 | 76 | Med LN | 7 | No |
| Active | 1 | Yes | No | 241 | 1.23 | 3.78 | Skin, joints | Yes | 60 | 89 | 87 | 84 | 84 | LN Other | 36 | Yes |
| NA | 1 | No | No | 25 | 1.26 | 3.92 | No | No | . | . | . | . | . | Med LN | 0 | Yes |
| Active | 1 | Yes | No | 82 | 1.83 | 1.92 | No | No | 83 | 93 | 103 | 89 | 71 | Med LN | 6 | No |
| NA | 1 | Yes | No | 46 | 1.37 | 3.16 | No | No | 102 | 105 | 107 | 90 | 104 | Med LN | 1 | No |
| Active | 1 | Yes | No | 86 | 1.1 | 6.16 | No | No | . | . | . | . | . | Med LN | 1 | No |
| NA | 1 | . | No | 6 | 1.05 | 4.53 | No | No | 76 | 99 | 112 | 100 | 75 | Med LN | 33 | No |
| Active | 3 | . | No | 19 | 0.68 | 2.15 | No | Yes | 103 | 119 | 110 | 101 | 75 | Lung | 120 | No |
| Active | 2 | Yes | No | 102 | 0.43 | 2.73 | No | No | 80 | 115 | 73 | 76 | 85 | LN Other | 48 | No |
| NA | 1 | No | No | 71 | 1.06 | 2.86 | No | No | 108 | 135 | 101 | 97 | 76 | Lung | 72 | No |
| Active | 2 | Yes | No | . | 0.64 | 3.29 | Eyes | Yes | 108 | 107 | 128 | 100 | 62 | Lung | 60 | No |
| Active | 3 | . | No | 42 | 0.65 | 4.14 | No | No | 97 | 99 | 126 | 108 | 73 | Med LN | 120 | No |
| Active | 2 | Yes | No | 123 | 0.61 | 2.76 | No | No | 110 | . | 122 | 95 | 67 | Lung | 60 | No |
| Active | 1 | Yes | No | 102 | 1.92 | 2.9 | No | No | 79 | 92 | 109 | 109 | 82 | Med LN | 1 | No |
| NA | 1 | Yes | No | 37 | 2 | 1.55 | No | No | 68 | 89 | 87 | 86 | 82 | Skin | 7 | No |
| NA | 1 | No | No | 79 | 2 | 4.6 | No | Yes | . | . | . | . | . | Med LN | 1 | Yes |
| NA | 1 | . | No | 24 | 1.52 | 3.19 | No | No | 99 | 116 | 97 | 84 | 71 | Lung | 81 | No |
| NA | 1 | . | No | 107 | 2.55 | 3.22 | Eyes | No | 96 | 105 | 111 | 70 | 49 | Med LN | 156 | No |
Table S3B
| Classified | CXR Stage | Active changes on CT | Respiratory Symptoms Changed | ACE | Lymph | Neut | Other organ involved | Fatigue | TLCO | KCO | FVC | FEV | FEV1/ FVC % | Organ biopsied | Length of disease (years) | Therapy started |
| --- | --- | --- | --- | --- | --- | --- | --- | --- | --- | --- | --- | --- | --- | --- | --- | --- |
| Active | 1 | Yes | Yes | 165 | 1.32 | 3.72 | Skin | No | 69 | . | 74 | 75 | 86 | Skin | 24 | No |
| Active | 1 | Yes | Yes | 70 | 0.86 | 2.94 | Joints | No | 97 | 102 | 104 | 95 | 79 | Med LN | 1 | Yes |
| Active | 1 | Yes | Yes | 150 | 1.41 | 4.49 | Joints | Yes | 72 | 96 | 92 | 90 | 83 | Med LN | 1 | Yes |
| Active | 1 | Yes | Yes | 167 | 1.5 | 3.3 | No | Yes | 112 | 125 | 107 | 112 | 109 | Med LN | 12 | No |
| Active | 3 | Yes | Yes | 347 | 1.1 | 2.9 | No | Yes | . | . | . | . | . | Lung | 6 | Yes |
| Active | 1 | Yes | Yes | 98 | 1.6 | 3.1 | No | Yes | . | . | . | . | . | Med LN | 4 | Yes |
| Active | 1 | Yes | Yes | 39 | 1.9 | 4.9 | No | No | 70 | 81 | 104 | 93 | 71 | Med LN | 1 | No |
| Active | 1 | Yes | No | 68 | 0.39 | 2.32 | Skin, neuro | Yes | 63 | 68 | . | . | . | Cerv LN | 36 | Yes |
| Active | 1 | Yes | No | 56 | 1.34 | 4.22 | Joints | Yes | 71 | 92 | 92 | 93 | 89 | Ing LN | 18 | Yes |
| NA | 1 | . | No | 89 | 1.2 | 4.8 | No | No | . | . | . | . | . | Lung | 12 | No |
| NA | 4 | Yes | No | 7 | 0.7 | 4.3 | No | No | . | . | . | . | . | Lung | 96 | No |
| NA | 1 | . | No | 125 | 1.7 | 5.5 | No | No | 41 | 51 | 121 | 122 | 109 | Med LN | 12 | No |
| AET | 1 | . | No | 103 | 2.5 | 4 | Skin | No | . | . | 60 | . | 100 | Skin | 24 | Declined |
| Active | 2 | Yes | No | 167 | 1.3 | 3.6 | Eyes | No | 42 | 59 | . | 48 | 65 | Lung | 5 | Yes |
| Active | 3 | . | No | 139 | 1.7 | 10.5 | No | No | . | . | . | . | . | Skin | 1 | Yes |
| Active | 4 | Yes | No | 200 | 1 | 4.7 | No | Yes | 66 | 58 | 91 | 87 | . | Med LN | 72 | Yes |
| NA | 1 | No | No | 6 | 0.8 | 1.8 | No | No | 56 | 91 | 80 | 73 | 82 | Med LN | 131 | No |
| Active | 4 | Yes | No | 89 | 1.6 | 4.7 | No | No | 61 | 95 | 102 | 83 | 63 | Lung | 96 | Declined |
| AET | 0 | . | No | 76 | 0.6 | 2.6 | Skin | Yes | 92 | 90 | 110 | 112 | 79 | Med LN | 48 | Yes |
| NA | 1 | . | No | 37 | 2.9 | 5.6 | No | No | . | . | . | . | . | Med LN | 24 | No |
| NA | 4 | No | No | 59 | 1 | 2.8 | No | No | 65 | 89 | 75 | 46 | 52 | Med LN | 131 | No |
| NA | 1 | No | No | 46 | 2.2 | 2.6 | No | No | . | . | . | . | . | skin | 48 | No |
| Active | 3 | Yes | Yes | 23 | 1.6 | 1.7 | Liver | No | . | . | . | . | . | Med LN | 6 | No |
| NA | 1 | No | No | 47 | 1.3 | 2.9 | No | No | 76 | 84 | 95 | 90 | 96 | Med LN | 1 | No |
| NA | 4 | No | No | 77 | 2.5 | 4.4 | No | No | 71 | 99 | 91 | 83 | -999 | Lung | 48 | No |

## Slide 2
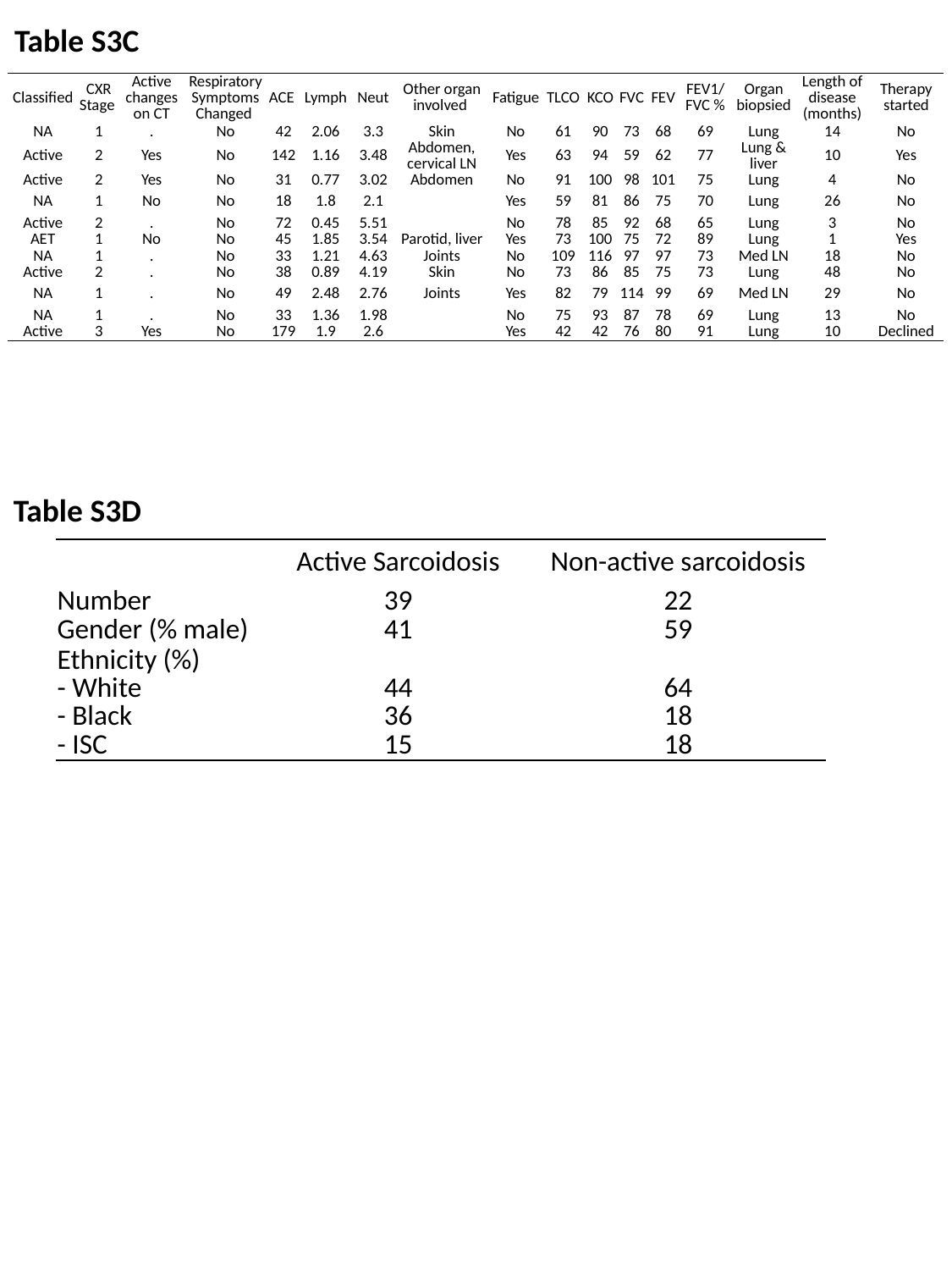

Table S3C
| Classified | CXR Stage | Active changes on CT | Respiratory Symptoms Changed | ACE | Lymph | Neut | Other organ involved | Fatigue | TLCO | KCO | FVC | FEV | FEV1/ FVC % | Organ biopsied | Length of disease (months) | Therapy started |
| --- | --- | --- | --- | --- | --- | --- | --- | --- | --- | --- | --- | --- | --- | --- | --- | --- |
| NA | 1 | . | No | 42 | 2.06 | 3.3 | Skin | No | 61 | 90 | 73 | 68 | 69 | Lung | 14 | No |
| Active | 2 | Yes | No | 142 | 1.16 | 3.48 | Abdomen, cervical LN | Yes | 63 | 94 | 59 | 62 | 77 | Lung & liver | 10 | Yes |
| Active | 2 | Yes | No | 31 | 0.77 | 3.02 | Abdomen | No | 91 | 100 | 98 | 101 | 75 | Lung | 4 | No |
| NA | 1 | No | No | 18 | 1.8 | 2.1 | | Yes | 59 | 81 | 86 | 75 | 70 | Lung | 26 | No |
| Active | 2 | . | No | 72 | 0.45 | 5.51 | | No | 78 | 85 | 92 | 68 | 65 | Lung | 3 | No |
| AET | 1 | No | No | 45 | 1.85 | 3.54 | Parotid, liver | Yes | 73 | 100 | 75 | 72 | 89 | Lung | 1 | Yes |
| NA | 1 | . | No | 33 | 1.21 | 4.63 | Joints | No | 109 | 116 | 97 | 97 | 73 | Med LN | 18 | No |
| Active | 2 | . | No | 38 | 0.89 | 4.19 | Skin | No | 73 | 86 | 85 | 75 | 73 | Lung | 48 | No |
| NA | 1 | . | No | 49 | 2.48 | 2.76 | Joints | Yes | 82 | 79 | 114 | 99 | 69 | Med LN | 29 | No |
| NA | 1 | . | No | 33 | 1.36 | 1.98 | | No | 75 | 93 | 87 | 78 | 69 | Lung | 13 | No |
| Active | 3 | Yes | No | 179 | 1.9 | 2.6 | | Yes | 42 | 42 | 76 | 80 | 91 | Lung | 10 | Declined |
Table S3D
| | Active Sarcoidosis | Non-active sarcoidosis |
| --- | --- | --- |
| Number | 39 | 22 |
| Gender (% male) | 41 | 59 |
| Ethnicity (%) - White - Black - ISC | 44 36 15 | 64 18 18 |
