# Supplementary material for: Transcriptional Blood Signatures Distinguish Pulmonary Tuberculosis, Pulmonary Sarcoidosis, Pneumonias and Lung Cancers
Source: PLoS One. 2013 Aug 5;8(8):e70630. doi: 10.1371/journal.pone.0070630 (PMC3734176; doi:10.1371/journal.pone.0070630)
Supplement: Table S4 — The clinical classification decision tree used to categorise patients into either active or non-active sarcoidosis predicts the clustering of the transcriptional profiles of the sarcoidosis patients better than standard single or multiple clinical variables. (A) Univariate regression analysis to determine which single clinical variables can best predict those sarcoidosis patients that will cluster with the TB patients and those that will cluster with the healthy controls as per the unsupervised clustering of the 1446-transcripts in the Training set and Test set (see Figures 1 &S4b) (B) Multivariate analysis to determine the ability of more than one variable to predict the clustering of the sarcoidosis patients. (PPTX) [file pone.0070630.s015.pptx]

## Slide 1
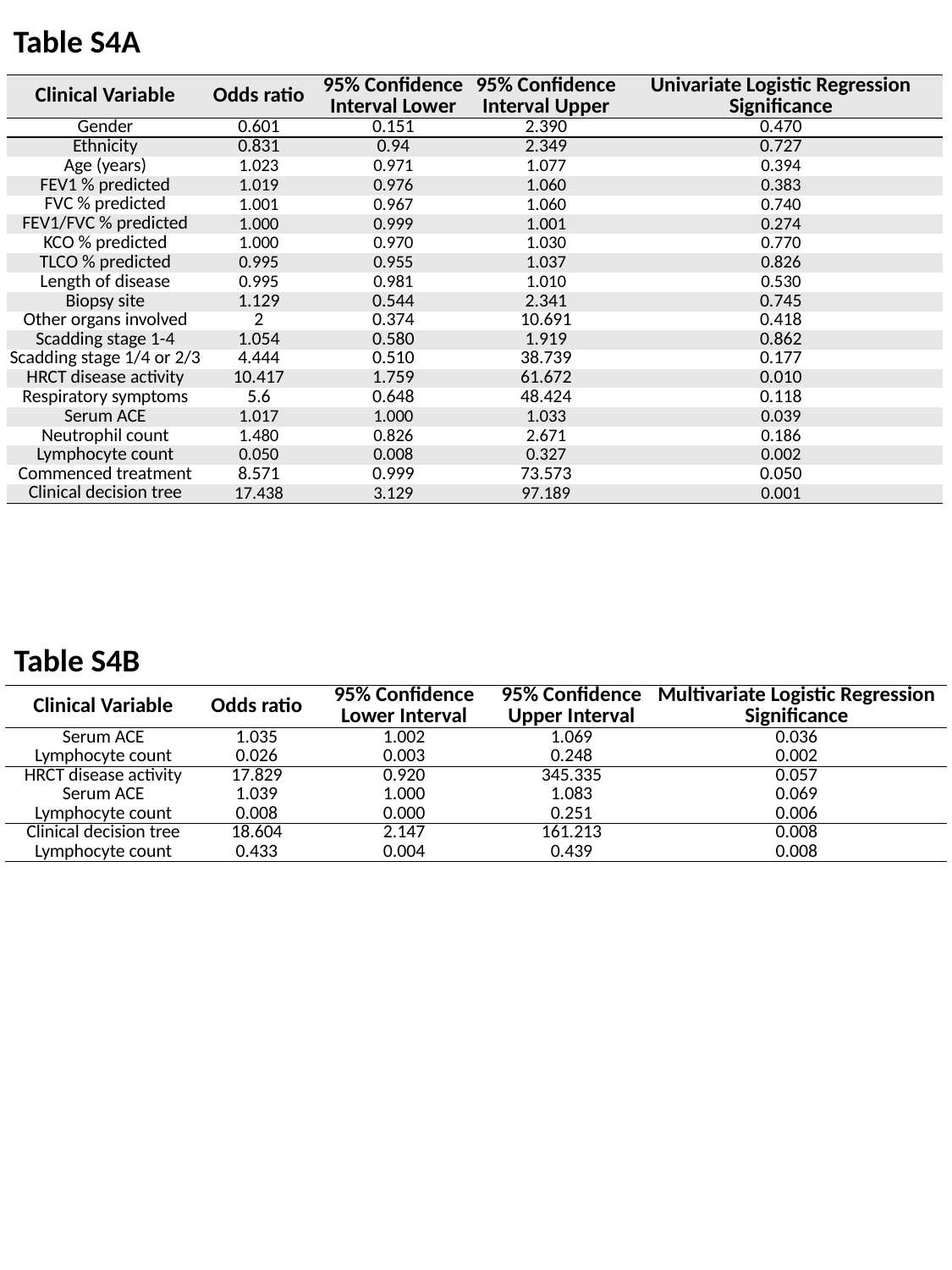

Table S4A
| Clinical Variable | Odds ratio | 95% Confidence Interval Lower | 95% Confidence Interval Upper | Univariate Logistic Regression Significance |
| --- | --- | --- | --- | --- |
| Gender | 0.601 | 0.151 | 2.390 | 0.470 |
| Ethnicity | 0.831 | 0.94 | 2.349 | 0.727 |
| Age (years) | 1.023 | 0.971 | 1.077 | 0.394 |
| FEV1 % predicted | 1.019 | 0.976 | 1.060 | 0.383 |
| FVC % predicted | 1.001 | 0.967 | 1.060 | 0.740 |
| FEV1/FVC % predicted | 1.000 | 0.999 | 1.001 | 0.274 |
| KCO % predicted | 1.000 | 0.970 | 1.030 | 0.770 |
| TLCO % predicted | 0.995 | 0.955 | 1.037 | 0.826 |
| Length of disease | 0.995 | 0.981 | 1.010 | 0.530 |
| Biopsy site | 1.129 | 0.544 | 2.341 | 0.745 |
| Other organs involved | 2 | 0.374 | 10.691 | 0.418 |
| Scadding stage 1-4 | 1.054 | 0.580 | 1.919 | 0.862 |
| Scadding stage 1/4 or 2/3 | 4.444 | 0.510 | 38.739 | 0.177 |
| HRCT disease activity | 10.417 | 1.759 | 61.672 | 0.010 |
| Respiratory symptoms | 5.6 | 0.648 | 48.424 | 0.118 |
| Serum ACE | 1.017 | 1.000 | 1.033 | 0.039 |
| Neutrophil count | 1.480 | 0.826 | 2.671 | 0.186 |
| Lymphocyte count | 0.050 | 0.008 | 0.327 | 0.002 |
| Commenced treatment | 8.571 | 0.999 | 73.573 | 0.050 |
| Clinical decision tree | 17.438 | 3.129 | 97.189 | 0.001 |
Table S4B
| Clinical Variable | Odds ratio | 95% Confidence Lower Interval | 95% Confidence Upper Interval | Multivariate Logistic Regression Significance |
| --- | --- | --- | --- | --- |
| Serum ACE | 1.035 | 1.002 | 1.069 | 0.036 |
| Lymphocyte count | 0.026 | 0.003 | 0.248 | 0.002 |
| HRCT disease activity | 17.829 | 0.920 | 345.335 | 0.057 |
| Serum ACE | 1.039 | 1.000 | 1.083 | 0.069 |
| Lymphocyte count | 0.008 | 0.000 | 0.251 | 0.006 |
| Clinical decision tree | 18.604 | 2.147 | 161.213 | 0.008 |
| Lymphocyte count | 0.433 | 0.004 | 0.439 | 0.008 |
