# Supplementary material for: Transcriptional Blood Signatures Distinguish Pulmonary Tuberculosis, Pulmonary Sarcoidosis, Pneumonias and Lung Cancers
Source: PLoS One. 2013 Aug 5;8(8):e70630. doi: 10.1371/journal.pone.0070630 (PMC3734176; doi:10.1371/journal.pone.0070630)
Supplement: Table S5 — The top 50 differentially expressed transcripts for each disease compared to matched controls. Differentially expressed genes were derived from the Training Set by comparing each disease to healthy controls matched for ethnicity and gender: TB = 2524, active sarcoidosis = 1391, pneumonia = 2801 and lung cancer = 1626 transcripts (≥1.5 fold change from the mean of the controls, Mann Whitney Benjamini Hochberg p<0.01). (PPTX) [file pone.0070630.s016.pptx]

## Slide 1
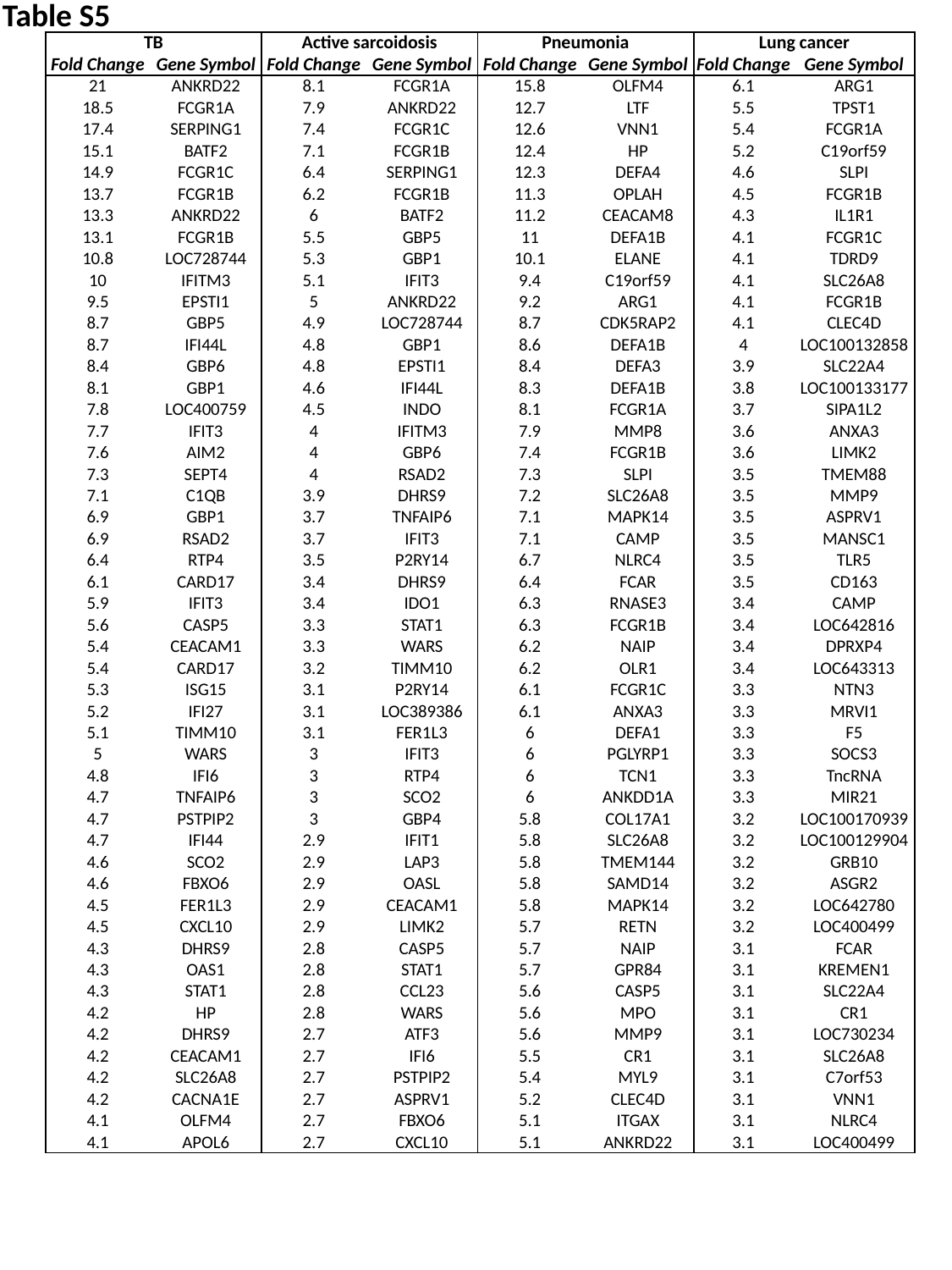

Table S5
| TB | | Active sarcoidosis | | Pneumonia | | Lung cancer | |
| --- | --- | --- | --- | --- | --- | --- | --- |
| Fold Change | Gene Symbol | Fold Change | Gene Symbol | Fold Change | Gene Symbol | Fold Change | Gene Symbol |
| 21 | ANKRD22 | 8.1 | FCGR1A | 15.8 | OLFM4 | 6.1 | ARG1 |
| 18.5 | FCGR1A | 7.9 | ANKRD22 | 12.7 | LTF | 5.5 | TPST1 |
| 17.4 | SERPING1 | 7.4 | FCGR1C | 12.6 | VNN1 | 5.4 | FCGR1A |
| 15.1 | BATF2 | 7.1 | FCGR1B | 12.4 | HP | 5.2 | C19orf59 |
| 14.9 | FCGR1C | 6.4 | SERPING1 | 12.3 | DEFA4 | 4.6 | SLPI |
| 13.7 | FCGR1B | 6.2 | FCGR1B | 11.3 | OPLAH | 4.5 | FCGR1B |
| 13.3 | ANKRD22 | 6 | BATF2 | 11.2 | CEACAM8 | 4.3 | IL1R1 |
| 13.1 | FCGR1B | 5.5 | GBP5 | 11 | DEFA1B | 4.1 | FCGR1C |
| 10.8 | LOC728744 | 5.3 | GBP1 | 10.1 | ELANE | 4.1 | TDRD9 |
| 10 | IFITM3 | 5.1 | IFIT3 | 9.4 | C19orf59 | 4.1 | SLC26A8 |
| 9.5 | EPSTI1 | 5 | ANKRD22 | 9.2 | ARG1 | 4.1 | FCGR1B |
| 8.7 | GBP5 | 4.9 | LOC728744 | 8.7 | CDK5RAP2 | 4.1 | CLEC4D |
| 8.7 | IFI44L | 4.8 | GBP1 | 8.6 | DEFA1B | 4 | LOC100132858 |
| 8.4 | GBP6 | 4.8 | EPSTI1 | 8.4 | DEFA3 | 3.9 | SLC22A4 |
| 8.1 | GBP1 | 4.6 | IFI44L | 8.3 | DEFA1B | 3.8 | LOC100133177 |
| 7.8 | LOC400759 | 4.5 | INDO | 8.1 | FCGR1A | 3.7 | SIPA1L2 |
| 7.7 | IFIT3 | 4 | IFITM3 | 7.9 | MMP8 | 3.6 | ANXA3 |
| 7.6 | AIM2 | 4 | GBP6 | 7.4 | FCGR1B | 3.6 | LIMK2 |
| 7.3 | SEPT4 | 4 | RSAD2 | 7.3 | SLPI | 3.5 | TMEM88 |
| 7.1 | C1QB | 3.9 | DHRS9 | 7.2 | SLC26A8 | 3.5 | MMP9 |
| 6.9 | GBP1 | 3.7 | TNFAIP6 | 7.1 | MAPK14 | 3.5 | ASPRV1 |
| 6.9 | RSAD2 | 3.7 | IFIT3 | 7.1 | CAMP | 3.5 | MANSC1 |
| 6.4 | RTP4 | 3.5 | P2RY14 | 6.7 | NLRC4 | 3.5 | TLR5 |
| 6.1 | CARD17 | 3.4 | DHRS9 | 6.4 | FCAR | 3.5 | CD163 |
| 5.9 | IFIT3 | 3.4 | IDO1 | 6.3 | RNASE3 | 3.4 | CAMP |
| 5.6 | CASP5 | 3.3 | STAT1 | 6.3 | FCGR1B | 3.4 | LOC642816 |
| 5.4 | CEACAM1 | 3.3 | WARS | 6.2 | NAIP | 3.4 | DPRXP4 |
| 5.4 | CARD17 | 3.2 | TIMM10 | 6.2 | OLR1 | 3.4 | LOC643313 |
| 5.3 | ISG15 | 3.1 | P2RY14 | 6.1 | FCGR1C | 3.3 | NTN3 |
| 5.2 | IFI27 | 3.1 | LOC389386 | 6.1 | ANXA3 | 3.3 | MRVI1 |
| 5.1 | TIMM10 | 3.1 | FER1L3 | 6 | DEFA1 | 3.3 | F5 |
| 5 | WARS | 3 | IFIT3 | 6 | PGLYRP1 | 3.3 | SOCS3 |
| 4.8 | IFI6 | 3 | RTP4 | 6 | TCN1 | 3.3 | TncRNA |
| 4.7 | TNFAIP6 | 3 | SCO2 | 6 | ANKDD1A | 3.3 | MIR21 |
| 4.7 | PSTPIP2 | 3 | GBP4 | 5.8 | COL17A1 | 3.2 | LOC100170939 |
| 4.7 | IFI44 | 2.9 | IFIT1 | 5.8 | SLC26A8 | 3.2 | LOC100129904 |
| 4.6 | SCO2 | 2.9 | LAP3 | 5.8 | TMEM144 | 3.2 | GRB10 |
| 4.6 | FBXO6 | 2.9 | OASL | 5.8 | SAMD14 | 3.2 | ASGR2 |
| 4.5 | FER1L3 | 2.9 | CEACAM1 | 5.8 | MAPK14 | 3.2 | LOC642780 |
| 4.5 | CXCL10 | 2.9 | LIMK2 | 5.7 | RETN | 3.2 | LOC400499 |
| 4.3 | DHRS9 | 2.8 | CASP5 | 5.7 | NAIP | 3.1 | FCAR |
| 4.3 | OAS1 | 2.8 | STAT1 | 5.7 | GPR84 | 3.1 | KREMEN1 |
| 4.3 | STAT1 | 2.8 | CCL23 | 5.6 | CASP5 | 3.1 | SLC22A4 |
| 4.2 | HP | 2.8 | WARS | 5.6 | MPO | 3.1 | CR1 |
| 4.2 | DHRS9 | 2.7 | ATF3 | 5.6 | MMP9 | 3.1 | LOC730234 |
| 4.2 | CEACAM1 | 2.7 | IFI6 | 5.5 | CR1 | 3.1 | SLC26A8 |
| 4.2 | SLC26A8 | 2.7 | PSTPIP2 | 5.4 | MYL9 | 3.1 | C7orf53 |
| 4.2 | CACNA1E | 2.7 | ASPRV1 | 5.2 | CLEC4D | 3.1 | VNN1 |
| 4.1 | OLFM4 | 2.7 | FBXO6 | 5.1 | ITGAX | 3.1 | NLRC4 |
| 4.1 | APOL6 | 2.7 | CXCL10 | 5.1 | ANKRD22 | 3.1 | LOC400499 |
