# Supplementary material for: Transcriptional Blood Signatures Distinguish Pulmonary Tuberculosis, Pulmonary Sarcoidosis, Pneumonias and Lung Cancers
Source: PLoS One. 2013 Aug 5;8(8):e70630. doi: 10.1371/journal.pone.0070630 (PMC3734176; doi:10.1371/journal.pone.0070630)
Supplement: Table S6 — The top 50 differentially expressed transcripts unique for each disease as determined by the 4-set Venn diagram. Differentially expressed genes were derived from the Training Set by comparing each disease to healthy controls matched for ethnicity and gender (≥1.5 fold change from the mean of the controls, Mann Whitney Benjamini Hochberg p<0.01). A 4-set Venn diagram was used to identify genes that were unique for each disease. (PPTX) [file pone.0070630.s017.pptx]

## Slide 1
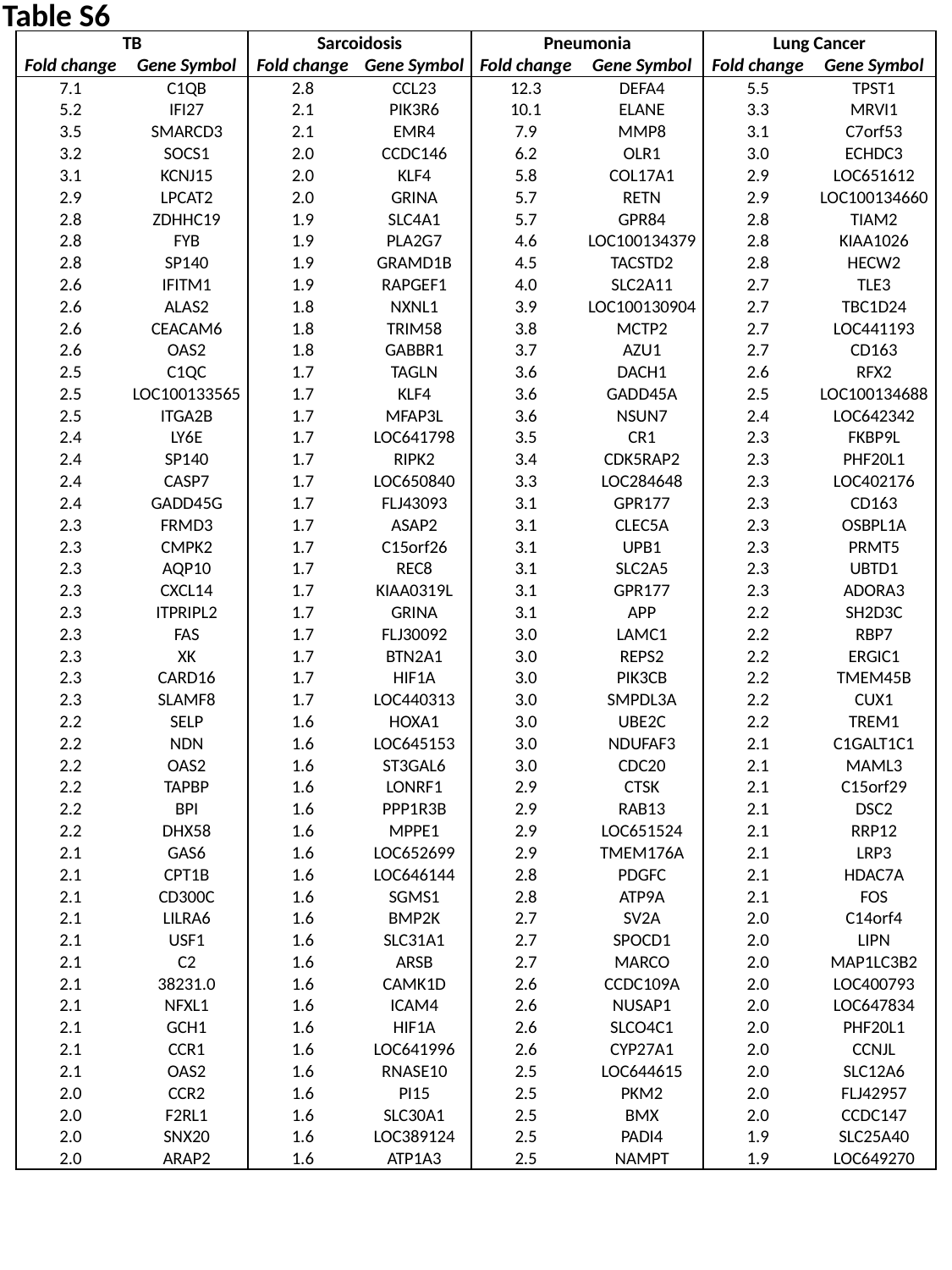

Table S6
| TB | | Sarcoidosis | | Pneumonia | | Lung Cancer | |
| --- | --- | --- | --- | --- | --- | --- | --- |
| Fold change | Gene Symbol | Fold change | Gene Symbol | Fold change | Gene Symbol | Fold change | Gene Symbol |
| 7.1 | C1QB | 2.8 | CCL23 | 12.3 | DEFA4 | 5.5 | TPST1 |
| 5.2 | IFI27 | 2.1 | PIK3R6 | 10.1 | ELANE | 3.3 | MRVI1 |
| 3.5 | SMARCD3 | 2.1 | EMR4 | 7.9 | MMP8 | 3.1 | C7orf53 |
| 3.2 | SOCS1 | 2.0 | CCDC146 | 6.2 | OLR1 | 3.0 | ECHDC3 |
| 3.1 | KCNJ15 | 2.0 | KLF4 | 5.8 | COL17A1 | 2.9 | LOC651612 |
| 2.9 | LPCAT2 | 2.0 | GRINA | 5.7 | RETN | 2.9 | LOC100134660 |
| 2.8 | ZDHHC19 | 1.9 | SLC4A1 | 5.7 | GPR84 | 2.8 | TIAM2 |
| 2.8 | FYB | 1.9 | PLA2G7 | 4.6 | LOC100134379 | 2.8 | KIAA1026 |
| 2.8 | SP140 | 1.9 | GRAMD1B | 4.5 | TACSTD2 | 2.8 | HECW2 |
| 2.6 | IFITM1 | 1.9 | RAPGEF1 | 4.0 | SLC2A11 | 2.7 | TLE3 |
| 2.6 | ALAS2 | 1.8 | NXNL1 | 3.9 | LOC100130904 | 2.7 | TBC1D24 |
| 2.6 | CEACAM6 | 1.8 | TRIM58 | 3.8 | MCTP2 | 2.7 | LOC441193 |
| 2.6 | OAS2 | 1.8 | GABBR1 | 3.7 | AZU1 | 2.7 | CD163 |
| 2.5 | C1QC | 1.7 | TAGLN | 3.6 | DACH1 | 2.6 | RFX2 |
| 2.5 | LOC100133565 | 1.7 | KLF4 | 3.6 | GADD45A | 2.5 | LOC100134688 |
| 2.5 | ITGA2B | 1.7 | MFAP3L | 3.6 | NSUN7 | 2.4 | LOC642342 |
| 2.4 | LY6E | 1.7 | LOC641798 | 3.5 | CR1 | 2.3 | FKBP9L |
| 2.4 | SP140 | 1.7 | RIPK2 | 3.4 | CDK5RAP2 | 2.3 | PHF20L1 |
| 2.4 | CASP7 | 1.7 | LOC650840 | 3.3 | LOC284648 | 2.3 | LOC402176 |
| 2.4 | GADD45G | 1.7 | FLJ43093 | 3.1 | GPR177 | 2.3 | CD163 |
| 2.3 | FRMD3 | 1.7 | ASAP2 | 3.1 | CLEC5A | 2.3 | OSBPL1A |
| 2.3 | CMPK2 | 1.7 | C15orf26 | 3.1 | UPB1 | 2.3 | PRMT5 |
| 2.3 | AQP10 | 1.7 | REC8 | 3.1 | SLC2A5 | 2.3 | UBTD1 |
| 2.3 | CXCL14 | 1.7 | KIAA0319L | 3.1 | GPR177 | 2.3 | ADORA3 |
| 2.3 | ITPRIPL2 | 1.7 | GRINA | 3.1 | APP | 2.2 | SH2D3C |
| 2.3 | FAS | 1.7 | FLJ30092 | 3.0 | LAMC1 | 2.2 | RBP7 |
| 2.3 | XK | 1.7 | BTN2A1 | 3.0 | REPS2 | 2.2 | ERGIC1 |
| 2.3 | CARD16 | 1.7 | HIF1A | 3.0 | PIK3CB | 2.2 | TMEM45B |
| 2.3 | SLAMF8 | 1.7 | LOC440313 | 3.0 | SMPDL3A | 2.2 | CUX1 |
| 2.2 | SELP | 1.6 | HOXA1 | 3.0 | UBE2C | 2.2 | TREM1 |
| 2.2 | NDN | 1.6 | LOC645153 | 3.0 | NDUFAF3 | 2.1 | C1GALT1C1 |
| 2.2 | OAS2 | 1.6 | ST3GAL6 | 3.0 | CDC20 | 2.1 | MAML3 |
| 2.2 | TAPBP | 1.6 | LONRF1 | 2.9 | CTSK | 2.1 | C15orf29 |
| 2.2 | BPI | 1.6 | PPP1R3B | 2.9 | RAB13 | 2.1 | DSC2 |
| 2.2 | DHX58 | 1.6 | MPPE1 | 2.9 | LOC651524 | 2.1 | RRP12 |
| 2.1 | GAS6 | 1.6 | LOC652699 | 2.9 | TMEM176A | 2.1 | LRP3 |
| 2.1 | CPT1B | 1.6 | LOC646144 | 2.8 | PDGFC | 2.1 | HDAC7A |
| 2.1 | CD300C | 1.6 | SGMS1 | 2.8 | ATP9A | 2.1 | FOS |
| 2.1 | LILRA6 | 1.6 | BMP2K | 2.7 | SV2A | 2.0 | C14orf4 |
| 2.1 | USF1 | 1.6 | SLC31A1 | 2.7 | SPOCD1 | 2.0 | LIPN |
| 2.1 | C2 | 1.6 | ARSB | 2.7 | MARCO | 2.0 | MAP1LC3B2 |
| 2.1 | 38231.0 | 1.6 | CAMK1D | 2.6 | CCDC109A | 2.0 | LOC400793 |
| 2.1 | NFXL1 | 1.6 | ICAM4 | 2.6 | NUSAP1 | 2.0 | LOC647834 |
| 2.1 | GCH1 | 1.6 | HIF1A | 2.6 | SLCO4C1 | 2.0 | PHF20L1 |
| 2.1 | CCR1 | 1.6 | LOC641996 | 2.6 | CYP27A1 | 2.0 | CCNJL |
| 2.1 | OAS2 | 1.6 | RNASE10 | 2.5 | LOC644615 | 2.0 | SLC12A6 |
| 2.0 | CCR2 | 1.6 | PI15 | 2.5 | PKM2 | 2.0 | FLJ42957 |
| 2.0 | F2RL1 | 1.6 | SLC30A1 | 2.5 | BMX | 2.0 | CCDC147 |
| 2.0 | SNX20 | 1.6 | LOC389124 | 2.5 | PADI4 | 1.9 | SLC25A40 |
| 2.0 | ARAP2 | 1.6 | ATP1A3 | 2.5 | NAMPT | 1.9 | LOC649270 |
