# Supplementary material for: Transcriptional Blood Signatures Distinguish Pulmonary Tuberculosis, Pulmonary Sarcoidosis, Pneumonias and Lung Cancers
Source: PLoS One. 2013 Aug 5;8(8):e70630. doi: 10.1371/journal.pone.0070630 (PMC3734176; doi:10.1371/journal.pone.0070630)
Supplement: Table S7 — Drug therapy given to each sarcoidosis patient who was commenced on treatment and the clinical management of their practising physician after observing their response to the therapy. Each patients study ID and ‘treatment response category’ correlates with the legend used for the modular analysis. The superscript number of the sarcoidosis patients is used when the patient had more than one visit to their practising physician such that 22 was the visit after being started on treatment and 23 was the subsequent visit. (PPTX) [file pone.0070630.s018.pptx]

## Slide 1
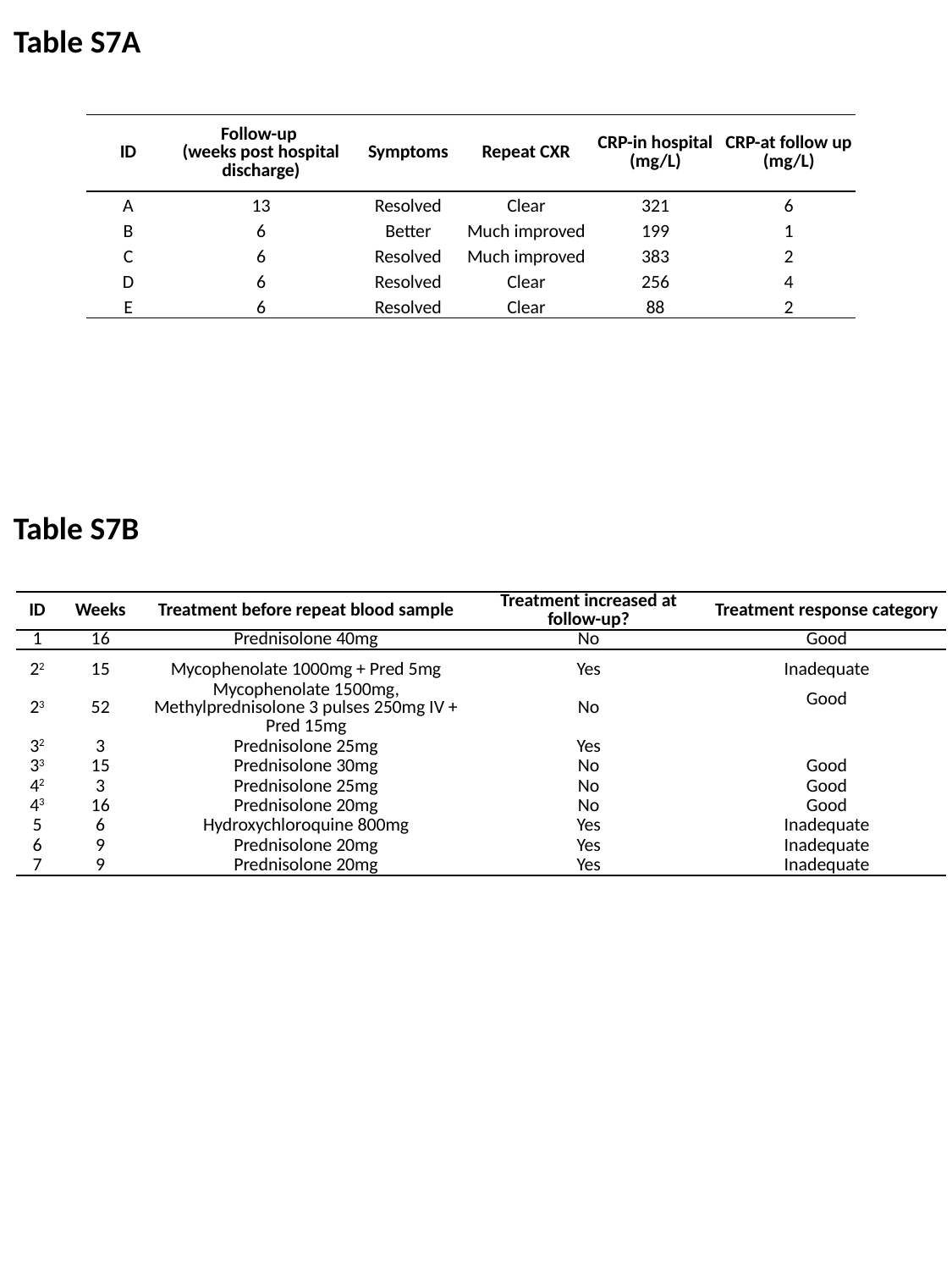

Table S7A
| ID | Follow-up (weeks post hospital discharge) | Symptoms | Repeat CXR | CRP-in hospital (mg/L) | CRP-at follow up (mg/L) |
| --- | --- | --- | --- | --- | --- |
| A | 13 | Resolved | Clear | 321 | 6 |
| B | 6 | Better | Much improved | 199 | 1 |
| C | 6 | Resolved | Much improved | 383 | 2 |
| D | 6 | Resolved | Clear | 256 | 4 |
| E | 6 | Resolved | Clear | 88 | 2 |
Table S7B
| ID | Weeks | Treatment before repeat blood sample | Treatment increased at follow-up? | Treatment response category |
| --- | --- | --- | --- | --- |
| 1 | 16 | Prednisolone 40mg | No | Good |
| 22 | 15 | Mycophenolate 1000mg + Pred 5mg | Yes | Inadequate |
| 23 | 52 | Mycophenolate 1500mg, Methylprednisolone 3 pulses 250mg IV + Pred 15mg | No | Good |
| 32 | 3 | Prednisolone 25mg | Yes | |
| 33 | 15 | Prednisolone 30mg | No | Good |
| 42 | 3 | Prednisolone 25mg | No | Good |
| 43 | 16 | Prednisolone 20mg | No | Good |
| 5 | 6 | Hydroxychloroquine 800mg | Yes | Inadequate |
| 6 | 9 | Prednisolone 20mg | Yes | Inadequate |
| 7 | 9 | Prednisolone 20mg | Yes | Inadequate |
