# Supplementary material for: Transcriptional Blood Signatures Distinguish Pulmonary Tuberculosis, Pulmonary Sarcoidosis, Pneumonias and Lung Cancers
Source: PLoS One. 2013 Aug 5;8(8):e70630. doi: 10.1371/journal.pone.0070630 (PMC3734176; doi:10.1371/journal.pone.0070630)
Supplement: Table S8 — Top 50 over-expressed genes in the inflammation modules in the good-treatment response sarcoidosis patients. (PPTX) [file pone.0070630.s019.pptx]

## Slide 1
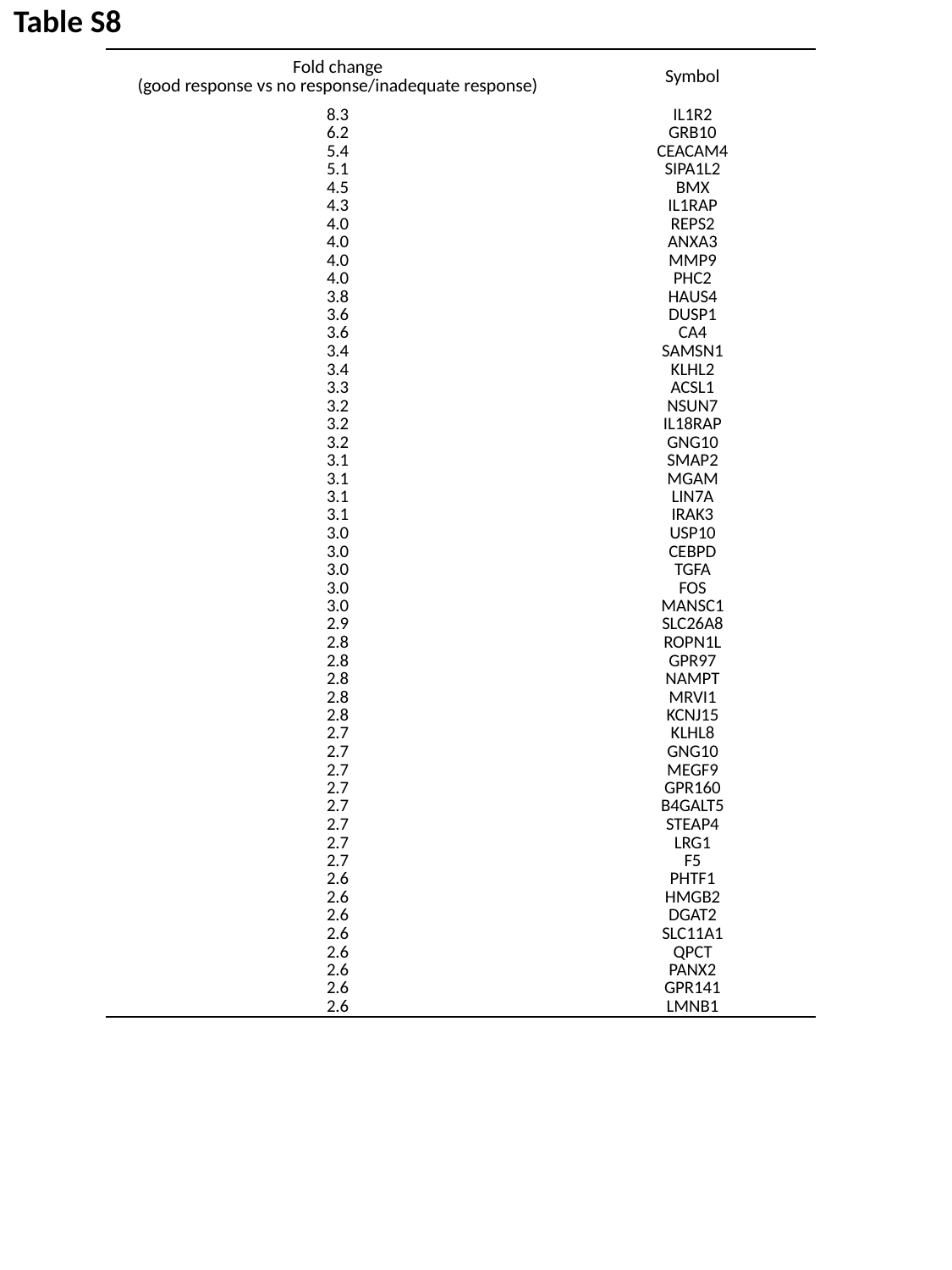

Table S8
| Fold change (good response vs no response/inadequate response) | Symbol |
| --- | --- |
| 8.3 | IL1R2 |
| 6.2 | GRB10 |
| 5.4 | CEACAM4 |
| 5.1 | SIPA1L2 |
| 4.5 | BMX |
| 4.3 | IL1RAP |
| 4.0 | REPS2 |
| 4.0 | ANXA3 |
| 4.0 | MMP9 |
| 4.0 | PHC2 |
| 3.8 | HAUS4 |
| 3.6 | DUSP1 |
| 3.6 | CA4 |
| 3.4 | SAMSN1 |
| 3.4 | KLHL2 |
| 3.3 | ACSL1 |
| 3.2 | NSUN7 |
| 3.2 | IL18RAP |
| 3.2 | GNG10 |
| 3.1 | SMAP2 |
| 3.1 | MGAM |
| 3.1 | LIN7A |
| 3.1 | IRAK3 |
| 3.0 | USP10 |
| 3.0 | CEBPD |
| 3.0 | TGFA |
| 3.0 | FOS |
| 3.0 | MANSC1 |
| 2.9 | SLC26A8 |
| 2.8 | ROPN1L |
| 2.8 | GPR97 |
| 2.8 | NAMPT |
| 2.8 | MRVI1 |
| 2.8 | KCNJ15 |
| 2.7 | KLHL8 |
| 2.7 | GNG10 |
| 2.7 | MEGF9 |
| 2.7 | GPR160 |
| 2.7 | B4GALT5 |
| 2.7 | STEAP4 |
| 2.7 | LRG1 |
| 2.7 | F5 |
| 2.6 | PHTF1 |
| 2.6 | HMGB2 |
| 2.6 | DGAT2 |
| 2.6 | SLC11A1 |
| 2.6 | QPCT |
| 2.6 | PANX2 |
| 2.6 | GPR141 |
| 2.6 | LMNB1 |
