# Supplementary material for: Transcriptional Blood Signatures Distinguish Pulmonary Tuberculosis, Pulmonary Sarcoidosis, Pneumonias and Lung Cancers
Source: PLoS One. 2013 Aug 5;8(8):e70630. doi: 10.1371/journal.pone.0070630 (PMC3734176; doi:10.1371/journal.pone.0070630)
Supplement: Table S9 — Interferon-inducible genes from the Berry et al 2010 publication. (PPTX) [file pone.0070630.s020.pptx]

## Slide 1
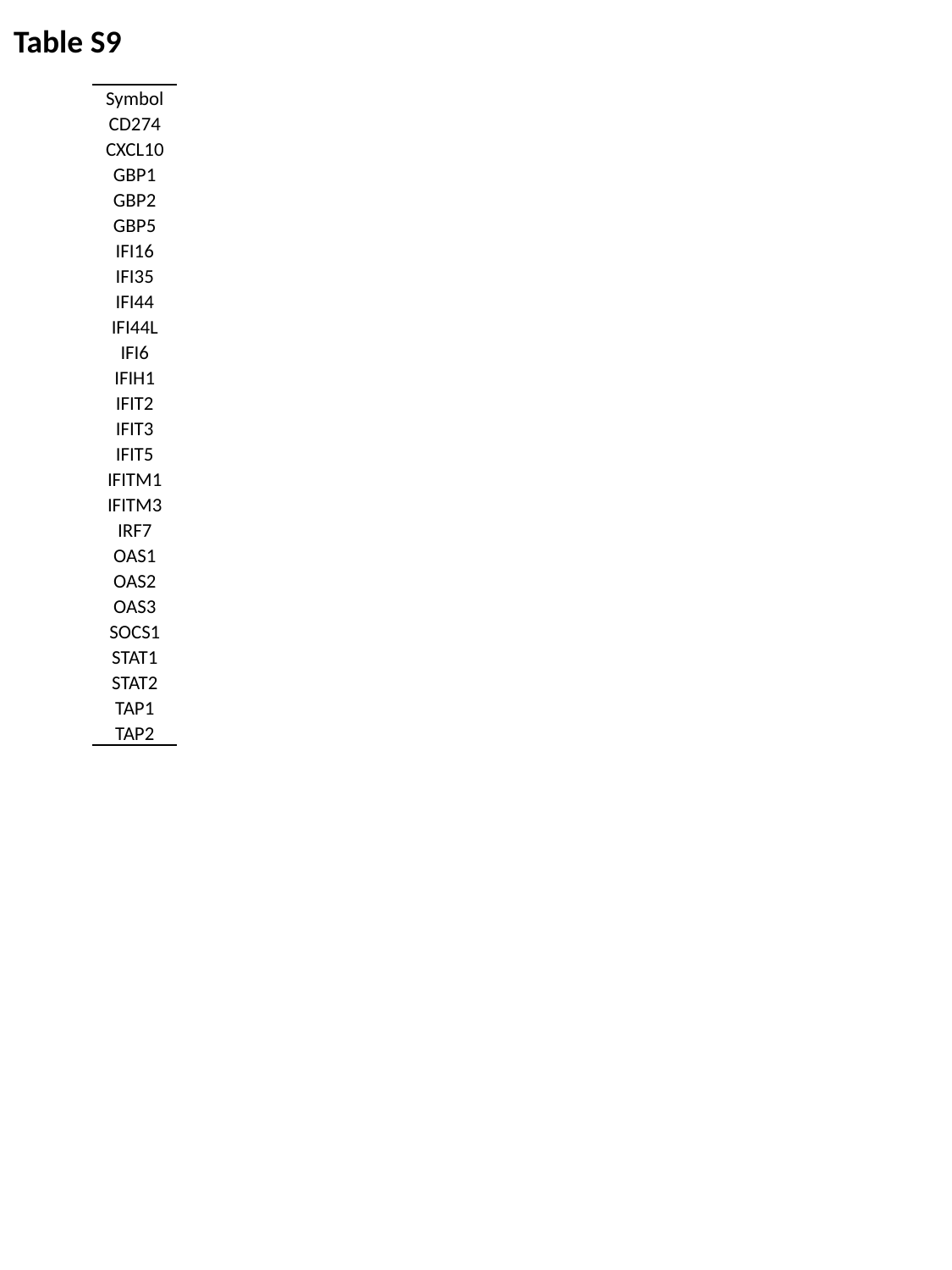

Table S9
| Symbol |
| --- |
| CD274 |
| CXCL10 |
| GBP1 |
| GBP2 |
| GBP5 |
| IFI16 |
| IFI35 |
| IFI44 |
| IFI44L |
| IFI6 |
| IFIH1 |
| IFIT2 |
| IFIT3 |
| IFIT5 |
| IFITM1 |
| IFITM3 |
| IRF7 |
| OAS1 |
| OAS2 |
| OAS3 |
| SOCS1 |
| STAT1 |
| STAT2 |
| TAP1 |
| TAP2 |
