# Supplementary material for: Transcriptional Blood Signatures Distinguish Pulmonary Tuberculosis, Pulmonary Sarcoidosis, Pneumonias and Lung Cancers
Source: PLoS One. 2013 Aug 5;8(8):e70630. doi: 10.1371/journal.pone.0070630 (PMC3734176; doi:10.1371/journal.pone.0070630)
Supplement: Table S10 — Demographics of study participants used in the cell purification. (PPTX) [file pone.0070630.s021.pptx]

## Slide 1
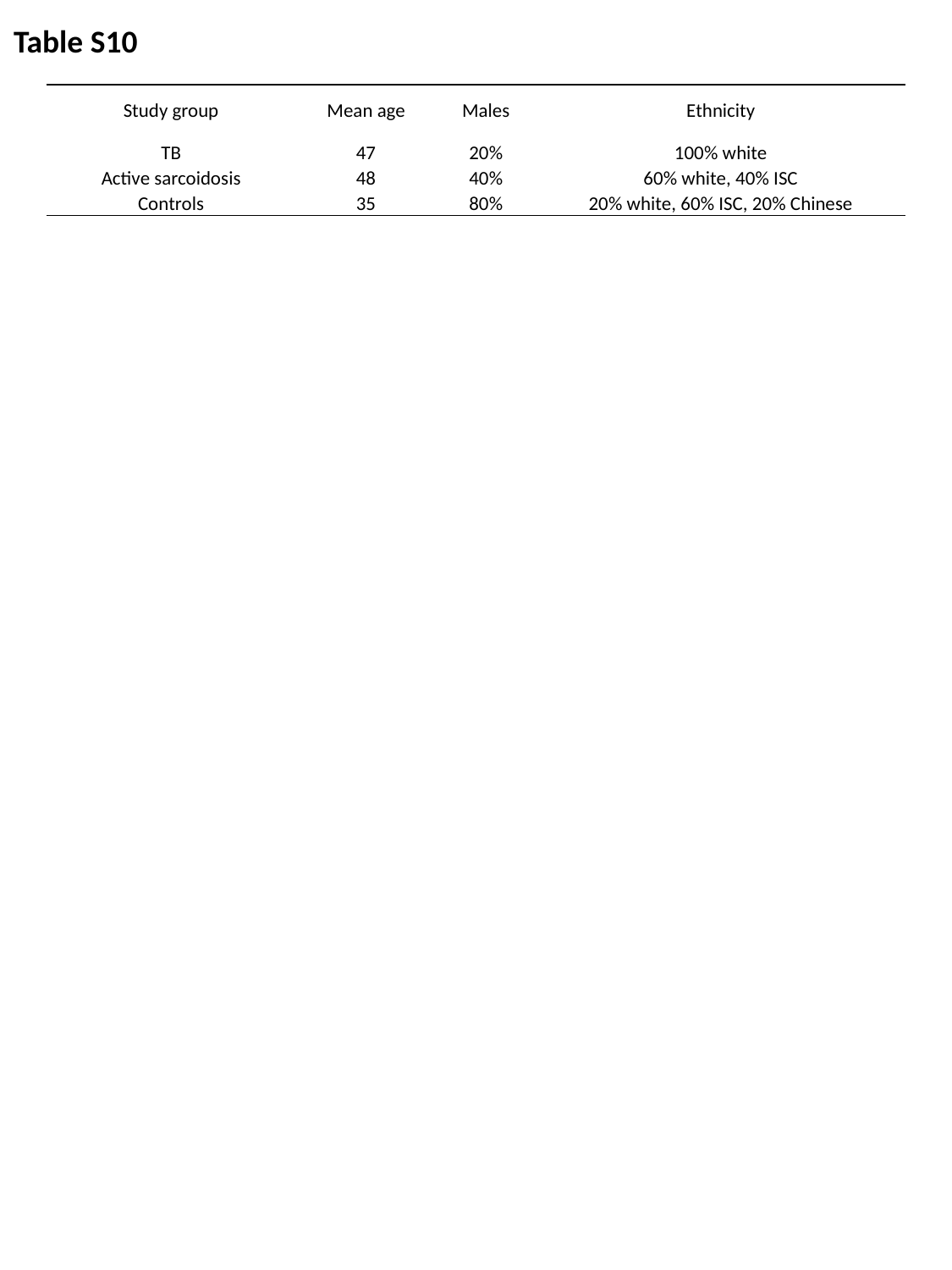

Table S10
| Study group | Mean age | Males | Ethnicity |
| --- | --- | --- | --- |
| TB | 47 | 20% | 100% white |
| Active sarcoidosis | 48 | 40% | 60% white, 40% ISC |
| Controls | 35 | 80% | 20% white, 60% ISC, 20% Chinese |
