# Supplementary material for: Transcriptional Blood Signatures Distinguish Pulmonary Tuberculosis, Pulmonary Sarcoidosis, Pneumonias and Lung Cancers
Source: PLoS One. 2013 Aug 5;8(8):e70630. doi: 10.1371/journal.pone.0070630 (PMC3734176; doi:10.1371/journal.pone.0070630)
Supplement: Table S11 — List of transcripts present in the IPA canonical pathways shown in Figure 5 . List of all transcripts by gene symbol that are present in the IPA canonical pathways: EIF2 signalling, interferon signalling, role of pattern recognition receptors in recognition of bacteria and viruses, and antigen presentation pathway. (PPTX) [file pone.0070630.s022.pptx]

## Slide 1
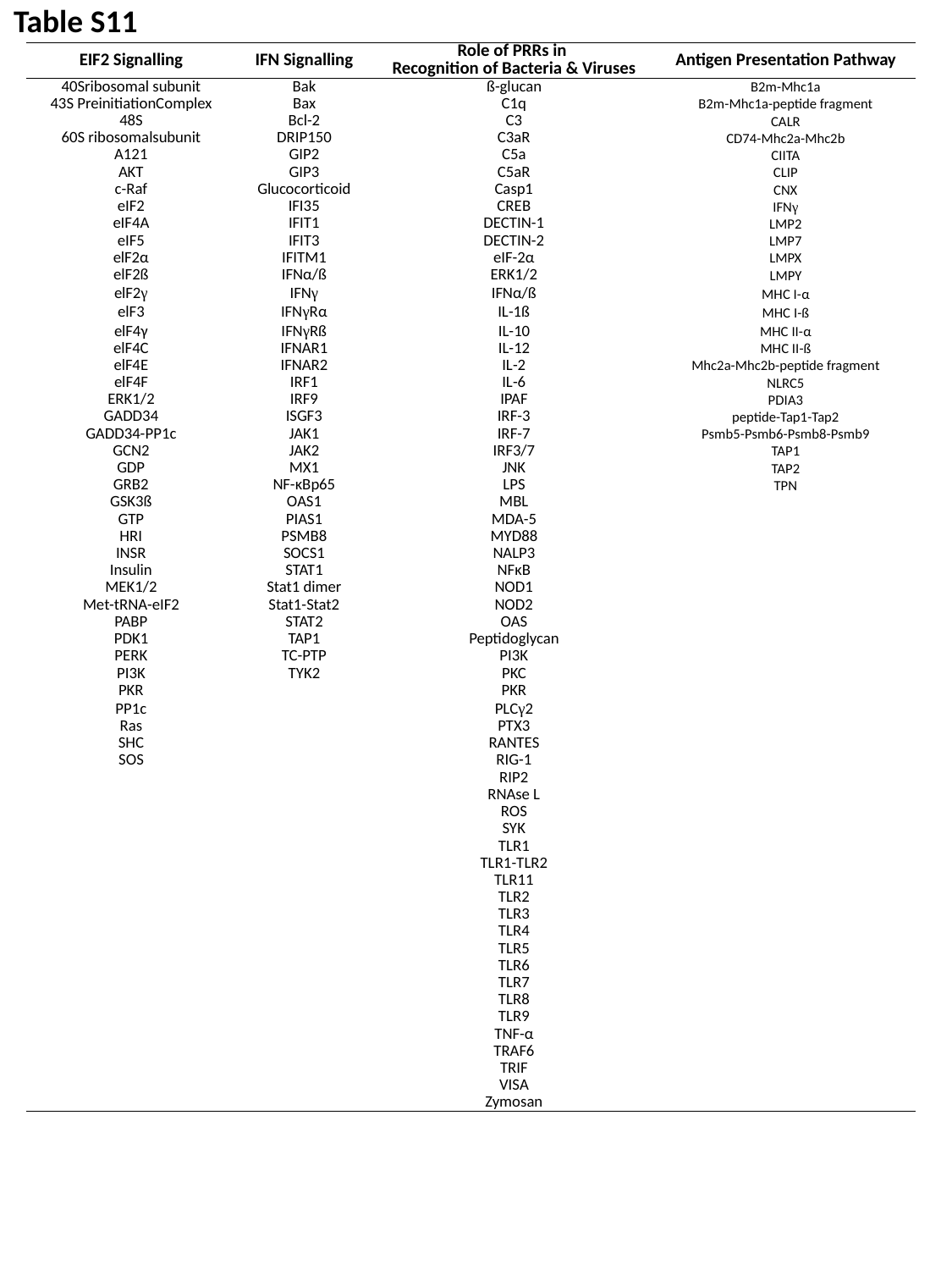

Table S11
| EIF2 Signalling | IFN Signalling | Role of PRRs in Recognition of Bacteria & Viruses | Antigen Presentation Pathway |
| --- | --- | --- | --- |
| 40Sribosomal subunit | Bak | ß-glucan | B2m-Mhc1a |
| 43S PreinitiationComplex | Bax | C1q | B2m-Mhc1a-peptide fragment |
| 48S | Bcl-2 | C3 | CALR |
| 60S ribosomalsubunit | DRIP150 | C3aR | CD74-Mhc2a-Mhc2b |
| A121 | GIP2 | C5a | CIITA |
| AKT | GIP3 | C5aR | CLIP |
| c-Raf | Glucocorticoid | Casp1 | CNX |
| eIF2 | IFI35 | CREB | IFNγ |
| eIF4A | IFIT1 | DECTIN-1 | LMP2 |
| eIF5 | IFIT3 | DECTIN-2 | LMP7 |
| elF2α | IFITM1 | eIF-2α | LMPX |
| elF2ß | IFNα/ß | ERK1/2 | LMPY |
| elF2γ | IFNγ | IFNα/ß | MHC I-α |
| elF3 | IFNγRα | IL-1ß | MHC I-ß |
| elF4γ | IFNγRß | IL-10 | MHC II-α |
| elF4C | IFNAR1 | IL-12 | MHC II-ß |
| elF4E | IFNAR2 | IL-2 | Mhc2a-Mhc2b-peptide fragment |
| elF4F | IRF1 | IL-6 | NLRC5 |
| ERK1/2 | IRF9 | IPAF | PDIA3 |
| GADD34 | ISGF3 | IRF-3 | peptide-Tap1-Tap2 |
| GADD34-PP1c | JAK1 | IRF-7 | Psmb5-Psmb6-Psmb8-Psmb9 |
| GCN2 | JAK2 | IRF3/7 | TAP1 |
| GDP | MX1 | JNK | TAP2 |
| GRB2 | NF-ĸBp65 | LPS | TPN |
| GSK3ß | OAS1 | MBL | |
| GTP | PIAS1 | MDA-5 | |
| HRI | PSMB8 | MYD88 | |
| INSR | SOCS1 | NALP3 | |
| Insulin | STAT1 | NFκB | |
| MEK1/2 | Stat1 dimer | NOD1 | |
| Met-tRNA-eIF2 | Stat1-Stat2 | NOD2 | |
| PABP | STAT2 | OAS | |
| PDK1 | TAP1 | Peptidoglycan | |
| PERK | TC-PTP | PI3K | |
| PI3K | TYK2 | PKC | |
| PKR | | PKR | |
| PP1c | | PLCγ2 | |
| Ras | | PTX3 | |
| SHC | | RANTES | |
| SOS | | RIG-1 | |
| | | RIP2 | |
| | | RNAse L | |
| | | ROS | |
| | | SYK | |
| | | TLR1 | |
| | | TLR1-TLR2 | |
| | | TLR11 | |
| | | TLR2 | |
| | | TLR3 | |
| | | TLR4 | |
| | | TLR5 | |
| | | TLR6 | |
| | | TLR7 | |
| | | TLR8 | |
| | | TLR9 | |
| | | TNF-α | |
| | | TRAF6 | |
| | | TRIF | |
| | | VISA | |
| | | Zymosan | |
